# How much GK is in the NKLM? A comparison between the catalogues of exam-relevant topics (GK) and the German National Competence-based Learning Objectives Catalogue for Undergraduate Medical Education (NKLM)

**DOI:** 10.3205/zma001086

**Published:** 2017-02-15

**Authors:** Olaf Fritze, Jan Griewatz, Elisabeth Narciß, Thomas Shiozawa, Annette Wosnik, Stephan Zipfel, Maria Lammerding-Koeppel

**Affiliations:** 1 Eberhard-Karls University, Competence Centre for University Teaching in Medicine, Baden-Wuerttemberg, Tuebingen, Germany; 2 University of Heidelberg, Faculty of Medicine Mannheim, Competence Centre of Final Year, Mannheim, Germany; 3 University of Tuebingen, Faculty of Medicine, Institute of Clinical Anatomy and Cell Analysis, Tuebingen, Germany; 4 University of Tuebingen, Faculty of Medicine, Dean's Office of Student Affairs, Tuebingen, Germany

**Keywords:** NKLM, GK, catalogues of exam-relevant topics, curriculum mapping, competence orientation, competency-based, medical education, change management

## Abstract

**Background: **The German National Competence-Based Learning Objectives for Undergraduate Medical Education (NKLM) being adopted in 2015 is designed to contribute to improve the quality of teaching and learning in medicine with respect to competence orientation. For departments, the coherence between teaching, assessment and the content of the catalogues of exam-relevant topics (GK) is a crucial factor. Before making use of the NKLM seriously in curricular development, many faculties demand more transparency regarding the representation in the NKLM of GK topics and in what aspects the NKLM exceeds the GK. Therefore, the aim of the study was to assign the NKLM competencies and objectives to the systematic GK terms, to reveal gaps in their congruence and to determine the percentage of agreement between GK and NKLM. Additionally, the distribution among the NKLM chapters (chap.), of GK content and further competencies relevant for medical practice were analysed.

**Methods:** The textual comparison of GK and NKLM was done by advanced students that were familiar with the NKLM from previous analyses. The comparison was done independently (keyword search, face validity), afterwards consented and matched with independent ratings of GK-2 and chapter 21 done by experts as well as with cross-references to the GK indicated in chapter 12, 13 and 15 of the NKLM. Detailed data is available online: www.merlin-bw.de/gk-nklm-abgleich.html.

**Results:** The degree of correspondence of the GK’s six preclinical parts with the NKLM ranges between 94% and 98%, with the clinical GK the degree of correspondence ranging between 84% and 88%. This demonstrates a consistently very high congruence of content. Only 6-16% of the content per GK part could not be assigned to NKLM equivalents. Regarding the distribution of GK content among NKLM chapters, the chapters with classic medical expertise (chapters 12, 13, 16, 17 as well as 20 and 21) show the highest correspondences. Practical medical skills (chapter 14b) can be found in the clinical GK “Health Disorders”. Doctor-patient interaction (chapter 14c) and medical scientific skills (chapter 14a) are represented only marginally in the GK. As expected, there were no equivalents to be found in the GK for the new professional roles for medical doctors (chapter 06-11).

**Discussion: **The results presented provide faculties with a useful and detailed data base to evaluate the NKLM more reliably, especially with respect to its relevance for exams. The increased transparency supports the implementation process of the NKLM by reducing content-related uncertainties of departments, invalidating sweeping arguments against the NKLM resulting from uncertainties and thereby minimizing resistance. At the same time a critical review process of the NKLM is encouraged.

## Zusammenfassung

**Hintergrund: **Der 2015 verabschiedete Nationale Kompetenzbasierte Lernzielkatalog Medizin (NKLM) soll dazu beitragen, die Qualität von Studium und Lehre in der Medizin in Bezug auf die Kompetenzorientierung zu verbessern. Für die Fächer ist die Kohärenz zwischen Lehre, Prüfungen und den Ausbildungsinhalten der Gegenstandskataloge (GK) entscheidend. Vor einer ernsthaften Auseinandersetzung mit dem NKLM und folgenden curricularen Änderungen fordern viele Fachvertreter mehr Transparenz dazu, wie die prüfungsrelevanten GK konkret im NKLM abgebildet sind und was der NKLM mehr bietet.

Ziel der Untersuchung ist deshalb, den systematischen Begriffen der GK die zugehörigen NKLM-Teilkompetenzen und -Lernziele zuzuordnen, Lücken in der Kongruenz aufzuzeigen und den prozentualen Grad der Übereinstimmung zwischen GK und NKLM zu ermitteln. Außerdem wird eruiert, wie sich die GK-Inhalte auf die NKLM-Kapitel (Kap.) verteilen und welche zusätzlichen berufsrelevanten Kompetenzen im NKLM, nicht aber in den GK enthalten sind.

**Methodik: **Der inhaltliche Vergleich zwischen GK und NKLM wurde von fortgeschrittenen Studierenden, die mit dem NKLM gut vertraut waren, unabhängig voneinander durchgeführt (Stichwortrecherche, Augenscheinprüfung), konsentiert und mit unabhängigen Expertenratings zu GK-2 und Kap. 21 sowie mit den in Kap. 12, 13 und 15 des NKLM angegebenen Querverweisen auf die GK abgeglichen. Die detaillierten Daten können unter www.merlin-bw.de/gk-nklm-abgleich.html heruntergeladen werden.

**Ergebnisse:** In den sechs vorklinischen Teilkatalogen liegt der jeweilige Übereinstimmungsgrad mit dem NKLM zwischen 94% und 98%, in den klinischen GK bei 84% bzw. 88%. Damit besteht durchgängig eine sehr hohe Kongruenz der Inhalte. Je nach GK-Teilkatalog konnten nur für 6-16 % der Inhalte keine passenden Zuordnungen im NKLM gefunden werden. Bezüglich der Verteilung der GK-Inhalte auf die NKLM-Kapitel zeigen diejenigen mit klassischem Fachwissen (Kap. 12, 13, 16, 17 sowie 20 und 21) die höchsten Übereinstimmungen. Die medizinisch-praktischen Fertigkeiten (Kap. 14b) finden sich im klinischen GK „Gesundheitsstörungen“ wieder. Die ärztliche Gesprächsführung (Kap. 14c) und medizinisch-wissenschaftliche Fertigkeiten (Kap. 14a) sind in den Teilkatalogen des GK nur marginal abgebildet. Für die neuen Arztrollen (Kap. 06 - 11) wurden erwartungsgemäß keine Entsprechungen im GK gefunden.

**Diskussion:** Mit den vorliegenden Ergebnissen ergibt sich eine hilfreiche detaillierte Datenbasis, die den Fakultäten mehr Sicherheit in der Einschätzung des NKLM gibt, insbesondere bezüglich seiner Examensrelevanz. Die höhere Transparenz begünstigt den NKLM-Implementierungsprozess, indem inhaltliche Verunsicherungen der Fächer reduziert, daraus folgende pauschale Argumente gegen den NKLM entkräftet und damit Widerstände abgebaut werden können. Gleichzeitig wird ein kritischer Review-Prozess des NKLM gefördert.

## Background

In 2015, the National Competence-based Learning Objectives Catalogue medicine (NKLM; [http://www.nklm.de accessed on 22.03.2016]) was passed by the Medical Faculty Association (MFT) as the official representative body of the medical faculties of Germany [4]. In adaptation to international developments, the NKLM is meant to improve the quality of study and teaching in medicine decisively with respect to competence orientation. The present catalogue is recommended to the faculties for testing and critical review until 2020 [4]. Meanwhile, discussions have started at many faculties about how to deal with the comprehensive NKLM. More and more deaneries of studies, as well as individual interested departments or professional groups are planning to compare their existing curriculum with the NKLM, to be able to make specific curricular changes after a status survey. In doing so, it is obvious that the resources required for implementing the NKLM are not to be underestimated and that resistances (e.g. of representatives of disciplines) are pre-programmed. These experiences were also made at the medical faculties of Baden-Wuerttemberg, which, in the scope of the BMBF sponsored joint project MERLIN [http://www.merlin-bw.de/ accessed on 18.07.2016] are cooperatively testing the implementation of the NKLM in teaching and assessment.

A frequently asked question by representatives of the disciplines concerns the relevance of the NKLM compared to the well-known and accepted set of rules for medical training used till now. In the introductory text to the NKLM (chapter 1), the importance of the NKLM is classified [http://www.nklm.de accessed on 22.03.2016]. It is emphasised that the NKLM describes the competence profile of the graduates in medicine, defines a core curriculum and consequently builds an orienting framework for medical education. On the one hand, it substantiates the regulations of the medical licensure act (ÄAppO; [https://www.gesetze-im-internet.de/_appro_2002/BJNR240500002.html accessed on 18.07.2016]), that allowed the faculties freedom to design. On the other hand, it includes the catalogues of exam-relevant topics (GK) of the Institute for Medical and Pharmaceutical Examination Questions (IMPP; [https://www.impp.de/internet/de/medizin/articles/gegenstandskataloge.html accessed on 10.12.2015]), which defines the essential medical expert knowledge and hence the knowledge relevant for examination by systematic lists of topics and terms. Thus, a nationwide consistent competence-oriented learning objectives catalogue has become available to the faculties for the first time, taking the relevant legal and textual reference scopes into account. However, a decisive aspect for a lot of representatives of the disciplines was the explicit agreement during the adoption of the NKLM, that the study and examination regulations of the faculties continue to remain binding as before [http://www.nklm.de accessed on 20.03.2016]. These regulations are usually oriented to the widely accepted GK of the IMPP, because the coherence between studies, examination and the educational contents of the GK is decisive for the departments:

1. The questions of the nationwide uniform written state examinations of the IMPP are put together from the GK’s list of terms;
2. the students align their learning with the GK;
3. furthermore the results of the state examinations are used as a marker for the quality of education.

Thus, confirming the finding: "Assessment drives curriculum and learning" [3], [10]. 

Therefore, a lot of representatives of the disciplines legitimately want to know in detail, which contents of the GK have been truly considered and mapped in the NKLM, before discussing the NKLM seriously:

- How well do the GK and the NKLM match?
- Which contents of the GK have been captured by which learning objectives of the NKLM, which have not?
- What does the NKLM add content-wise?

The desired transparency requires that the degree of match between GK and NKLM can be demonstrated concretely.

So far, the related GK contents have only been explicitly identified and made transparent in three chapters of the NKLM (chapters 12, 13, 15) [http://www.nklm.de accessed on 20.03.2016]. An independent, content-related comparison between the NKLM and the GK is vehemently denied by the representatives of the disciplines for the lack of resources and feasibility. Particularly as the NKLM consciously refrains from a classical department and organ assignment and therefore does not permit quick orientation. The present study is designed to fill this gap. The aim of the study is to assign the NKLM competencies and objectives to the systematic GK-1 and -2 terms, to search for gaps in their congruence and to determine the percentage of agreement between the GK and the NKLM. In a second question, it is examined, how the GK contents are distributed in the NKLM chapters and which additional, professionally relevant competencies are contained in the NKLM, but not in the GK. The results of this differentiated analysis should provide answers to the frequently asked questions and concerns of the departments.

## Methods

**Figure 1:**
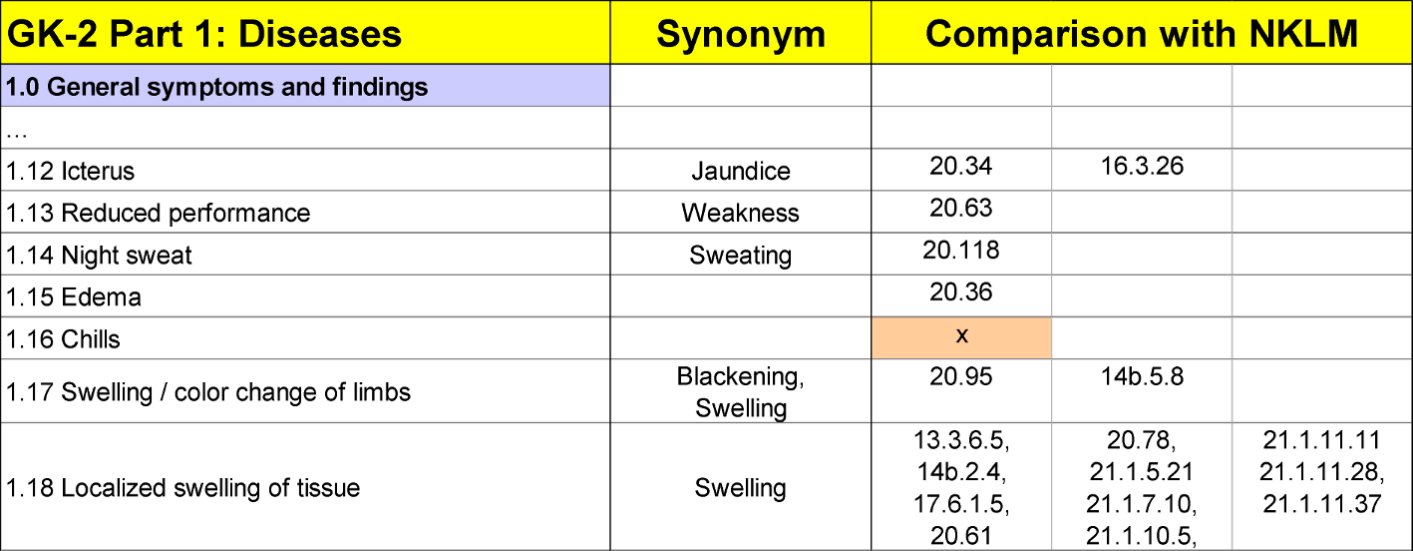
Comparison of GK lists of terms with NKLM contents. An exemplary part of the script illustrating the GK-NKLM comparison is shown. In the column “synonym” alternative terms are given, if comparable content was found in the NKLM but in different terminology. The NKLM matches were designated using the ID numbers of the NKLM competencies or learning objectives. For better clarity, in case of multiple matches, the IDs were split over three columns. In cases were no NKLM contents could be matched to GK terms, the relevant fields were highlighted in the NKLM column.

The comparative analysis between the GK and the NKLM was carried out based on the latest editions of the IMPP’s GK [https://www.impp.de/internet/de/medizin/articles/gegenstandskataloge.html accessed on 10.12.2015] as well as the passed NKLM version [http://www.nklm.de accessed on 20.03.2016]. The comparison was done by three advanced students of human medicine (10th semester). They were well acquainted with the NKLM from preliminary work and with the catalogues of exam-relevant topics due to the progress of their studies and examinations. Starting with the GK items, two students looked independently for content-related matches (keyword search, face validity through specific review of chapters with assumed content-related congruencies). In an Excel sheet, each GK term was assigned to the related identification number (ID) of the NKLM competencies or the NKLM learning objectives (see figure 1 (Fig. 1)). In case of clear differences in the terminology, the synonyms were documented. If no NKLM match was found for a GK term, the empty NKLM field was highlighted in colour (see figure 1 (Fig. 1)).

Both results were compared, differences and ambiguities were discussed and agreed upon. The calculation of an interrater reliability was not done. Later a third student checked a random selection of results for plausibility (esp.: matching of the synonyms and assignment). An additional check for correctness and completeness was done by checking against further sources:

1. with an independent expert rating for GK-2 Part 2 and chapter 21 (by two medical specialists experienced in dealing with the NKLM),
2. with the IMPP cross-references specified in chapters 12, 13 and 15 of the NKLM.

The entries were corrected if necessary. References that were not identified by the students were added. The tables were put together in a document (see Attachment 1) for internal use in the MERLIN project (support of the argumentation in the mapping process). In case further matches between the GK and the NKLM are found in the future, they will be added to the document. The latest version is available for download at the MERLIN homepage [http://www.merlin-bw.de/gk-nklm-abgleich.html accessed on 18.07.2016]. Based on the database described above, the coverage percentage of the GK contents by the NKLM was calculated (see table 1 (Tab. 1)). To represent the distribution of the GK contents on the entire learning objectives catalogue, the determined GK findings were counted out for each NKLM chapter and documented in tabular form (see table 2 (Tab. 2)).

## Results

### Which GK-1 and -2 concepts are present in the NKLM? 

Each GK term was checked, whether it could be identified contentwise at least once at the level of a competence or a learning objective in one or more chapters of the NKLM. Since both catalogues are different in their terminology, a potential uncertainty in the assignment could not be avoided. Nevertheless, matching ID numbers of the NKLM could be documented for almost every GK term (see figure 1 (Fig. 1)). In many cases, several matches could be found in different chapters of the NKLM. The latest tables with all details are available online in a document on the MERLIN homepage [http://www.merlin-bw.de/gk-nklm-abgleich.html accessed on 18.07.2016]. 

The degree of match was determined for each thematic GK sub-catalogue. Table 1 (Tab. 1) summarises the results sorted by sub-catalogues and shows the coverage percentage. In the six thematic sub-catalogues of the pre-clinical stage of studies, the respective degree of match with the NKLM lies between 94% and 98%. In both parts of the clinical GK (GK-2 Part 1 and Part 2), respectively 84% and 88% of the terms are present in an NKLM competency or learning objective as well. This demonstrates a consistently very high congruence of content.

The GK contents not found in the NKLM are of special relevance. These were highlighted in the table (see Attachment 1). In this way, they can be quickly identified specifically by the representatives of the disciplines and discussed critically. Depending on the GK catalogue, no matches in the NKLM could be found for only 6-16% of the contents. This results from the percentages of the matches in table 1 (Tab. 1).

#### Among which NKLM chapters are the contents of GK-1 and GK-2 distributed?

In order to show, to what extent the NKLM goes beyond the topic catalogues of IMPP, the NKLM contents (see table 2 (Tab. 2)) were assigned to the GK-1 and -2 contents. As expected, the chapters, displaying the classical knowledge (chapters 12, 13, 16, 17 as well as 20 and 21) show high correspondence with the GK contents. For the practical medical skills (chapter 14b), there are numerous matches in the GK-2 Part 1 "Health disorders" too. Doctor-patient interaction (chapter 14c) and medical scientific skills, on the other hand, are represented only marginally in the catalogues of the GK. As expected too, there were no equivalents to be found in the GK with one exception, for the newly formulated professional roles for medical doctors (chapter 06-11).

## Discussion

In the present study, the contents of the catalogues of the IMPP (GK) were compared with the NKLM in detail. The differentiated comparison shows a very high content-related matching of the GK with the NKLM. Since the essential and examination-relevant medical knowledge of the IMPP object catalogues is also found in the NKLM chapters, the results confirm the statement of the NKLM introductory text ("The NKLM is not opposed to the IMPP GK-1 and 2." [http://www.nklm.de accessed on 20.03.2016]). The present study provides recent figures for supporting this position and arguments for the discussion with the refusing or uncertain representatives of the disciplines.

The different terminology in GK and NKLM and the frequent use of collective terms in the NKLM makes it difficult to make a clear decision about correspondences. The specification of the equivalent synonyms used in this study can contribute to a higher transparency. No matching assignments in the NKLM could only be found for a few GK contents. This result could perhaps become even better by selecting a more comprehensive searching strategy. Since the catalogues were not compared in small steps (objective by objective), but instead by looking over the chapters with suspected textual relationship or via keyword search, congruent contents could have been overlooked. Moreover, considering the different terminology, it cannot be ruled out that the terms were assigned incorrectly by mistake. Furthermore, selected GK contents could have intentionally been left out to favour a reduction of content while drafting the NKLM or could also have not been considered by mistake. The fact that only two respectively three students carried out the GK-NKLM comparison seems to be acceptable for resource reasons. These are especially qualified students, who were facing their year of internship and whose ratings were also validated by other sources, including an expert rating. With respect to the high matching between the GK and the NKLM, the results of this study in all provide a convincing orientation and adequate surety for teaching and examination for the departments - an argument, which according to our experience, can effectively be used while implementing the NKLM.

An additional advantage of the NKLM was uncovered by the comparison. The expert knowledge is not only mentioned in systematic terms lists (as in the GK), but also operationalised in competencies and learning objectives so that the level of competency acquisition is defined at a specific milestone. This facilitates the faculties to design their own learning objective catalogues and safeguards mainly the equivalence check of training at different locations. It is an advantage that by this comparison the few GK contents missing in the NKLM could be identified. In this way, the representatives of the departments can easily decide, whether the missing terms should be added or whether, as per exemplary teaching [https://www.gesetze-im-internet.de/_appro_2002/BJNR240500002.html accessed on 18.07.2016] these can be avoided.

This data helps to achieve a high transparency, which is from experience required by the departments when attempting implementation [9]. This transparency is also relevant for the pending review process of the NKLM, since the data can act as basis for a content-related quality assurance and critical review.

Moreover, it becomes clear from the results, where the NKLM clearly exceeds the expert knowledge of the object catalogue in its definition of professionally relevant educational objectives. The NKLM adds important professional roles for medical doctors, which were referred to as highly relevant for professional practice by the university teachers in national and international surveys [12], [13], [14]. It also adds "social, communicative, clinical-practical and scientific competencies“ [4], [17] required by the science council. The appearance of various GK contents in different NKLM chapters reflects a high degree of networking between the three structuring pillars of the NKLM. Contrary to the frequent concerns of the departments, it becomes clear that medical competencies are not to be imparted isolated as "add-ons", but instead embedded in practical contexts, woven with guiding symptoms and disease patterns or in combination with theoretical concepts and principles [7].

The current project is an example, how the NKLM can be linked with other catalogues (here, with the IMPP object catalogues). In a similar way, the specific learning objectives catalogues of the specialist medical societies (e.g. [1], [2], [11], [14], [16]) and faculties, guidelines, etc. can also be linked. This enables a high degree of transparency and matching at different levels.

Overall, the results presented provide faculties with a useful and detailed data base to evaluate the NKLM more reliably, especially with respect to its relevance for examinations. At the same time a critical review process of the NKLM is encouraged. In addition, the increased transparency supports the implementation process of the NKLM by reducing content-related uncertainties of departments, invalidating sweeping arguments against the arguments resulting from uncertainties and thereby minimizing resistance.

## Competing interests

The authors declare that they have no competing interests.

## Supplementary Material

Complementary document with the detailed data of the GK-NKLM adjustment - only in german

## Figures and Tables

**Table 1:**
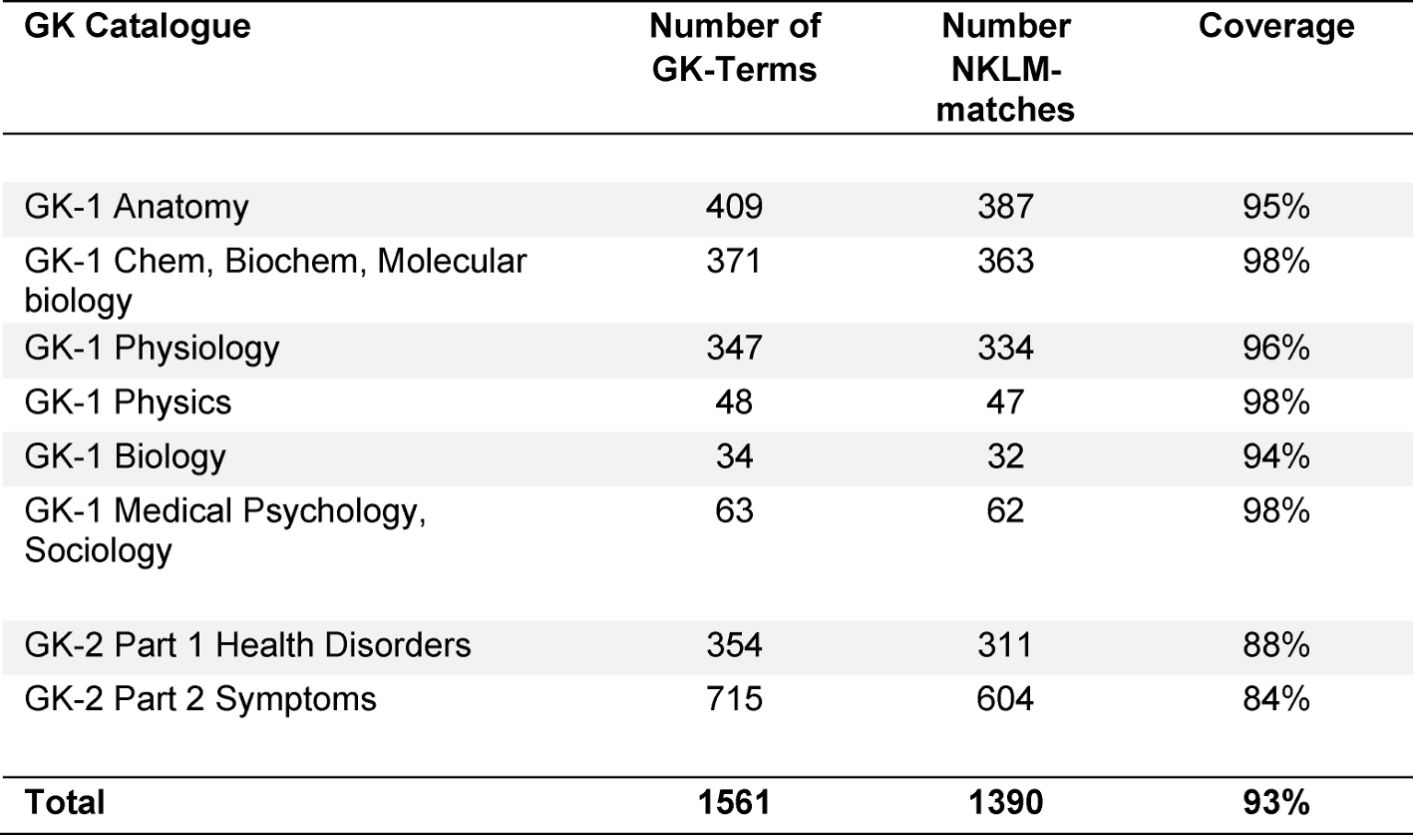
Quantitative accordance between GK-1 / GK-2 and NKLM. The columns show the total number of terms in the respective GK catalogues, the number of matches within the NKLM competencies / learning objectives („NKLM-matches“) and the percentage of agreement („coverage“).

**Table 2:**
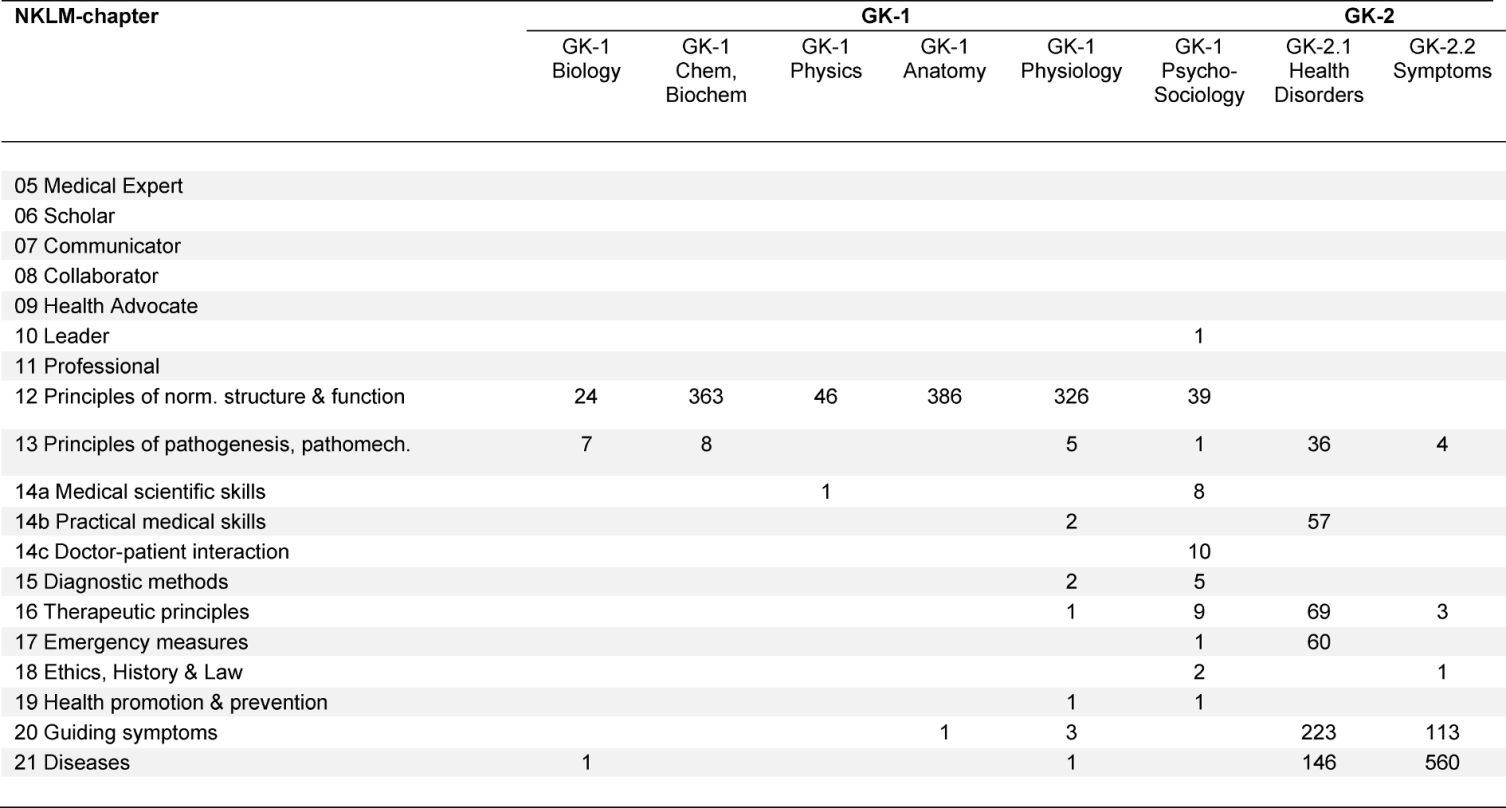
Distribution of matches from GK-1 and GK-2 to NKLM-chapters. The table indicates the number of GK-terms with one or more equivalents to be found in a NKLM-chapter.

## References

[1] Breuer G, Ahlers O, Beckers S, Breckwoldt J, Böttiger B, Eichler W, Frank A, Hahnenkamp K, Hempel G, Koppert W, Meyer O, Mönk S, Schaumberg A, Schmidt G, Schneier G, Sopka S, Schüttler J (2015). Nationaler Lernzielkatalog Anästhesiologie" mit fachspezifischen Aspekten der Bereiche Intensivmedizin, Notfall- und Schmerzmedizin. Kommission Studentische Lehre und Simulatortraining der Deutschen Gesellschaft für Anästhesiologie und Intensivmedizin (DGAI) - Grundlage einer lebenslangen Lernspirale und Basis der aktuellen Musterweiterbildungsordnung. Anästh Intensivmed.

[2] Dugas M, Röhrig R, Stausberg J, GMDS-Projektgruppe MI-Lehre in der Medizin" (2012). Welche Kompetenzen in Medizinischer Informatik benötigen Ärztinnen und Ärzte? Vorstellung des Lernzielkatalogs Medizinische Informatik für Studierende der Humanmedizin. GMS Med Inform Biom Epidemiol.

[3] Epstein RM (2007). Assessment in Medical Education. N Engl J Med.

[4] Fischer MR, Bauer D, Mohn K, NKLM-Projektgruppe (2015). Finally Finished! National Competence Based Catalogues of Learning Objectives for Undergraduate Medical Education (NKLM) and Dental Education (NKLZ) ready for trial. GMS Z Med Ausbild.

[5] Fritze O, Boecker M, Gornostayeva M, Durante S, Griewatz J, Öchsner W, Wosnik A, Lammerding-Köppel M (2014). Kompetenzorientiertes Curriculummapping im MERLIN-Projekt: eine Online-Datenbank als Tool zur gezielten curricularen Weiterentwicklung. http://dx.doi.org/10.3205/14gma255.

[6] Griewatz J, Wiechers S, Ben-Karacobanim H, Lammerding-Koeppel M (2016). Medical teachers' perception of professional roles in the framework of the German National Competence-Based Learning Objectives for Undergraduate Medical Education (NKLM) – a multi-centre study. Med Teach.

[7] Harden RM (2001). AMEE guide no. 21: Curriculum mapping: A tool for transparent and authentic teaching and learning. Med Teach.

[8] Jilg S, Möltner A, Berberat P, Fischer MR, Breckwoldt J (2015). Wie bewerten im Krankenhaus tätige Ärztinnen und Ärzte die Bedeutung der Rollen-definierenden Kompetenzen des CanMEDS-Modells und ihre Umsetzung für die Ausbildung im Praktischen Jahr?. GMS Z Med Ausbild.

[9] Lammerding-Köppel M, Giesler M, Gornostayeva M, Narciss E, Wosnik A, Zipfel S (2016). Monitoring und Analyse des Change-Prozesses beim Curriculummapping zum Nationalen Kompetenzorientierten Lernzielkatalog Medizin (NKLM) an vier Medizinischen Fakultäten. Teil I: Förderliche Ressourcen und Strukturen, Teil II: Motivierung der Lehrenden im Prozess. GMS J Med Educ.

[10] Newable D, Jaeger K (1983). The effect of assessment and examination on the learning of medical students. Med Educ.

[11] Preisser AM, Angerer P, Hildenbrand S, Letzel S (2015). Neuer Lernzielkatalog für das Fach Arbeitsmedizin. ASU.

[12] Rademakers JJ, de Rooy N, Cate OT (2007). Senior medical students' appraisal of CanMEDS competencies. Med Educ.

[13] Ringsted C, Hansen TL, Davis D, Scherpbier A (2006). Are some of the challenging aspects of the CanMEDS roles valid outside Canada?. Med Educ.

[14] Stutsky BJ, Singer M, Renaud R (2012). Determining the weighting and relative importance of CanMEDS roles and competencies. BMC Res Notes.

[15] Walcher F, Dreinhöfer KE, Obertacke U, Waydhas C, Josten C, Rüsseler M (2008). Entwicklung des Lernzielkatalogs "Muskuloskelettale Erkrankungen, Verletzungen und traumatische Notfälle" für Orthopädie-Unfallchirurgie im Medizinstudium. Unfallchirurg.

[16] Weidner K, Herrmann-Lingen C, Herzog W, Jünger J, Kruse J, Zipfel S, Köllner V (2015). Lernziele der Psychosomatischen Medizin und Psychotherapie vor dem Hintergrund des Nationalen kompetenzbasierten Lernzielkataloges Medizin (NKLM). Z Psychosom Med Psychother.

[17] Wissenschaftsrat (WR) (2014). Empfehlungen zur Weiterentwicklung des Medizinstudiums in Deutschland auf Grundlage einer Bestandsaufnahme der humanmedizinischen Modellstudiengänge.

