# Supplementary material for: How much GK is in the NKLM? A comparison between the catalogues of exam-relevant topics (GK) and the German National Competence-based Learning Objectives Catalogue for Undergraduate Medical Education (NKLM)
Source: GMS J Med Educ. 2017 Feb 15;34(1):Doc9. doi: 10.3205/zma001086 (PMC5327656; doi:10.3205/zma001086)
Supplement: Complementary document with the detailed data of the GK-NKLM adjustment - only in german [file JME-34-9-s-001.pdf]

EBERHARD KARLS
UNIVERSITÄT
TÜBINGEN

MERLIN
Medical Education Research -
Lehrforschung im Netz BW

NKLM und Gegenstands-Kataloge GK-1 und GK-2 im Vergleich

Stand: 18.7.2016

Olaf Fritze, Maria Lammerding-Köppel

© Kompetenzzentrum für Hochschuldidaktik in Medizin Baden-Württemberg

GEFÖRDERT VOM
Bundesministerium
für Bildung
und Forschung

Impressum

Autoren:
Olaf Fritze,
Maria Lammerding-Köppel

Herausgeber:
Dr. med. M. Lammerding-Köppel, MME
Kompetenzzentrum für Hochschuldidaktik in Medizin Baden-Württemberg

Kontakt:
Kompetenzzentrum für Hochschuldidaktik in Medizin Baden-Württemberg
Medizinische Fakultät der Eberhard Karls Universität Tübingen
Elfriede-Aulhorn-Str. 10
D-72076 Tübingen
Tel.: +49 (0)7071-29 77 974 / -60
Fax: +49 (0)7071-29 52 18
E-Mail: medizindidaktik@med.uni-tuebingen.de

18.7.2016

© Kompetenzzentrum für Hochschuldidaktik in Medizin Baden-Württemberg

INHALTSVERZEICHNIS **Seite**

1. METHODIK 4
   1.1. Basis der Datenerhebung 4
   1.2. Vorgehen 4
2. PROZENTUALE ABDECKUNG DER GK-INHALTE IM NKLM 5
3. NKLM IM VERGLEICH ZUM GK-1 6
   3.1. Biologie für Mediziner 7
   3.2. Chemie für Mediziner und Biochemie/Molekularbiologie 7
   3.3. Physik für Mediziner 14
   3.4. Anatomie 15
   3.5. Physiologie 23
   3.6. Medizinische Psychologie und Medizinische Soziologie 29
4. NKLM IM VERGLEICH ZUM GK-2 31
   4.1. GK-2 Teil 1: Gesundheitsstörungen 32
   4.2. GK-2 Teil 2: Krankheitsbilder 38

1. Methodik

1.1. Basis der Datenerhebung

IMPP-Gegenstandskatalog (IMPP-GK-1):

- Teilkatalog „Biologie für Mediziner“ Auflage von Januar 2014
- Teilkatalog „Chemie für Mediziner und Biochemie/Molekularbiologie“ Auflage von Januar 2014
- Teilkatalog „Physik für Mediziner“ Auflage von Mai 2014
- Teilkatalog „Anatomie“ Auflage von Januar 2014
- Teilkatalog „Physiologie“ Auflage von Januar 2014
- Teilkatalog „Grundlagen der Medizinischen Psychologie und der Medizinischen Soziologie“ 2. Auflage von März 2010

IMPP-Gegenstandskatalog (IMPP-GK-2) 4. Auflage von Dezember 2013

1.2. Vorgehen

2 Studierende der Humanmedizin (10. Fachsemester) suchten zunächst unabhängig voneinander nach inhaltlichen Übereinstimmungen (Stichwortrecherche, Augenscheinprüfung durch gezielte Sichtung von Kapiteln mit vermuteten inhaltlichen Kongruenzen). In einer Excel-Tabelle wurden jedem GK-Begriff die zugehörigen Identifikationsnummern (ID) für NKLM-Teilkompetenzen oder NKLM-Lernziele zugeordnet. Bei deutlichen Unterschieden in der Terminologie wurden die Synonyme dokumentiert. Wurden zu einem GK-Begriff keine NKLM-Entsprechungen gefunden, so wurde das leere NKLM-Feld farblich hervorgehoben.

Beide Ergebnisse wurden verglichen, bei Unterschieden oder Unsicherheiten diskutiert und konsentiert. Ein dritter Studierender (8. Fachsemester) prüfte die Ergebnisse im Nachgang stichprobenartig auf Plausibilität. Eine zusätzliche Prüfung auf Richtigkeit und Vollständigkeit wurde durch den Abgleich mit weiteren Quellen realisiert:

(1) mit einem unabhängigen Expertenrating zu GK-2 Teil 2 und NKLM-Kapitel 21,

(2) mit den in den NKLM-Kapiteln 12, 13 und 15 angegebenen IMPP-Querverweisen.

Nicht von den Studierenden identifizierte Fundstellen wurden ergänzt.

Dieses Skript soll auch weiterhin vervollständigt werden, die aktuellste Version steht als Download unter www.merlin-bw.de/gk-nklm-abgleich.html zur Verfügung.

Bei vielen NKLM-Fundstellen wurden die entsprechenden ID zugunsten einer besseren Übersichtlichkeit in 3 Spalten aufgeteilt.

Die Verfasser geben keine Garantie für Vollständigkeit und Richtigkeit der Vergleichsergebnisse.

2. Prozentuale Abdeckung der GK-Inhalte im NKLM

In der nachfolgenden Tabelle ist zum Überblick dargestellt, wie viele Themen die einzelnen Gegenstandskataloge beinhalteten, wie viele dieser Inhalte im NKLM wieder gefunden wurden und welcher prozentualen Abdeckung dies entspricht:

Gegenstandskatalog	Anzahl der GK-Begriffe	NKLM-Treffer	Abdeckung
GK-1 Anatomie	409	387	95%
GK-1 Chem&Biochem	371	363	98%
GK-1 Physiologie	347	334	96%
GK-1 Physik	48	47	98%
GK-1 Biologie	34	32	94%
GK-1 Psycho-Sozio	63	62	98%
GK-2 Teil1	354	311	88%
GK-2 Teil2	715	604	84%
**Gesamt**	1561	1390	93%

3. NKLM im Vergleich zum GK-1

3.1. Biologie für Mediziner

GK Biologie		Abgleich mit NKLM		
**1**	**Allgemeine Zellbiologie, Zellteilung und Zelltod**			
1.1	Zellbegriff und zelluläre Strukturelemente	12.3.1	12.3	
1.2	Zellmembran	12.3.1, 12.3.1.4	12.3.1.2, 12.3.1.4	12.3.2.1, 12.3
1.3	Zellkern	12.3.1, 12.3.1.4	12.2.5, 12.3.1.3	12.2.5.5, 12.3, 12.3.1.1
1.4	Zytoplasma, Zytosol	12.3.1, 12.3.1.4	12.2.5.5.7, 12.3.1.3	12.3, 12.3.1.1
1.5	Ribosomen	12.3.1, 12.3.1.4	12.2.5.7, 12.3.1.3	12.3, 12.3.1.1
1.6	Endoplasmatisches Retikulum	12.3.1, 12.3.1.4	12.3, 12.3.1.3	12.3.1.1
1.7	Golgi-Komplex (Golgi-Apparat)	12.3.1, 12.3.1.4	12.3, 12.3.1.3	12.3.1.1
1.8	Lysosomen	12.3.1, 12.3.1.4	12.3, 12.3.1.3	12.3.1.1
1.9	Stoffabgabe	12.3.1, 12.3.1.4	12.3, 12.3.1.3	12.3.1.1
1.10	Stoffaufnahme	12.3.1, 12.3.1.4	12.3, 12.3.1.3	12.3.1.1
1.11	Peroxisomen	12.3.1, 12.3.1.4	12.3, 12.3.1.3	12.3.1.1
1.12	Mitochondrien	12.3.1, 12.3.1.4	12.3, 12.3.1.3	12.3.1.1
1.13	Zytoskelett	12.3.1, 12.3.1.4	12.3, 12.3.1.3	12.3.1.1
1.14	Zellzyklus und Zellteilung (Mitose)	12.3.3	12.2.5.5	12.2.5.6
1.15	Meiose (Reifeteilung)	12.3.3, 12.3.3.2	12.2.5.5	12.2.5.6
1.16	Zelltod	12.3.5		
1.17	Zellkommunikation und Signaltransduktion	12.3.5		
**2**	**Genetik/Grundlagen der Humangenetik**			
2.1	Organisation und Funktion eukaryontischer Gene	12.2.5	12.2.5.5	
2.2	Chromosomen des Menschen	12.2.5	12.2.5.5	
2.3	Formale Genetik	12.2.5	12.2.5.5	
2.4	Gonosomen, Geschlechtsbestimmung und -differenzierung	12.2.5	12.2.5.5	12.7.1.1
2.5	Mutationen	12.2.5	12.2.5.5	
2.6	Klonierung und Nachweis von Genen bzw. Genmutationen	12.2.5		
2.7	Entwicklungsgenetik	x		
2.8	Populationsgenetik	x		
2.9	Genetische Evolution	12.2.5		
**3**	**Grundlagen der Mikrobiologie und Ökologie**			
3.1	Grundlagen der mikrobiologischen Ökologie und der Infektionen	13.2.6		
3.2	Morphologische Grundformen der Bakterien	13.2.6		
3.3	Aufbau und Morphologie der Bakterienzelle (Procyte)	13.2.6		
3.4	Stoffwechsel und Vermehrung der Bakterien	13.2.6		
3.5	Bakteriengenetik	13.2.6		
3.6	Pilze	13.2.6		
3.7	Viren	13.2.6		
3.8	Prionen	21.1.10.40		

3.2. Chemie für Mediziner und Biochemie/Molekularbiologie

GK Chem. & Biochem. & Molbio.		Synonym	Abgleich mit NKLM		
**1**	**Aufbau der Materie**				
**1.1**	**Atome, chemische Elemente**		12.2.1.9	12.2.2.1	12.2.2.2, 12.2.2.3
1.1.1	Begriffe		12.2.2		
1.1.2	Atomkern (s. a. GK Physik 3.1)	Aufbau Elemente/ Materie	12.2.2		
1.1.3	Radioaktivität(s. a. GK Physik Kap. 8)		12.2.1		
1.1.4	Elektronenhülle	Aufbau Elemente/ Materie	12.2.2		
1.1.5	Periodensystem	periodisch; Funktion von Elektrolyten und Spurenelementen	12.2.2	12.2.3.7	
**1.2**	**Moleküle, chemische Bindungen**		12.2.2.1	12.4.2.6	12.12.2.1
1.2.1	Ionenbindung	Materie aus Molekülen	12.2.2		
1.2.2	Atombindung		12.2.2		
1.2.3	Polarität von Molekülen	Aufbau, periodische Eigenschaften und Stabilität der Elemente	12.2.2.1		
1.2.4	schwache Wechselwirkungen		12.2.2		
1.2.5	Metallkomplexe		12.2.2		
1.2.6	metallische Bindung	Aufbau, periodische Eigenschaften und Stabilität der Elemente	12.2.2.1		
1.2.7	Bindungstypen in biochemisch relevanten Verbindungen	chemische Struktur	12.2.2		
**1.3**	**Heterogene Stoffgemische**	Eigenschaften von Gasen	12.2.1.7	12.2.2.2	12.2.2.4, 12.4.2.6
1.3.1	Aggregatzustände (s. a. GK Physik 4.4 und 4.6)	Phasengleichgewichte	12.2.2		
1.3.2	Begriffe	stoffliche Eigenschaften der Materie unkl. Phasen, -gleichgewichte und Gleichgewichte	12.2.2.2		
1.3.3	heterogene Gleichgewichte (s. a. GK Physik 4.6)	Aufbau, Eigenschaften und Funktion von biologischen Membranen	12.2.2, 12.14.2.8, 12.16.3.3	12.3.1.2, 12.16.3.1	12.3.2.1, 12.3.1.2, 12.16.3.2
1.3.4	Trennverfahren		12.2.2		
**2**	**Chemisch-analytische Verfahren in der Biochemie und Medizin**				
**2.1**	**Elektromagnetische Strahlung (s. a. GK Physik 6.4)**	Bedeutung von Schwingungen und Wellen in der Medizin	12.2.2, 12.2.1.4	12.2.2.3	
**2.2**	**Begriffe (s.a. GK Physik 7.4)**		x		
**2.3**	**NMR-Spektroskopie (s. a. GK Physik 5.9)**		x		
**2.4**	**Infrarotspektroskopie**		x		
**2.5**	**UV/VIS-Spektroskopie (s.a. GK Physik 7.4)**		x		
**2.6**	**Massenspektrometrie**		x		
**3**	**Chemische Reaktionen**				
**3.1**	**Chemische Gleichungen**		12.2.2.5		
3.1.1	Begriffe, Definitionen		12.2.2		
3.1.2	Stöchiometrie		12.2.2		
**3.2**	**Thermodynamische Grundlagen**		12.2.1.1	12.2.2.2	12.2.2.5
3.2.1	Begriffe, Definitionen		12.2.2		
3.2.2	Energetik chemischer Reaktionen		12.2.2		
3.2.3	chemisches Gleichgewicht		12.2.2		
**3.3**	**Grundlagen der Kinetik**	Biokatalyse	12.2.4	12.2.2.2	12.2.2.5
3.3.1	Begriffe, Definitionen		12.2.2		
3.3.2	Energieprofil		12.2.2		
3.3.3	Katalyse		12.2.2		
3.3.4	Zeitgesetze		12.2.2		
**3.4**	**Säure-Base-Reaktionen, Puffer (s. a. 21.1 und GK Physiol. 5.10)**	Mechanismen der Regulation des zellulären pH-Wertes	12.3.2.4, 12.12.2.1	12.2.2.6, 12.14.3.5	12.4.2.6, 12.16.2.6
3.4.1	Begriffe, Definitionen		12.2.2	12.2.2.6	
3.4.2	dissoziationsabhängige Größen		12.2.2	12.2.2.6	
3.4.3	Säure-Base-Reaktionen		12.2.2	12.2.2.6	
3.4.4	Puffersysteme		12.2.2	12.2.2.6	
3.4.5	Beispiele		12.2.2	12.2.2.6	
**3.5**	**Redox-Reaktionen**		12.6.1.7, 12.12.2.4	12.2.2.6	12.6.1.8, 12.6.2.1
3.5.1	Begriffe, Definitionen		12.2.2	12.2.2.6	
3.5.2	Redox-Gleichungen		12.2.2	12.2.2.6	
3.5.3	elektrochemische Zellen		12.2.2	12.2.2.6	
3.5.4	Beispiele		12.2.2	12.2.2.6	
**3.6**	**Reaktionen von Salzen**		12.2.2.6		
3.6.1	Salzlösungen		12.2.2	12.2.2.6	
3.6.2	Beispiele		12.2.2	12.2.2.6	
**3.7**	**Reaktionen von Metallkomplexen**		12.2.2.6	12.6.1.8	12.12.2.1
3.7.1	Eigenschaften		12.2.2		
3.7.2	typische Reaktionen		12.2.2		
**4**	**Reaktionen einfacher und substituierter Kohlenstoffverbindungen**				
**4.1**	**Bindungsverhältnisse**	chem. Struktur und Reaktionen biochem. Funktioneller Gruppen	12.2.2.7		
**4.2**	**Reaktionstypen**		12.2.2.7		

**5**	**Grundstrukturen**	funktionelle Gruppen und monomere Bausteine komplexer Biomoleköle	12.2.3.1	12.2.2.7	
**5.1**	**Offenkettige Kohlenwasserstoffe**	biochem	12.2.2	12.2.2.7	
**5.2**	**Alicyclische Verbindungen**	biochem	12.2.2	12.2.2.7	
**5.3**	**Aromaten**	biochem	12.2.2	12.2.2.7	
**5.4**	**Heterocyclen**	biochem	12.2.2	12.2.2.7	
**6**	**Funktionelle Gruppen**	funktionelle Gruppen und monomere Bausteine komplexer Biomoleküle	12.2.3.1	12.2.2.7	
**6.1**	**Alkohole, Phenole, Chinone, Ether**	biochem	12.2.2	12.2.2.7	
**6.2**	**Verbindungen mit N**	biochem	12.2.2	12.2.2.7	
**6.3**	**Verbindungen mit S**	biochem	12.2.2	12.2.2.7	
**6.4**	**Aldehyde und Ketone**	biochem	12.2.2	12.2.2.7	
**6.5**	**Carbonsäuren, Carbonsäurederivate**	biochem	12.2.2	12.2.2.7	
**6.6**	**Hydroxy- und Oxocarbonsäuren**	biochem	12.2.2	12.2.2.7	
**6.7**	**„Anorganische“ Säuren und ihre Derivate**	biochem	12.2.2	12.2.2.7	
**7**	**Stereochemie**		12.2.2.7	12.2.2.8	
**7.1**	**Isomerien**		12.2.2.7		
7.1.1	Begriffe, Definitionen	chem. Struktur und Reaktionen biochem. Funktioneller Gruppen erklären; Bedeutung der räumlichen Anordnung funktioneller Gruppen	12.2.2.7	12.2.2.8	
7.1.2	Anwendungen		12.2.2.7	12.2.2.8	
**7.2**	**Enantiomere**			12.2.2.7	
7.2.1	Eigenschaften	chem. Struktur und Reaktionen biochem. Funktioneller Gruppen; Bedeutung der räumlichen Anordnung funktioneller Gruppen	12.2.2.7	12.2.2.8	
7.2.2	Nomenklatur		12.2.2.7	12.2.2.8	
7.2.3	Anwendungen		12.2.2.7	12.2.2.8	
**8**	**Medizinisch relevante Werkstoffe/Biomaterialien**		12.2.2.7		
**8.1**	**Metalle**		12.2.2	12.2.2.7	
**8.2**	**Keramische Materialien**		12.2.2	12.2.2.7	
**8.3**	**Polymere**		12.2.2	12.2.2.7	
**8.4**	**Anwendungen**		12.2.2	12.2.2.7	
**9**	**Struktur und Eigenschaften von Aminosäuren, Peptiden und Proteinen**		12.2.2.7, 12.6.2.4	12.2.3.1, 12.10.3.6	12.2.3.3, 12.15.3.4
**9.1**	**Aminosäuren**		12.2.2.7	12.2.3.1	
9.1.1	Klassifizierung, Struktur		12.2.3	12.2.2.7	12.2.3.1
9.1.2	Eigenschaften		12.2.3	12.2.2.7	12.2.3.1
9.1.3	Reaktionen		12.2.3	12.2.2.7	12.2.3.1
**9.2**	**Peptide und Proteine**	Struktur und Funktionsweise von Enzymen	12.2.4.1	12.2.2.7	12.2.3.1
9.2.1	Begriffe		12.2.3	12.2.2.7	12.2.3.1
9.2.2	Peptidbindung		12.2.3	12.2.2.7	12.2.3.1
9.2.3	Strukturen		12.2.3, 12.7.2.2	12.2.2.7	12.2.3.1
9.2.4	Eigenschaften		12.2.3	12.2.2.7	12.2.3.1
9.2.5	Proteinanalytik		12.2.3	12.2.2.7	12.2.3.1
**10**	**Struktur und Eigenschaften von Kohlenhydraten**		12.2.2.7, 12.6.2.2	12.2.3.1, 12.6.3.1	12.2.3.2, 13.3.15.1
**10.1**	**Monosaccharide**		12.2.2.7	12.2.3.1	
10.1.1	Klassifizierung, Struktur		12.2.3	12.2.2.7	12.2.3.1
10.1.2	Stereochemie		12.2.3	12.2.2.7	12.2.3.1
10.1.3	Reaktionen		12.2.3	12.2.2.7	12.2.3.1
10.1.4	Beispiele		12.2.3	12.2.2.7	12.2.3.1
**10.2**	**Disaccharide**		12.2.2.7	12.2.3.1	
10.2.1	Klassifizierung, Struktur		12.2.3	12.2.2.7	12.2.3.1
10.2.2	Eigenschaften		12.2.3	12.2.2.7	12.2.3.1
10.2.3	Beispiele		12.2.3	12.2.2.7	12.2.3.1
**10.3**	**Oligo- und Polysaccharide**		12.2.2.7	12.2.3.1	
10.3.1	Klassifizierung, Struktur		12.2.3	12.2.2.7	12.2.3.1
10.3.2	Beispiele		12.2.3	12.2.2.7	12.2.3.1
**11**	**Struktur und Eigenschaften von Lipiden**		12.2.2.7, 12.6.2.2	12.2.3.1, 12.6.3.1	12.2.3.4, 13.3.15.3
**11.1**	**Klassifizierung, Struktur und Eigenschaften**		12.2.3	12.2.2.7	12.2.3.1
**11.2**	**Speicherlipide**		12.2.2.7	12.2.3.1	
11.2.1	Klassifizierung, Struktur		12.2.3	12.2.2.7	12.2.3.1
11.2.2	Eigenschaften, Reaktionen		12.2.3	12.2.2.7	12.2.3.1
11.2.3	Beispiele		12.2.3	12.2.2.7	12.2.3.1
**11.3**	**Membranlipide**		12.2.2.7	12.2.3.1	
11.3.1	Klassifizierung, Struktur		12.2.3	12.2.2.7	12.2.3.1
11.3.2	Eigenschaften, Reaktionen		12.2.3	12.2.2.7	12.2.3.1
**12**	**Struktur und Eigenschaften von Nucleotiden und Derivaten, Nucleinsäuren**		12.2.2.7, 12.6.2.5	12.2.3.1, 13.3.15.4	12.2.3.5, 12.6.1.9
**12.1**	**Nucleoside, Nucleotide und Nucleotidderivate**				
12.1.1	Klassifizierung, Struktur		12.2.3		
12.1.2	Reaktionen		12.2.3		
12.1.3	Beispiele		12.2.3		
**12.2**	**Nucleinsäuren**				
12.2.1	Klassifizierung, Struktur		12.2.3		
12.2.2	Reaktionen		12.2.3		
**13.**	**Vitamine und Vitaminderivate**		12.2.3.6		

**13.1**	**Definition und Klassifikation**		12.2.3		
**13.2**	**Strukturprinzipien**		12.2.3		
**13.3**	**Herkunft, Stabilität**		12.2.3		
**13.4**	**Funktion**		12.2.3		
**13.5**	**Stoffwechsel**		12.2.3		
**14**	**Energetik und Kinetik biochemischer Reaktionen**				
**14.1**	**Fließgleichgewicht**	Struktur und Funktionsweise von Enzymen	12.2.2	12.2.4.1	
**14.2**	**Gekoppelte Reaktionen**		12.2.2	12.2.4.1	
**14.3**	**„Energiereiche" Verbindungen**		12.2.2	12.2.4.1	
**14.4**	**Biokatalyse**		12.2.4	12.2.4.1	
**14.5**	**Cofaktoren**	Struktur und Funktionsweise von Enzymen	12.2.3	12.2.4.1	12.2.3.6, 12.6.1.7
**14.6**	**Enzymkinetik**		12.2.4	12.2.4.1	
**14.7**	**Hemmung von Enzymen**		12.2.4	12.2.4.2	
**14.8**	**Abhängigkeit der Enzymaktivität**		12.2.4	12.2.4.1	
**15**	**Prinzipien der Enzymregulation**		12.2.4.1	12.2.4.3	
**15.1**	**Abhängigkeit der Enzymaktivität von der Substratkonzentration**		12.2.4		
**15.2**	**Negative Rückkopplung**		12.2.4		
**15.3**	**Allosterische Regulation**		12.2.4		
**15.4**	**Enzymgesteuerte chemische Modifikation von Enzymen**		12.2.4		
**15.5**	**Veränderung der Enzymkonzentration**		12.2.4		
**15.6**	**Limitierte Proteolyse**		12.2.4		
**15.7**	**Protein-Protein-Interaktion**		12.2.4		
**15.8**	**Kontrollierte räumliche Trennung von Enzym und Substrat**		12.2.4		
**16.**	**Kataboler Stoffwechsel und Energiegewinnung**		12.6.1, 13.3.15.4	12.6.1.7	13.3.15.1, 13.3.15.3
**16.1**	**Kohlenhydratabbau**		12.6.1	12.6.1.1	12.6.2.1.
**16.2**	**Triacylglycerol-(Triglycerid-) und Fettsäureabbau**		12.6.1	12.6.1.2	
**16.3**	**Ketonkörpersynthese und -abbau**		12.6.1	12.6.1.3	
**16.4**	**Aminosäureabbau**		12.6.1	12.6.1.4	12.15.3.4
16.4.1	Prinzipien des Aminosäurestoffwechsels		12.6.1	12.6.2.4	
16.4.2	Stoffwechselprodukte von Aminosäurer		12.6.1	12.6.3.1	12.6.3.2
16.4.3	organbezogener Aminosäurestoffwechse		12.6.1		
**16.5**	**Ethanolabbau**		12.6.1	12.6.1.5	
**16.6**	**Pyruvat-Dehydrogenase, Citrat-Zyklus**		12.6.1	12.6.1.6	
**16.7**	**Atmungskette und oxidative Phosphorylierung**		12.6.1		
16.7.1	Aufbau		12.6.1		
16.7.2	Arbeitsweise		12.6.1		
16.7.3	Reaktive Sauerstoffspezies (ROS)		12.6.1		
**17**	**Anaboler Stoffwechsel und Aufbau von Energiespeichern**		13.3.15.1	13.3.15.3	13.3.15.4
**17.1**	**Kohlenhydrate**		12.6.2.2		
17.1.1	Verwertung von Glucose		12.6.1		
17.1.2	Gluconeogenese		12.6.1		
17.1.3	Glykogensynthese		12.6.1	12.6.3.1	12.6.3.2
**17.2**	**Lipide**		12.6.2.3		
17.2.1	Stoffwechsel von Lipoproteinen und Fettsäuren		12.6.1, 12.15.3.2	12.6.3.1	12.6.3.2
17.2.2	Fettsäuresynthese		12.6.1	12.6.2.1	
17.2.3	Triacylglycerolsynthese (Triglyceridsynthese)		12.6.1	12.6.3.1	12.6.3.2
**18**	**Regulation des Energiestoffwechsels**	Regelprozesse und Regelprinzipien	12.2.1.1	13.3.15.1	13.3.15.3, 13.3.15.4
**18.1**	**Begriffe und Grundlagen**		12.6.1		
**18.2**	**Bildung von Energiespeichern**		12.6.1		12.6.3.2
**18.3**	**Speicherverwertung**		12.6.1	12.10.3.6	
**18.4**	**Regulation des Glucoseabbaus und des Citrat-Zyklus**		12.6.1		
**19**	**Speicherung, Übertragung und Expression genetischer Information**		13.2.6.1	13.2.6.4	
**19.1**	**Nucleotide**		12.6.2.5		
19.1.1	Synthese		12.2.3	12.6.2	
19.1.2	Funktion		12.2.3		
19.1.3	Abbau		12.2.3	12.6.1	12.6.1.9
**19.2**	**Nucleinsäuren**				
19.2.1	Grundbegriffe		12.2.5	12.2.3	
19.2.2	DNA-Replikation		12.2.5	12.2.5.2	12.2.5.3
19.2.3	DNA-Schädigung und Reparatur		12.2.5	12.2.5.4	
19.2.4	Transkription		12.2.5	12.3.1.4	12.2.5.7
19.2.5	Regulation der Transkription	Regulation von Enzymen durch allosterische Regulatoren	12.2.5, 12.5.3.3	12.2.4.3, 12.3.1.4	12.3.1.4, 12.2.5.7
19.2.6	posttranskriptionelle Veränderungen		12.2.5	12.3.1.4	12.2.5.7
19.2.7	Translation		12.2.5	12.2.5.7	
19.2.8	Hemmstoffe der Translation	Regulation von Enzymen durch allosterische	12.2.5	12.2.4.3	12.2.5.7
19.2.9	Regulation der Translation		12.2.5	12.2.4.3	12.2.5.7
19.2.10	Viren		12.2.5	12.2.5.2	
19.2.11	Gentechnik		12.2.5		
19.2.12	Analyse von Nucleinsäuren		12.2.5	12.2.5.1	
**19.3**	**Co- und posttranslationale Faltung und Modifikation von Proteinen, Proteomen**		12.2.3.3		
19.3.1	Proteinfaltung		12.2.3		
19.3.2	Proteinsortierung	Mechanismen, durch die Proteine und RNA transportiert werden	12.2.3	12.3.1.4	
19.3.3	limitierte Proteolyse		12.2.3		

19.3.4	Proteinglykosylierung		12.2.3	12.7.2.2	
19.3.5	nichtenzymatische Glykierung		12.2.3		
19.3.6	Verankerung von Proteinen an Membranen		12.2.3		
19.3.7	reversible Modifikationen von Proteinen		12.2.3		
19.3.8	Proteome		12.2.3		
**19.4**	**Proteolyse**		12.7.2.2		
19.4.1	Proteasen		12.2.4		
19.4.2	lysosomale Proteolyse		12.2.4		
19.4.3.	zytosolische Proteolyse		12.2.4		
**19.5**	**Tumorbiochemie**				
19.5.1	Kanzerogenese	Mechanismen der Mutationsentstehung und DNA-Reparatur	13.2.4	12.2.5.4	12.3.3.1
19.5.2	Therapie		16.4.9	16.5.62	
19.5.3	Apoptose		12.3.5	12.3.5.1	12.3.5.2, 12.7.2.2
**20**	**Molekulare Zellbiologie**				
**20.1**	**Eukaryontische Zellen**		12.3.1	12.3.1.1	
20.1.1	Aufbau		12.3.1	12.3.1.1	
**20.2.**	**Membranen (s. a. GK Biol. 1.2)**	stoffliche Eigenschaften der Materie unkl. Phasen, -gleichgewichte und Gleichgewichte; Aufbau und Funktion von Proteinen; Aufbau unf Funktion von Fettsäuren und Lipiden	12.9.1.4	12.2.3.3, 12.3.1.1	12.2.3.4, 12.3.1.2
20.2.1	Membrankomponenten		12.3.1	12.3.1.1	
20.2.2	Bildung und Abbau von Membranen		12.3.1	12.3.1.1	
20.2.3	Funktion		12.3.1	12.3.2.1	12.12.2.2
**20.3**	**Zellkern (s. a. GK Biol. 1.3)**		12.3.1	12.3.1.1	
20.3.1	Chromatin	Zelle, Chromosom	12.3, 12.3.1	12.2.5, 12.3.1.1	12.2.5.1, 12.2.5.2
20.3.2	Kernhülle	Aufbau der Zelle	12.3	12.2.5, 12.3.1.1	
20.3.3	Funktionen	"	12.3, 12.3.1	12.2.5, 12.3.1.1	12.2.5.1
**20.4**	**Mitochondrien (s. a. GK Biol. 1.12)**		12.3.1	12.3.1.1	
20.4.1	Entstehung, Aufbau	Aufbau der Zelle	12.3.1	12.2.5.5	12.3.1.1
20.4.2	Funktionen		12.3.1	12.3.1.1	
**20.5**	**Lysosomen (s. a. GK Biol. 1.8)**		12.3.1	12.3.1.1	
20.5.1	Entstehung, Aufbau	Aufbau der Zelle	12.3.1	12.3.1.1	12.3.1.4
20.5.2	Funktionen		12.3.1	12.3.1.1	
**20.6**	**Peroxisomen (s. a. GK Biol.1.11)**		12.3.1	12.3.1.1	
**20.7**	**Endoplasmatisches Retikulum (ER) (s. a. GK Biol. 1.6)**		12.3.1	12.3.1.1	
20.7.1	glattes ER	Aufbau der Zelle	12.3.1	12.3.1.1	
20.7.2	raues ER		12.3.1	12.3.1.1	12.3.1.4
**20.8**	**Golgi-Apparat (s. a. GK Biol. 1.7)**		12.3.1	12.3.1.1	
20.8.1	Aufbau	Aufbau der Zelle	12.3.1	12.3.1.1	
20.8.2	Funktionen		12.3.1	12.3.1.1	
**20.9**	**Zytoskelett (s. a. GK Biol. 1.13)**		12.3.1	12.3.1.1	12.3.1.3
20.9.1	Aufbau und Funktion	Aufbau der Zelle	12.3.1	12.3.1.1	
**20.10**	**Extrazelluläre Matrix**		12.3.1	12.3.1.1	12.3.1.5
20.10.1	Strukturprinzip, Vorkommen	Aufbau der Zelle	12.3.1		
20.10.2	Synthese, Abbau		12.3.1	12.4.2.1	
20.10.3	Funktion		12.3.1		
**20.11**	**Zellzyklus (s. a. GK Biol. 1.14)**	Ablauf von Mitose und Meisose	12.2.5.6		
20.11.1	Ablauf und Regulation		12.3.3	12.7.2.2	
**21**	**Säure-Basen-Haushalt, Wasser- und Elektrolythaushalt, Spurenelemente und Schwefel**				
**21.1**	**Säure-Basen-Haushalt (s. a. GK Physiol. 5.10)**	Mechanismen der Regulation des zellulären pH-Wertes	12.3.2.4		
21.1.1	Protonenbilanz	Reaktionstypen bei Säuren, Basen	12.2.2.6		
21.1.2	pH-Homöostase	pH	12.3.2	12.14.3.5	
**21.2**	**Wasser- und Elektrolythaushalt**	Funktion von Elektrolyten und Spurenelementen	12.2.3.7	12.16.2	12.16.3.3
21.2.1	Stoffwechsel des Wassers		12.16.2	12.16.2.2	
21.2.2	Biochemie der Elektrolyte		12.16.2, 12.16.2.4	12.5.2.3, 12.16.2.5	12.16.2.3
**21.3**	**Spurenelemente**		12.2.3.7		
21.3.1	Eisen		12.2.3		
21.3.2	Kupfer		12.2.3		
21.3.3	Zink		12.2.3		
21.3.4	Iod		12.2.3		
21.3.5	Selen		12.2.3		
**21.4**	**Schwefel**	Funktion von Elektrolyten und Spurenelementen	12.2.3.7		
**22**	**Bewegung**		12.3.1.3		
**22.1**	**Kontraktile Systeme (s. a. GK Physiol. Kap. 13)**				
22.1.1	Actomyosin- System in Muskelzellen	Aufbau Muskelgewebe	12.4.3	12.3.1.3	12.4.3.2
**22.2**	**Motile Systeme**				
22.2.1	mikrotubuläres System	Organellen und Komponenten des	12.3.1.3		
22.2.2	Actin in Nichtmuskelzellen		12.3.1.3		
**23**	**Hormone, hormonähnliche Signalstoffe und Cytokine (s. a. GK Physiol. Kap. 10)**		12.5.		

**23.1**	**Grundlagen**	Regelprozesse und Regelprinzipien	12.2.1.1	12.5.1	
23.1.1	Grundlagen der hormonellen Kommunikation		12.5.1	12.5.2.2	
23.1.2	Hormone, Cytokine und hormonähnliche Substanzen		12.5.1	12.5.1.4	12.5.1.5
23.1.3	Hormon- und Cytokinrezeptoren		12.5.1, 12.5.2, 12.5.3.1	12.5.1.4, 12.5.2.2, 12.5.3.3	12.5.1.5, 12.5.2.1, 12.5.3.2
23.1.4	Signaltransduktion		12.5.1, 12.5.3, 12.5.2.1	12.5.1.4, 12.5.3.1, 12.5.1.5	12.18.6.2, 12.18.9.1, 12.18.7.2
23.1.5	Neurohormone		12.5.1, 12.5.1.6	12.5.1.4	12.5.1.5
**23.2**	**Biochemie von Hormonen (s. a. GK Physiol. 10.2)**		12.5.1.8		
23.2.1	Insulin	Hormon	12.5.1		
23.2.2	Glucagon		12.5.1		
23.2.3	Adrenalin und Noradrenalin		12.5.1	12.11.6.5	
23.2.4	Glucocorticoide		12.5.1	12.5.1.3	
23.2.5	Somatotropin (STH, GH)		12.5.1	12.5.1.3	
23.2.6	Schilddrüsenhormone		12.5.1	12.5.1.3	12.11.6.5
23.2.7	Sexualhormone		12.5.1, 12.17.3.1	12.5.1.3	12.17.2.4
23.2.8	Prolactin		12.5.1	12.17.3.1	
23.2.9	Oxytocin		12.5.1		
23.2.10	gastrointestinale Hormone		12.5.1	12.15.2.3	12.15.4.2
23.2.11	orexigen und anorexigen wirkende Hormone und Mediatoren		12.5.1	12.6.4.3	12.15.4.2
23.2.12	Aldosteron		12.5.1	12.11.6.5	
23.2.13	Renin-Angiotensin-System		12.5.1	12.11.6.5	
23.2.14	atriales natriuretisches Hormon (Atriopeptin, ANF)		12.5.1	12.11.6.5	
23.2.15	Adiuretin (Vasopressin)		12.5.1		
23.2.16	Parathormon		12.5.1	12.16.2.5	
23.2.17	Calcitonin		12.5.1		
23.2.18	Calciferole		12.5.1	12.16.2.5	
23.2.19	Histamin		12.5.1	12.13.1.2	
23.2.20	Serotonin		12.5.1		
23.2.21	Kinine (Bradykinin, Kallidin)		12.5.1	12.13.1.2	
23.2.22	Eicosanoide (Prostaglandine, Leukotriene, Thromboxane)		12.5.1	12.12.2.3	12.13.1.2
**23.3**	**Biochemie der Cytokine**		12.13.1.2		
23.3.1	proinflammatorische Cytokine	Cytokin	12.5.1		
23.3.2	Chemokine		12.5.1		
23.3.3	Interleukine		12.5.1		
23.3.4	Wachstumsfaktoren		12.5.1		
23.3.5	Cytokinmangel, -überschuss		12.5.1		
**24**	**Immunsystem**		12.13.1.1		
**24.1**	**Zellen des Immunsystems**		12.13.1, 12.13.4.1	12.13.1	12.13.3.2
**24.2**	**Begriffe**		12.13.1	12.13.4.4	
**24.3**	**Immunglobuline**		12.13.4	12.13.4.2	
**24.4**	**Histokompatibilitätsantigene, Antigenpräsentation**		12.13.4	12.13.4.4	
**24.5**	**T-Zellrezeptor, T-Zell-Antigenerkennung**		12.13.4	12.13.4.2	
**24.6**	**Unspezifische Immunantwort**		12.13.3	12.13.3.1	
**24.7**	**Spezifische Immunantwort**		12.13.4	12.13.4.1	12.13.4.3
**24.8**	**Immunologische Abwehrmechanismen**		12.13.3	12.13.4	
**25**	**Blut**		12.12.1		
**25.1**	**Erythropoiese und Erythrozyten (s. a. GK Physiol. 2.2)**				
25.1.1	Sauerstoffaufnahme und -versorgung		12.12.2	12.11.6.11	12.12.2.1
25.1.2	CO2-Transport		12.12.2	12.12.2.1	
25.1.3	Hämoglobin		12.12.1	12.12.2.1	
25.1.4	Erythropoiese und Erythrozytenabbau		12.12.1, 12.12.1.4	12.11.6.11	12.12.1.1
25.1.5	Stoffwechsel		12.12.2	12.11.6.11	12.12.2.4
**25.2**	**Granulozyten, Makrophagen**		12.12.1, 12.13.3.2	12.12.1.1	12.13.1
**25.3**	**Lymphozyten**		12.12.1	12.12.1.1	
**25.4**	**Blutstillung, Blutgerinnung und Fibrinolyse (s.a. GK Physiol. 2.4)**		12.12.2		
25.4.1	Thrombozyten		12.12.1		
25.4.2	Blutgerinnung		12.12.2		
25.4.3	Fibrinolyse		12.12.2		
**25.5**	**Blutplasma (s.a. GK Physiol. 2.3)**		12.12.1, 12.15.3.2	12.12.1.3	12.12.2.2
**26**	**Leber**		12.15.3		
**26.1**	**Energiestoffwechsel**		12.15.3	12.6.4	12.6.4.1
**26.2**	**Serviceleistungen**		12.15.3, 12.15.3.3	12.6.4.1, 12.15.3.4	12.6.4.3
**26.3**	**Cholesterol (Cholesterin)**		12.15.3	12.15.2.6	12.15.3.2
**26.4**	**Gallenflüssigkeit und Gallensäuren (s. a. GK Physiol. 7.3.5)**		12.15.3	12.15.2.6	12.15.3.5
**26.5**	**Biotransformation**		12.15.3.5	12.15.3.6	
26.5.1	Prinzip und Bedeutung		12.15.3		
26.5.2	Phase 1		12.15.3		
26.5.3	Phase 2		12.15.3		
26.5.4	Phase 3		12.15.3		
26.5.5	Induktion des Biotransformationssystems		12.15.3		
**26.6**	**Endokrine Funktionen**		12.15.3		
**26.7**	**Leberfunktionsstörungen**		12.15.3		
**27**	**Magen-Darm-Trakt**				
**27.1**	**Grundlagen der Ernährung (s. a. GK Physiol. 7.1.1, 7.1.2 und 8.1.2)**	Aufbau und Funktion von Vitaminen und Cofaktoren; Funktion von Elektrolyten und Spurenelementen	12.2.3.6	12.2.3.7	
27.1.1	Nahrungsbestandteile		12.15.4	12.15.4.1	

27.1.2	Bilanz		12.15.4		
**27.2**	**Verdauung und Resorption(s. a. GK Physiol. 7.3, 7.4 und 7.5)**	Aufbau und Funktion von Vitaminen und Cofaktoren	12.2.3.6, 12.15.2.5	12.2.3.6	12.15.2.2
27.2.1	Verdauungssekrete		12.15.2	12.15.2.5	
27.2.2	Kohlenhydrate		12.15.2	12.15.2.4	12.15.2.5
27.2.3	Proteine		12.15.2	12.15.2.4	12.15.2.5
27.2.4	Lipide		12.15.2	12.15.2.4	12.15.2.5
27.2.5	Vitamine		12.15.2	12.15.2.5	
27.2.6	Hypo- und Hypervitaminosen (s. a. GK Physiol. 7.1.2		12.15.2	12.15.2.5	
27.2.7	Wasser, Elektrolyte und nicht verdaute Nahrungsbestandteile		12.15.2, 12.15.2.5	12.15.4, 12.15.2.7	12.5.2.3
**27.3**	**Endokrine Funktionen**		13.3.10	12.15.2.5	
**28**	**Fettgewebe**		12.6.4	12.6.4.1	12.6.4.3
**28.1**	**Stoffwechselleistungen**		12.6.1	12.6.2	12.6.5.2, 12.6.5.3
**28.2**	**Endokrine Funktionen**		12.6.5.3		
**29**	**Niere**		12.6.5.3	12.6.5.3	
**29.1**	**Stoffwechsel**		12.6.4	12.6.4.1	
**29.2**	**Endokrine Funktionen**		x		
**29.3**	**Grundlagen der Harnbildung (s. a. GK Physiol. 9.2)**		12.16.3	12.16.3.2	
**29.4**	**Ausscheidung von Säuren und Ammoniak**		12.16.2	12.16.2.6	
**30**	**Muskulatur (s. a. GK Physiol. Kap. 13)**				
**30.1**	**Energiestoffwechsel**		12.6.4.1	12.6.4	12.10.3.4
30.1.1	Skelettmuskel		12.10.3	12.6.4.3	
30.1.2	Herzmuskel		x		
30.1.3	glatte Muskulatur		x		
**30.2**	**Kontraktion, Relaxation**		12.4.3	12.10.3	12.4.3.2
**30.3**	**Endokrine Funktion**		12.10.3		
**31**	**Aufbau des Stützgewebes**				
**31.1**	**Extrazelluläre Matrix**		12.3.1	12.4.2	
**31.2**	**Knorpelgewebe (s. a. GK Anat.2.5.3)**		12.10.1		
**31.3**	**Knochen, Zahnhartsubstanz (s. a. GK Anat. 2.5.4 und 2.5.5)**		12.10.1	12.4.2	12.4.2.4
**32**	**Nervensystem**				
**32.1**	**Stoffwechsel**		12.18.2, 12.6.4.3	12.6.4	12.6.4.1
**32.2**	**Blut-Hirn-Schranke, Liquor cerebrospinalis (s. a. GK Anat. 9.11.2 und 9.9.4)**		12.18.1	12.9.1.4	
**32.3**	**Myelin**		12.4.4	12.4.4.1	
**32.4**	**Erregungsleitung und –übertragung durch Neurotransmitter (s. a. GK Physiol. 12.3 u. 12.4)**		12.4.4.3	12.5.1.7	
32.4.1	Grundlagen der Erregungsübertragung	Zustandekommen des Membranpotentials	12.5.1, 12.5.2, 12.5.2.3	12.5.2, 12.5.2.1	12.3.2.2, 12.5.2.2
32.4.2	Acetylcholin		12.5.1	12.5.2, 12.5.1.8	12.5.1.6
32.4.3	Catecholamine		12.5.1	12.5.2, 12.5.1.8	12.5.1.6
32.4.4	Glutamat		12.5.1	12.5.2, 12.5.8	12.5.1.6
32.4.5	GABA		12.5.1	12.5.2, 12.5.8	12.5.1.6
32.4.6	Glycin		12.5.1	12.5.2, 12.5.8	12.5.1.6
32.4.7	Serotonin		12.5.1	12.5.2, 12.5.8	12.5.1.6
32.4.8	Stickstoffmonoxid (NO)		12.5.1	12.5.2, 12.5.8	12.5.1.6
32.4.9	Nucleotide und Nucleoside		12.5.1	12.5.2	12.5.1.8
32.4.10	peptiderge Neurotransmitter		12.5.1	12.5.2	12.5.1.8
32.4.11	lipiderge Neurotransmitter		12.5.1	12.5.2	12.5.1.8
**33**	**Sinnesbiochemie (s. a. GK Physiol. 17.2 und 19.3)**				
**33.1**	**Sehen**		12.18.3		
**33.2**	**Riechen**		12.18.5	12.18.5.2	

3.3. Physik für Mediziner

GK Physik		Abgleich mit NKLM		
**1**	**Grundbegriffe des Messens und der quantitativen Beschreibung**			
1.1	Physikalische Größen und Einheiten	12.2.1	12.10.1	
1.2	Mengengrößen, bezogene Größen	x		
1.3	Messen und Unsicherheiten beim Messen	14a.2.2	14a.2.3	
1.4	Zusammenhänge zwischen physikalischen Größen	12.2.1		
**2**	**Mechanik**	12.2.1.2		
2.1	Bewegungen	12.2.1.2		
2.2	Impuls, Kraft	12.2.1.2		
2.3	Drehmoment, Trägheitsmoment, Drehimpuls	12.2.1.2		
2.4	Arbeit, Energie; Leistung	12.2.1.2		
2.5	Verformung fester Körper	12.2.1.2		
2.6	Druck	12.2.1.2		
2.7	Kräfte an Grenzflächen	12.2.1.2	12.2.1.7	
2.8	Strömung von Flüssigkeiten und Gasen	12.2.1.2	12.2.1.8	
**3**	**Struktur der Materie**			
3.1	Aufbau der Atome und Atomkerne	12.2.2		
3.2	Festkörper, Flüssigkeiten, Gase	12.2.2		
**4**	**Wärmelehre**	12.2.1.3		
4.1	Temperatur	12.2.1.3		
4.2	Wärme, Wärmekapazität	12.2.1.3		
4.3	Gaszustand	12.2.1.3	12.2.1.7	
4.4	Änderung des Aggregatzustands	12.2.1.3		
4.5	Wärmetransport, Transportphänomene	12.2.1.3		
4.6	Stoffgemische	12.2.1.3		
**5**	**Elektrizitätslehre**	12.2.1.5		
5.1	Elektrische Stromstärke, elektrische Ladung	12.2.1.5		
5.2	Elektrische Feldstärke	12.2.1.5		
5.3	Elektrisches Potential, elektrische Spannung	12.2.1.5		
5.4	Elektrischer Widerstand	12.2.1.5		
5.5	Elektrischer Stromkreis	12.2.1.5		
5.6	Elektrische Kapazität	12.2.1.5		
5.7	Elektrizitätsleitung	12.2.1.5		
5.8	Elektrische Spannungen an Grenzflächen, Diffusionsspannungen	12.2.1.5		
5.9	Magnetische Größen, elektromagnetische Induktion	12.2.1.5		
5.10	Wechselspannung, Wechselstrom	12.2.1.5		
**6**	**Schwingungen und Wellen**	12.2.1.4		
6.1	Schwingungen	12.2.1.4		
6.2	Wellen	12.2.1.4		
6.3	Schallwellen	12.2.1.4		
6.4	Elektromagnetische Wellen	12.2.1.4		
**7**	**Optik**	12.2.1.6		
7.1	Licht	12.2.1.6		
7.2	Geometrische Optik	12.2.1.6		
7.3	Wellenoptik	12.2.1.6		
7.4	Optische Instrumente	12.2.1.6		
**8**	**Ionisierende Strahlung**	12.2.1.9		
8.1	Röntgenstrahlung	12.2.1.9		
8.2	Radioaktivität	12.2.1.9		
8.3	Nachweis ionisierender Strahlen	12.2.1.9		
8.4	Strahlenwirkungen	12.2.1.9		

3.4. Anatomie

GK Anatomie		Abgleich mit NKLM		
**1**	**Allgemeine Embryologie**			
**1.1**	**Grundlagen der Reproduktion**	12.3.4, 12.17.2	12.7	12.7.1
1.1.1	Keimzellen	12.7.1	12.7.1.1	12.17.2.1
1.1.2	Oogenese und weibliches Genitalsystem	12.7.1	12.17.2.1	12.17.2.4
1.1.3	Spermatogenese und männliches Genitalsystem	12.7.1	12.17.2.1	
1.1.4	Verlauf von Schwangerschaftund Geburt	12.7.1	12.17.3	12.17.3
**1.2**	**Grundlagen der Embryologie**	12.3.5, 12.3.4.2, 12.7.1.3, 12.7.1.5	12.3.3.4, 12.3.5.1, 12.7	12.3.4, 12.7.1, 12.7.1.4
1.2.1	Grundbegriffe der Embryonalentwicklung	12.3.3, 12.3.3.3, 12.7.1.3	12.3.4, 12.3.3.4	12.7.1, 12.3.4.1, 12.3.5
1.2.2	Molekularbiologie der Entwicklung	12.3.3, 12.3.5, 12.7.1.3	12.3.4, 12.3.3.4	12.7.1
**1.3**	**Befruchtung, Furchung und Implantation**	12.3.3.4, 12.3.5.3, 12.7.1.6	12.3.5, 12.7.1.3	12.3.5.2
1.3.1	Befruchtung	12.7.1, 12.3.5	12.3.4.1, 12.7.1.3	12.3.3.4
1.3.2	Furchung	12.7.1, 12.3.5	12.3.3.4, 12.3.4.1	12.7.1.3
1.3.3	Blastozyste	12.7.1, 12.3.5	12.3.3.4, 12.7.1.3	
1.3.4	Implantation	12.7.1, 12.3.5	12.3.3.4, 12.7.1.3	
**1.4**	**Plazentation**	12.3.3.4	12.3.5, 12.7.1.3	
1.4.1	Ausbildung des uteroplazentaren Kreislaufs	12.7.1, 12.17.3.2	12.17.3, 12.7.1.3	12.3.3.4
1.4.2	Form, Feinbau und Funktion der reifen Plazenta	12.7.1, 12.17.3.2	12.1.7.3	12.3.3.4
1.4.3	Ablösung der Plazenta	12.7.1, 12.17.3.2	12.1.7.3	12.3.3.4
**1.5**	**Frühentwicklung und Körperbauplan (Embryogenese)**	12.3.5, 12.3.5.2, 12.7.1.4	12.3.3.4, 12.3.5.3, 12.7.1.6	12.3.5.1, 12.7.1.2
1.5.1	Entwicklung der Keimscheibe und des extraembryonalen Gewebes	12.3.3, 12.3.4.1	12.7.1	12.3.4.1, 12.3.3.4
1.5.2	Differenzierung der Keimscheibe	12.3.3	12.7.1	12.3.3.4
1.5.3	Anlage des Nervensystems	12.3.3	12.7.1	12.3.3.4
1.5.4	Ausbildung der Körperform	12.3.3	12.7.1	12.3.3.4
**1.6**	**Entwicklung und Reifung der Organe (Organogenese)**	12.3.3.4, 12.4.1.1	12.3.5.2, 12.7.1.6	12.3.5.3
1.6.1	Stadieneinteilung, Alters-und Längenangaben	12.7.1		
1.6.2	Entwicklung von Embryo und Fetus	12.7.1		
**1.7**	**Zwillinge, Mehrlinge**	x		
**1.8**	**Fehlbildungen**	20.3		
**2**	**Allgemeine Anatomie, Gewebelehre und Histogenese**	12.8.3.1		
**2.1**	**Allgemeine Anatomie**	12.3.4.2	12.7.1.5	
2.1.1	Gestalt	12.3.4.2		
2.1.2	allgemeine Begriffe	12.3.3.3, 12.8.2.4	12.3.4.2	12.8.2.1, 12.8.2.5
2.1.3	postnatale Änderung der Gestalt	12.3.4.2	12.8.2.4	12.8.2.5
**2.2**	**Methoden**	12.7.1.5		
**2.3**	**Epithelgewebe**	12.3.4.2, 12.4.1.2, 12.7.1.5	12.4.1.1, 12.4.1.3, 1.4.1.5	12.4, 12.4.1, 12.1.4.4
2.3.1	Oberflächenepithelien	12.4.1, 12.7.1.5	12.3.4.2	12.4
2.3.2	Drüsenepithelien und Sekretion (s. a. GK Physiol. 1.3.2 und 7.3)	12.4.1, 12.7.1.5	12.3.4.2	12.4
**2.4**	**Allgemeine Anatomie der exokrinen und endokrinen Drüsen**	12.4	12.3.4.2	12.3.4.2
2.4.1	exokrine Drüsen	12.4.1, 12.7.1.5	12.3.4.2	12.4

2.4.2	endokrine Drüsen	12.4.1, 12.4	12.5.1, 12.5.	12.3.4.2, 12.7.1.5
**2.5**	**Binde- und Stützgewebe**	12.3.1.5, 12.4, 12.4.2.4, 12.7.1.5	12.3.4.2, 12.4.2.5, 12.4.26	12.4.2, 12.4.2.1, 12.4.2.2
2.5.1	Bindegewebe	12.4.2, 12.7.1.5	12.3.4.2	12.4
2.5.2	Fettgewebe (s. a. GK Chemie/Bioch. Kap.28)	12.9.1, 12.4, 12.7.1.5	12.6.5, 12.9.1.2	12.4.2, 12.3.4.2
2.5.3	Knorpelgewebe	12.10.1, 12.4	12.7.1.5, 12.10.3	12.3.4.2, 12.10.2.2
2.5.4	Knochengewebe	12.4, 12.7.1.5	12.10.1, 12.10.2.2	12.4.2.4, 12.3.4.2
2.5.5	Zahnhartsubstanzen	12.4.2, 12.7.1.5	12.3.4.2	12.4
**2.6**	**Muskelgewebe (s. a. Physiol. Kap. 13)**	12.3.4.2, 12.4.3.1	12.4.3, 12.4.3.2	12.4, 12.7.1.5
2.6.1	Skelettmuskulatur	12.4.3, 12.3.4.2	12.10.1, 12.4	12.10.3, 12.7.1.5
2.6.2	Herzmuskulatur(s. a. 7.5)	12.4.3, 12.7.1.5	12.3.4.2	12.4
2.6.3	glatte Muskulatur	12.4.3, 12.7.1.5	12.3.4.2	12.4
**2.7**	**Allgemeine Anatomie des Bewegungsapparates**	12.3.4.2, 12.7.1.5, 12.10.2.2	12.4, 12.4.3, 12.4.3.2	12.4.2.2, 12.10.1
2.7.1	Knochen	12.10.1, 12.7.1.5	12.3.4.2	12.4, 12.10.1.1
2.7.2	Knochenverbindungen	12.10.1, 12.4	12.2.1.2, 12.10.2.3	12.3.4.2, 12.7.1.5
2.7.3	Skelettmuskeln	12.10.1, 12.7.1.5	12.3.4.2, 12.10.3.1	12.4, 12.10.1.1
2.7.4	Zusatzeinrichtungen der Muskeln	12.10.1, 12.7.1.5	12.3.4.2, 12.10.1.2, 12.10.1.3	12.4, 12.4.3.4, 12.10.1.1
**2.8**	**Nervengewebe**	12.3.4.2, 12.4, 12.7.1.5	12.4.4, 12.4.4.1	12.5.1, 12.4.4.2, 12.4.4.3
2.8.1	Neurone	12.4.4	12.4	12.5.
2.8.2	Gliazellen	12.4.4	12.4	
2.8.3	Nervenfaser	12.4.4	12.4	
**2.9**	**Allgemeine Anatomie des Nervensystems**	12.4	12.4.4, 12.4.4.3	12.4.4.1, 12.4.4.2
2.9.1	übergeordnete Gliederungen und allgemeine Begriffe	12.18.2, 12.18.13	12.18.2.1	12.18.2.2
2.9.2	periphere Organisation und Projektion	12.18.2, 12.18.9.3	12.18.2.1	12.18.2.8, 12.18.2.9
2.9.3	Gliederung des peripheren Nervensystems	12.18.2	12.4.4.1, 12.18.2.1	12.4.4.2
2.9.4	mikroskopische Anatomie des peripheren Nervensystems	12.18.2		
**2.10**	**Allgemeine Anatomie des Herz-Kreislaufsystems und Lymphgefäßsystems**	12.8.1.1	12.11.1	12.13.1
2.10.1	Gliederung	12.11.1, 12.11.6.3	12.11.1.2, 12.14.2.9	12.11.1.3
2.10.2	Blutgefäße	12.11.1, 12.11.1.4	12.11.6	12.11.1.1
2.10.3	Lymphgefäßsystem	12.11.1	12.11.1.1	
**2.11**	**Blut und Knochenmark**	12.12.1.2		
2.11.1	Blutzellen	12.12.1, 12.12.1.1	12.12.2	12.12.1
2.11.2	rotes Knochenmark	12.12.1	12.12.1.1	12.13.2.1
**2.12**	**Allgemeine Anatomie des Immunsystems**	12.13		
2.12.1	allgemeine Aspekte (s. a. GK Physiol. 2.5 und GK Chemie/Bioch. Kap. 24)	12.13.1, 12.13.4.1, 12.13.4.4	12.13.3, 12.13.4.2	12.13.3.2, 12.13.4.3
2.12.2	Thymus	12.13.2	12.13.2	12.13.2.1
2.12.3	Milz	12.13.2	12.13.2	12.13.2.2
2.12.4	Lymphknoten	12.13.2	12.13.2	12.13.2.2
2.12.5	mukosaassoziiertes lymphatisches Gewebe (MALT)	12.13.2	12.13.2	12.13.2.2
**3**	**Obere Extremität**	12.8.1.6	12.10.2	
**3.1**	**Grundkenntnisse der Entwicklung**	12.10.1		

**3.2**	**Knochen**	12.8.1, 12.10.2.2	12.10.1	
**3.3**	**Gelenke**	12.10.2.2		
3.3.1	Schultergürtel	12.10.2		
3.3.2	Schultergelenk	12.10.2		
3.3.3	Ellenbogengelenk	12.10.2		
3.3.4	Verbindungen der Unterarmknochen	12.10.2		
3.3.5	Handgelenke	12.10.2		
3.3.6	Fingergelenke	12.10.2		
**3.4**	**Muskeln**	12.8.1.2	12.10.2.4	
3.4.1	Schultergürtelmuskeln	12.10.2		
3.4.2	Schultermuskulatur	12.10.2		
3.4.3	Oberarmmuskulatur	12.10.2		
3.4.4	Unterarmmuskulatur	12.10.2		
3.4.5	Handmuskeln	12.10.2		
**3.5**	**Nerven**	12.8.1.2		
3.5.1	Plexus brachialis	12.1.8.2		
**3.6**	**Arterien**	x		
**3.7**	**Venen**	x		
**3.8**	**Lymphknoten und Lymphgefäße**	x		
**3.9**	**Angewandte und topographische Anatomie**	12.8.1.2	12.8.2.4	12.8.2.5
3.9.1	Oberflächenanatomie	12.8.2		
3.9.2	Regio supraclavicularis	12.8.2		
3.9.3	Regio infraclavicularis, deltoidea und scapularis	12.8.2		
3.9.4	Fossa axillaris (Spatium axillare)	12.8.2		
3.9.5	Schulter	12.8.2		
3.9.6	Oberarm	12.8.2		
3.9.7	Fossa cubitalis	12.8.2		
3.9.8	Unterarm	12.8.2		
3.9.9	Regio carpalis anterior und posterior	12.8.2		
3.9.10	Palma manus	12.8.2		
3.9.11	Dorsum manus	12.8.2		
3.9.12	Finger	12.8.2		
3.9.13	Abgrenzung der sensiblen Innervationsgebiete an Hand und Fingern	x		
3.9.14	anatomische Korrelate bildgebender Verfahren	x		
**4**	**Untere Extremität**	12.8.1.6	12.10.2	
**4.1**	**Grundkenntnisse der Entwicklung**	12.10.1		
**4.2**	**Knochen**	12.8.1, 12.10.2.2	12.10.1	
**4.3**	**Gelenke**	12.10.2.2		
4.3.1	Hüftgelenk	12.10.2		
4.3.2	Kniegelenk	12.10.2		
4.3.3	Verbindung der Unterschenkelknochen	12.10.2		
4.3.4	Sprunggelenke	12.10.2		
4.3.5	weitere Gelenke der Fußwurzel und des Mittelfußes	12.10.2		
4.3.6	Zehengelenke	12.10.2		
**4.4**	**Muskeln**	12.8.1.2	12.10.2.4	
4.4.1	Muskeln der Hüfte	12.10.2		
4.4.2	Oberschenkelmuskeln	12.10.2		
4.4.3	Unterschenkelmuskeln	12.10.2		
4.4.4	Fußmuskeln	12.10.2		
**4.5**	**Nerven**	12.8.1.2		
4.5.1	Plexus lumbosacralis	12.18.2		
**4.6**	**Arterien**	x		
**4.7**	**Venen**	x		
**4.8**	**Lymphknoten und Lymphgefäße**	x		
**4.9**	**Angewandte und topographische Anatomie**	12.8.1.2	12.8.2.4	12.8.2.5
4.9.1	Oberflächenanatomie	12.8.2		
4.9.2	Regio inguinalis	12.8.2		
4.9.3	Trigonum femorale und Fossa iliopectinea	12.8.2		
4.9.4	Regio glutealis	12.8.2		
4.9.5	Hüfte	12.8.2		
4.9.6	Oberschenkel	12.8.2		
4.9.7	Fossa poplitea	12.8.2		
4.9.8	Regio genus	12.8.2		
4.9.9	Unterschenkel	12.8.2		
4.9.10	Regio malleolaris	12.8.2		
4.9.11	Fuß	12.8.2		
4.9.12	Planta pedis	12.8.2		
4.9.13	anatomische Korrelate bildgebender Verfahren	x		
**5**	**Kopf und Hals**	12.18		
**5.1**	**Entwicklung und Wachstum**			

5.1.1	Neurocranium	12.18.1		
5.1.2	Viscerocranium	12.14.1	12.14.1.1	
5.1.3	Hirnnerven, Sinnesorgane	12.18.1		
5.1.4	Gesicht	x		
5.1.5	Hals	x		
**5.2**	**Cranium**	12.10.2.2	12.18.1	12.18.1.1
5.2.1	Schädelkalotte	12.18.1		
5.2.2	Schädelbasis	12.18.1		
5.2.3	Viscerocranium	12.18.1	12.14.1.1	
5.2.4	Kiefergelenk	12.8.1		
**5.3**	**Kopf- und Halsmuskeln, Faszien**	12.8.1.2, 12.10.2	12.8.1.3	
5.3.1	mimische Muskulatur	12.9.1	12.9.1.3	
5.3.2	Kaumuskulatur	x		
5.3.3	Faszien am Kopf und Hals	x		
5.3.4	Zungenbein und Zungenbeinmuskulatur	12.8.1		
5.3.5	Halsmuskulatur	x		
**5.4**	**Kopf- und Halseingeweide**	12.8.1.4, 12.15.2.1+C344	12.14.1	12.15.1
5.4.1	Nasenhöhle	12.14.1.1	12.14.2.2	12.18.5.1
5.4.2	Nasennebenhöhlen	12.14.1.1		
5.4.3	Mundhöhle	12.15.1	12.15.1.1	
5.4.4	Zähne	12.15.1	12.15.1.1	12.15.1.2
5.4.5	Zunge	12.15.1	12.15.1.1	12.18.6.1
5.4.6	Speicheldrüsen (s. a. GK Physiol. 7.3.2)	12.15.1	12.15.1.1	12.15.1.3
5.4.7	Gaumen	12.15.1	12.15.1.1	
5.4.8	Isthmus faucium	12.15.1	12.15.1.1	
5.4.9	Pharynx	12.15.1	12.15.1.1	
5.4.10	Halsteil des Ösophagus	12.15.1		
5.4.11	Larynx	12.8.1, 12.14.2.4	12.14.1.1	12.14.2.3
5.4.12	Halsteil der Trachea	12.14.1	12.14.1.1	
5.4.13	Schilddrüse	12.5.1.1		
5.4.14	Epithelkörperchen	12.5.1.1		
5.4.15	Glomus caroticum	12.18.13.2		
**5.5**	**Hirnnerven**	12.18.2.6		
5.5.1	Sensorische Nerven	12.18.2	12.18.5.1	
5.5.2	Augenmuskelnerven	12.18.2		
5.5.3	N. trigeminus	12.18.2		
5.5.4	N. facialis	12.18.2		
5.5.5	N. glossopharyngeus	12.18.2		
5.5.6	N. vagus	12.18.2		
5.5.7	N. accessorius	12.18.2		
5.5.8	N. hypoglossus	12.18.2		
**5.6**	**Zervikale Spinalnerven**			
5.6.1	Rr. dorsales	12.18.2		
5.6.2	Rr. ventrales	12.18.2		
**5.7**	**Vegetative Innervation am Kopf und Hals**	12.18.2.7		
5.7.1	Pars sympathica	12.18.2		
5.7.2	Pars parasympathica	12.18.2		
**5.8**	**Arterien und Venen**			
5.8.1	A. subclavia (s.a. 3.6 und 7.6.1)	12.18.1		
5.8.2	A. carotis communis	12.18.1		
5.8.3	A. carotis interna	12.18.1		
5.8.4	A. carotis externa	12.18.1		
5.8.5	V. jugularis interna	12.18.1		
5.8.6	Angulus venosus	12.18.1		
**5.9**	**Lymphknoten und Lymphgefäße**			
5.9.1	Lymphknoten an der Kopf-Hals-Grenze	x		
5.9.2	Oberflächliche und tiefe Halslymph- knoten	x		
5.9.3	Lymphbahnen	x		
**5.10**	**Angewandte und topographische Anatomie**	12.5.1.1, 12.8.2.4	12.8.1.2, 12.8.2.5	12.8.1.3
5.10.1	Oberflächenanatomie von Kopf und Hals	12.8.1		
5.10.2	Kopfregionen	12.8.1		
5.10.3	oberflächliche Gesichtsregionen	12.8.1		12.18.3.1
5.10.4	tiefe Gesichtsregionen	12.8.1		12.18.3.1
5.10.5	Spatium peripharyngeum	12.8.1		
5.10.6	Mundboden	12.8.1		
5.10.7	anatomische Korrelate bildgebender Verfahren	12.8.1		
5.10.8	Halsregionen	12.8.1		
**6**	**Leibeswand**	12.8.1.2, 12.10.2	12.8.1.5	
**6.1**	**Rücken**	12.10.2.4		
6.1.1	Entwicklung der Wirbelsäule	12.8.2	12.10.2.4	
6.1.2	Skelettelemente der Wirbelsäule	12.10.2	12.10.2.4	

6.1.3	Verbindungen der Wirbel	12.10.2	12.10.2.4	
6.1.4	Wirbelsäule als Ganzes	12.10.2	12.10.2.4	
6.1.5	Autochthone Rückenmuskulatur	12.8.1	12.10.2.4	
6.1.6	Nerven und Gefäße	12.8.1	12.10.2.4	
6.1.7	angewandte und topographische Anatomie	12.8.1	12.10.2.4	12.18.1.3
**6.2**	**Brustwand**	12.10.2.4		
6.2.1	Grundzüge der Entwicklung des Thorax	12.10.2.4		
6.2.2	Skelettelemente und Verbindungen	12.10.2	12.10.2.4	
6.2.3	Thorax als Ganzes	12.8.1	12.10.2.4	
6.2.4	Interkostalmuskulatur	12.8.1	12.10.2.4	
6.2.5	Zwerchfell	12.8.1	12.10.2.4	
6.2.6	Nerven und Gefäße	12.8.1	12.10.2.4	
6.2.7	Mamma	12.10.2.4		
**6.3**	**Bauchwand**	12.10.2.4		
6.3.1	Grundzüge der Entwicklung und Nabelbildung	x		
6.3.2	Muskulatur	12.8.1		
6.3.3	Nerven und Gefäße der Bauchwand	12.8.1		
**6.4**	**Becken, Beckenwände**			
6.4.1	Skelettelemente und Verbindungen	12.8.1	12.10.2	
6.4.2	Becken als Ganzes	12.8.1		
6.4.3	innere Beckenmuskulatur	12.8.1		
6.4.4	Beckenbodenmuskulatur	12.8.1	12.15.1.6	12.17.1.3
6.4.5	Nerven und Gefäße	12.8.1		
**7**	**Brusteingeweide**	12.8.1.7	12.8.3.1	
**7.1**	**Entwicklung von Pleurahöhlen, Herz und Lunge**	12.8.2.2		
7.1.1	Pleurahöhlen und Zwerchfell	12.14.1	12.3.3	
7.1.2	Herz	12.3.3		
7.1.3	Schlundbogenarterien	12.7.1		
7.1.4	fetaler Kreislauf	12.11.6		
7.1.5	Trachea und Lunge	12.14.1	12.3.3	
**7.2**	**Atmungsorgane**	12.14	12.14.1	12.14.1.2
7.2.1	Trachea	12.14.1		
7.2.2	Lungen	12.14.1	12.14.2.1	
7.2.3	Pleura	12.14.1		
**7.3**	**Ösophagus**	12.15.1		
**7.4**	**Thymus**	12.13.2		
**7.5**	**Herz**			
7.5.1	Gestalt, Bau, Lage	12.11.2	12.11.2	12.11.2.3
7.5.2	Erregungsbildungs-und –leitungsystem (s. a. GK Physiol. 3.1.2)	12.11.3, 12.11.2.4	12.5.4, 12.11.3	12.5.4.1
7.5.3	Gefäße	12.11.4	12.11.2.2	
7.5.4	Nerven	12.11.5		
7.5.5	Herzbeutel	12.11.2	12.11.2.1	
**7.6**	**Arterien, Venen und Lymphgefäße des Thorax**			
7.6.1	Aorta im Thorax	12.11.4		
7.6.2	V. cava superior und inferior	12.11.4		
7.6.3	Pulmonal- und Bronchialgefäße	12.11.4		
7.6.4	Lymphknoten und Lymphgefäße	12.11.4		
**7.7**	**Nerven**	12.18.2.7		
**7.8**	**Angewandte und topographische Anatomie**	12.8.2.4	12.8.2.5	
7.8.1	Oberflächenanatomie	12.8.1		
7.8.2	Projektion der Thoraxorgane auf die Thoraxwand (Skeletotopik)	12.8.1		
7.8.3	Gliederung der Thoraxhöhle und Topographie der Thoraxorgane	12.8.1		
7.8.4	Atemmechanik (s.a. GK Physiol.5.4)	12.14.3	12.14.2.5	
**8**	**Bauch- und Beckeneingeweide**	12.8.1.7	12.8.3.1	
**8.1**	**Entwicklung von Darmtrakt, Harn- und Sexualorganen**	12.8.2.2		
8.1.1	Verdauungsorgane	12.3.3		
8.1.2	Organe im Retroperitonealraum	12.3.3	12.16.1.1	12.17.1
8.1.3	Geschlechtsorgane	12.3.3, 12.17.1.2	12.16.1.1	12.17.1
**8.2**	**Organe des Magen-Darm-Kanals**	12.15, 12.15.1.5	12.15.1, 12.18.9.3	12.15.1.4
8.2.1	Magen	12.15.1		
8.2.2	Dünndarm	12.15.1		
8.2.3	Colon	12.15.1		
8.2.4	Appendix vermiformis	12.15.1		
8.2.5	Rectum	12.15.1		
**8.3**	**Leber, Gallenblase, Pankreas**	12.15.1	12.15.1.4	12.18.9.3
8.3.1	Leber	12.15.1	12.15.3.1	
8.3.2	Gallenblase	12.15.1		
8.3.3	extrahepatische Gallenwege	12.15.1		

8.3.4	Pankreas	12.15.1		
**8.4**	**Milz**	12.15.1	12.13.2.2	
**8.5**	**Endokrine Organe**	12.5.1.1	12.5.1.2	
8.5.1	Nebenniere und Paraganglien	12.5.1		
8.5.2	Inselorgan (endokrines Pankreas)	12.15.1	12.5.1	
8.5.3	Gastroenteropancreatico- endokrines System	12.5.1		
**8.6**	**Harnorgane**	12.16, 12.16.3	12.16.1	12.16.1.1
8.6.1	Niere	12.16.1	12.16.3.2	12.16.3.3
8.6.2	Nierenbecken	12.16.1		
8.6.3	Harnleiter	12.16.1		
8.6.4	Harnblase	12.16.1	12.16.3.4	
8.6.5	weibliche Harnröhre	12.16.1		
**8.7**	**Weibliche Geschlechtsorgane**	12.17	12.17.1	
8.7.1	Ovar	12.17.1	12.5.1.1	12.17.2.1
8.7.2	Tube	12.17.1		
8.7.3	Uterus	12.17.1	12.17.3	
8.7.4	Vagina	12.17.1		
8.7.5	äußere Genitalien	12.17.1		
**8.8**	**Männliche Geschlechtsorgane**	12.17	12.17.1	
8.8.1	Hoden	12.17.1	12.5.1.1	12.17.2.1
8.8.2	Nebenhoden	12.17.1		
8.8.3	Samenstrang	12.17.1		
8.8.4	Glandula vesiculosa (Samenblase)	12.17.1		
8.8.5	Prostata	12.17.1		
8.8.6	äußere Geschlechtsorgane	12.17.1	12.17.2.5	
8.8.7	Ejakulat	12.17.1	12.17.2.2	
**8.9**	**Arterien**			
8.9.1	Pars abdominalis aortae	12.11.1		
8.9.2	Truncus coeliacus	12.11.1		
8.9.3	A. mesenterica superior	12.11.1		
8.9.4	A. mesenterica inferior	12.11.1		
8.9.5	paarige laterale Äste	12.11.1		
8.9.6	Bifurcatio aortae, Aa. iliacae comm.	12.11.1		
8.9.7	A. iliaca externa	12.11.1		
8.9.8	A. iliaca interna	12.11.1		
**8.10**	**Venen**			
8.10.1	V. cava inferior	12.11.1		
8.10.2	Vv. iliacae communis, externa, interna	12.11.1		
8.10.3	V. portae hepatis	12.11.1		
**8.11**	**Lymphgefäße und Lymphknoten**	12.11.1		
**8.12**	**Vegetatives Nervensystem**	12.15.2.7	12.18.2.7	
8.12.1	Pars sympathica	12.18.2		
8.12.2	Pars parasympathica	12.18.2		
**8.13**	**Peritoneum**	12.8.1.8	12.15.1.4	
8.13.1	Peritonealstrukturen	12.8.1		
**8.14**	**Angewandte und topographische Anatomie**	12.5.1.1, 12.8.2.5	12.8.1.8, 12.8.2.4	12.8.2.2
8.14.1	Oberflächenanatomie, Abdomen	12.8.1	12.8.2	12.18.9.3
8.14.2	Organprojektionen auf die Bauchwand, Tastbarkeit	12.8.1	12.8.2	
8.14.3	anatomische Korrelate bildgebender Verfahren	x		
8.14.4	Gliederung der Bauchhöhle, Topographie der Bauchorgane	12.8.1	12.8.2	12.15.1.4
8.14.5	Gliederung des Cavum pelvis, Topographie der Beckenorgane	12.8.1	12.8.2	12.17.1.3
8.14.6	Regio perinealis	12.8.1	12.8.2	
8.14.7	Intraabdominaldruck	12.8.1	12.8.2	12.15.1.6
8.14.8	Schwangerschaft, Geburtsvorgang	12.17.3	12.17.1.3	12.17.3
**9**	**Zentralnervensystem**	12.8.2.4, 12.18.2	12.8.2.5, 12.18.2.2	12.18
**9.1**	**Entwicklung**			
9.1.1	Ausgangsmaterial	12.4.4		
9.1.2	Rückenmark	12.4.4		
9.1.3	Gehirn (Encephalon)	12.4.4		
9.1.4	angeborene Fehlbildungen	12.4.4		
**9.2**	**Rückenmark**			
9.2.1	Gestalt, Gliederung, Lage	12.18.2		
9.2.2	graue Substanz	12.18.2		
9.2.3	weiße Substanz	12.18.2		
9.2.4	funktionelle Anatomie	12.18.2	12.18.11	12.18.11.1
**9.3**	**Rhombencephalon**			
9.3.1	Gestalt, Gliederung, Lage	12.18.2		
9.3.2	innere Gliederung	12.18.2	12.18.13.1	
9.3.3	funktionelle Anatomie (s. a. GK Physiol. 14.3.3 und 4.2.2)	12.18.2	12.15.2.1	12.18.2.6

**9.4**	**Mesencephalon**			
9.4.1	Gestalt, Gliederung, Lage	12.18.2		
9.4.2	innere Gliederung	12.18.2		
9.4.3	funktionelle Anatomie	12.18.2, 12.18.9.2	12.18.2.6	12.18.9
**9.5**	**Cerebellum**			
9.5.1	Gestalt, Gliederung	12.18.2		
9.5.2	innere Gliederung	12.18.2		
9.5.3	Kleinhirnbahnen	12.18.2		
**9.6**	**Diencephalon**	12.5.1.3		
9.6.1	Gestalt, innere und äußere Oberfläche	12.18.2		
9.6.2	Grundlagen der inneren und funktionellen Gliederung	12.18.2		
**9.7**	**Telencephalon**			
9.7.1	Gestalt, Gliederung	12.18.2	12.18.5.1	
9.7.2	Kerne des Telencephalon	12.18.2		
9.7.3	Großhirnrinde	12.18.2	12.18.10	12.18.10.1
9.7.4	Bahnen der Großhirnrinde	12.18.2		
**9.8**	**Systeme**	12.18.2.4		
9.8.1	afferente Systeme, neuronale Gliederung, Umschaltorte	12.18.2, 12.18.5, 12.18.6.2. 12.18.9	12.18.3.7, 12.18.5.2, 12.18.7.2, 12.18.10.2	12.18.4.6, 12.18.6, 12.18.8.2, 12.18.9.2
9.8.2	efferente Systeme, neuronale Gliederung, Umschaltorte	12.18.2	12.18.12	12.18.12.1
9.8.3	limbisches System	12.18.2	12.19.3	12.19.3.2
**9.9**	**Innere Liquorräume**	12.18.1.4		
9.9.1	Seitenventrikel: Gestalt, Gliederung, Lage	12.18.1		
9.9.2	III. Ventrikel: Gestalt, Gliederung, Lage	12.18.1		
9.9.3	IV. Ventrikel: Gestalt, Gliederung, Lage	12.18.1		
9.9.4	Plexus choroideus	12.18.1		
**9.10**	**Hirn- und Rückenmarkshäute, äußere Liquorräume**	12.18.1.2		
9.10.1	Dura mater spinalis et encephali	12.18.1	12.18.1.3	
9.10.2	Arachnoidea mater, Pia mater	12.18.1	12.18.1.3	12.18.1.4
**9.11**	**Gefäßversorgung**	12.5.1.1		
9.11.1	Arterien	12.18.1	12.18.1.6	
9.11.2	Mikrozirkulation	12.18.1		
9.11.3	venöse Abflusswege	12.18.1	12.18.1.5	
**9.12**	**Angewandte und topographische Anatomie**	12.18.1	12.18.1.7	
**10**	**Sehorgan**	12.18	12.18.3	12.18.3.1
**10.1**	**Entwicklung**	x		
**10.2**	**Orbita**	12.8.2.4	12.8.2.5	
10.2.1	Form, Lage	12.18.3		
10.2.2	Peri- und retrobulbärer Bindegewebsraum	12.18.3		
**10.3**	**Bulbus oculi**	12.18.3.2		
10.3.1	Gestalt, Gliederung, Form, mikrosko-pische Anatomie	12.18.3	12.18.3.6	
10.3.2	Tunica fibrosa	12.18.3		
10.3.3	Tunica vasculosa	12.18.3	12.18.3.3	
10.3.4	Tunica interna	12.18.3		
10.3.5	N. opticus	12.18.3		
10.3.6	Bewegungsapparat des Bulbus oculi	12.18.3	12.18.3.3	
**10.4**	**Zusätzliche Einrichtungen**	12.8.2.5		
10.4.1	Augenlid	12.18.3	12.18.3.3	
10.4.2	Bindehaut	12.18.3		
10.4.3	Tränendrüse, Tränenwege	12.18.3	12.18.3.4	
**11**	**Hör- und Gleichgewichtsorgan**	12.18	12.18.4	
**11.1**	**Entwicklung des Hör- und Geleichgewichtsorgans**	x		
**11.2**	**Äußeres Ohr**	12.8.2.4	12.8.2.5	12.18.4.1
11.2.1	Ohrmuschel, äußerer Gehörgang	12.18.4	12.8.2.5	12.18.4.1
**11.3**	**Mittelohr**	12.8.2.5	12.18.4.1	12.18.4.2
11.3.1	Paukenhöhle	12.18.4	12.8.2.5	12.18.4.1
11.3.2	Gehörknöchelchen	12.18.4	12.8.2.5	12.18.4.1
**11.4**	**Innenohr**	12.8.2.4	12.8.2.5	12.18.4.1
11.4.1	Labyrinth	12.18.4	12.18.4.5	
11.4.2	Gleichgewichtsorgan	12.18.8	12.18.8	12.18.8.1
**12**	**Haut und Hautanhangsgebilde**	12.9		
**12.1**	**Haut und Unterhaut**	12.9.1.1		
12.1.1	Epidermis	12.9.1	12.9.1.1	
12.1.2	Dermis	12.9.1	12.9.1.1	
12.1.3	Subcutis	12.9.1	12.9.1.1	
12.1.4	Sinnesfunktion der Haut	12.18.7, 12.18.7	12.9.1, 12.18.7.1	12.9.1.1, 12.18.9.1

**12.2**	**Behaarung**	12.9.1	12.9.1.1	
**12.3**	**Nägel**	12.9.1	12.9.1.1	
**12.4**	**Hautdrüsen**	12.9.1	12.9.1.1	

3.5. Physiologie

GK Physiologie		Synonym	Abgleich mit NKLM		
**1**	**Allgemeine und Zellphysiologie, Zellerregung**		12.3.1.2		
**1.1**	**Stoffmenge und Konzentration (s. a. GK Physik 2.5)**	Prozesse des Verhaltens und Erlebens	12.2.6.2	12.3.1.2	
**1.2**	**Osmose (s. a. GK Physik 4.6)**	Aufbau, Eigenschaft und Funktion von biologischen Membranen	12.3.1.2	12.3.2.3	
**1.3**	**Stofftransport**	aktiver und passiver Substanztransport über Membran	12.3.2.1, 12.5.2, 12.5.2.3	12.4.1.5, 12.5.2.1	12.5.2, 12.5.2.2
1.3.1	in und von Gasen und Flüssigkeiten	Aufbau, Eigenschaft und Funktion von biologischen Membranen	12.3.1.2		
1.3.2	durch Membranen (s. a. GK Ana- tomie 2.3.2 und GK Chemie/Bio-ch. 20.2.3)	membran	12.2.2	12.3.1.2	
1.3.3	intrazellulär	Aufbau, Eigenschaft und Funktion von biologischen Membranen	12.3.1.2		
1.3.4	über Zellverbände (s. a. GK Biol. 1.2.6 und GK Anat. 2.3, 2.6.2 und 2.6.3)	interzellulär	12.5	12.3.1.2	
**1.4**	**Zellorganisation und -beweglichkeit**	Aufbau, Eigenschaft und Funktion von biologischen Membranen	12.3.1.2, 12.3.5.2	12.3.2.4	12.3.5.3
**1.5**	**Elektrische Phänomene an Zellen**	Grundlagen der Elektrizität; Reaktionstypen bei Säuren, Basen, Salzen und Metallkomplexen	12.2.1.5, 12.2.2.6	12.3.1.2	12.3.2.2
1.5.1	Grundphänomene und -funktionen		12.5.4		
1.5.2	Funktion erregbarer Zellen		12.5.4		
**1.6**	**Energetik (s. GK Physik 2.4 und GK Chemie/Bioch. Kap. 14)**		12.5.4	12.3.1.2	
**2**	**Blut und Immunsystem**		12.12.1		
**2.1**	**Blut**		12.12	12.12.1.1	
**2.2**	**Erythrozyten**	Blutzellen	12.12.1	12.12.1.1	
**2.3**	**Blutplasma**		12.12.1	12.12.1.1	12.12.1.3
2.3.1	Transportfunktion		12.12.1		
2.3.2	niedermolekulare Bestandteile		12.12.1		
2.3.3	Plasmaproteine		12.12.1		
**2.4**	**Hämostase und Fibrinolyse**	Gerinnung	12.12.2	12.12.2.3	
2.4.1	Thrombozyten		12.12.2	16.5.59	
2.4.2	Hämostase		12.12.2		
2.4.3	Fibrinolyse		12.12.2		
**2.5**	**Abwehrsystem und zelluläre Identität (Immunologie)**		12.13, 12.13.3.1	12.13.1.1, 12.13.3.2	12.13.1.2, 12.13.4.1
2.5.1	Leukozyten	immunsystem	12.13		
2.5.2	unspezifische Abwehr	angeborenes Immunsystem	12.13	12.13.3	
2.5.3	spezifische Abwehr	Adaptives Immunsystem	12.13	12.13.4	
2.5.4	Entzündungsreaktionen		12.13	13.2.2	
2.5.5	Blutgruppen		12.13	15.2	
**3**	**Herz**				
**3.1**	**Elektrophysiologie des Herzens**	elektro	12.11.3, 12.11.3.2	12.11.3, 12.11.3.3	12.11.3.1, 12.11.3.4
3.1.1	spezielle Elektrophysiologie des Myokards		12.11.3		
3.1.2	Erregungsbildungs- und -leitungssystem (s. a. GK Anat. 7.5.2)		12.11.3	12.5.4	12.5.4.2
3.1.3	elektromechanische Koppelung (s. a. GK Anat. 2.6.2)		12.11.3		
3.1.4	Elektrokardio- graphie (EKG)	Grundlagen der Elektrizitätslehre	12.2.1.5, 12.11.3	14b.3	
**3.2**	**Mechanik des Herzens**		12.11	12.11.4	12.11.4.1, 12.11.4.2
3.2.1	Grundlagen der Muskelkontraktion (s. a. 13.1)		12.11.4		
3.2.2	Herzklappen (s. a. GK Anat. 7.5.1)		12.11.2		
3.2.3	Herzzyklus		12.11.4		
3.2.4	Füllung des Herzens		12.11.2		
**3.3**	**Energiestoffwechsel des Herzens**		12.11.5.3		
3.3.1	Koronardurch- blutung	herzkranzgefäße	12.11.2	12.11.6.5	12.11.6.6
3.3.2	Energieumsatz		x		
**3.4**	**Steuerung der Herztätigkeit**		12.11.3	12.11.5, 12.11.5.1	12.11.5.2
3.4.1	Grundmechanismen		12.11.4	12.11.3	
3.4.2	Herznerven	erregungsleitung	12.11.2	12.18.9.1	
3.4.3	funktionsabhängige Anpassung	hypertrophie	13.2.1, 12.19.10.2	12.18.11.2	12.19.6.7
**4**	**Blutkreislauf**				
**4.1**	**Allgemeine Grundlagen**		12.11.6, 12.11.6.8	12.11.6.1	12.11.6.7
4.1.1		Grundlagen der Strömungslehre	12.2.1.8, 12.11.6		
4.1.2	funktionelle Abschnitte		12.11.6		

4.1.3	Druck		12.11.6	12.11.6.4	
4.1.4	Strömung		12.11.6	12.11.6.5	
4.1.5	Strömungswiderstand		12.11.6	12.11.6.5	
4.1.6	Blutvolumen (s. a. 1.3.4 und 2.1)		12.11.6		
4.1.7	Stoffaustausch (s. a. 1.3.4)		12.11.6	12.11.6.9	12.11.6.10
**4.2**	**Hochdrucksystem**				
4.2.1	arterieller Blutdruck		12.11.6	12.11.1	12.11.6.4
4.2.2	Blutdruckregulation (s. a. GK Anatomie 9.3.3)		12.11.6, 12.11.6.5	12.11.1, 12.18.11.2	12.11.6.4, 12.19.6.7
**4.3**	**Niederdrucksystem**		12.11.6.7	12.11.6.8	
4.3.1	venöser Blutdruck		12.11.1		
4.3.2	intrathorakale Abschnitte		12.11.1		
**4.4**	**Organdurchblutung**				
4.4.1	Grundmechanismen (s. a. 4.1.4)		12.11.1	12.11.6.6	
4.4.2	Lunge		12.14.2	12.11.1	
4.4.3	Gehirn		12.11.6	12.11.6.6	
4.4.4	Niere		12.11.6		
4.4.5	Haut		12.11.6	12.11.6.6	12.19.6.7
4.4.6	Herz		12.11.6		
4.4.7	Skelettmuskel (s. a. 6.2.4)		12.11.6	12.11.6.6	
4.4.8	Splanchnikusgebiet		12.11.6	12.11.6.6	
**4.5**	Fetaler und plazentarer Kreislauf (s. 11.7 und 11.8)	foetal	12.11.6	12.11.6.3	
**5**	**Atmung**				
**5.1**	**Morphologische Grundlagen (s. a. GK Anatomie 7.2)**		12.14.1	12.14.1.2	
**5.2**	**Nicht-respiratorische Lungenfunktion**		12.14.2.2		
**5.3**	**Physikalische Grundlagen (s. a. Kap. 1 sowie GK Physik, 2.7, 4.3 und 4.6)**	Eigenschaften von Gasen	12.2.1.7, 12.14.2.2, 12.14.2		
**5.4**	**Atemmechanik**		12.14.2.5	12.14.2.6	
5.4.1	Lungenvolumina und Statik des Atemapparates		12.14.2		
5.4.2	Dynamik des Atemapparates (s. a. GK Ana- tomie 6.2 und 7.8.4)	lunge	12.14.2		
**5.5**	**Lungenperfusion**	lungenkreislauf	12.14.2	12.11.6.6	12.14.2.9
**5.6**	**Gasaustausch in der Lunge**		12.12.2.1	12.14.2.7	12.14.2.8
5.6.1	O2-Aufnahme, CO2-Abgabe	gasaustausch	12.14.2		
5.6.2	Ventilation		12.14.2		
5.6.3	Diffusion		12.14.2		
5.6.4	Verteilung		12.14.2		
**5.7**	Atemgastransport im Blut (s. a. GK Chemie/Bioch. 25.1.1 bis 25.1.3)		12.14.2	12.12.2.1	12.14.2.8
5.7.1	O2		12.14.2	12.14.3.4	
5.7.2	CO2		12.14.2		
5.7.3	Wechselwirkung zwischen O2- und CO2- Bindung		12.14.2		
**5.8**	**Atmungsregulation**		12.18.13.3		
5.8.1	Atemzentren, Atemreize		12.14.3, 12.18.9.1	12.14.3.1	12.14.3.2
5.8.2	Formen normaler und verän- derter Atmung		12.14.3	12.14.3.2	12.14.3.3
**5.9**	**Atmung unter ungewöhnlichen Bedingungen (s. a. GK Physik 2.7)**		12.14.3.3		
**5.10**	**Säure-Basen-Gleichgewicht und Pufferung (s. a. GK Chemie/Bioch. 3.4 und 21.1)**	Reaktionstypen bei Säuren, Basen, Salzen und Metallkomplexen	12.2.2.6	12.14.3.5	12.16.2.6
5.10.1	Pufferung und H+-Ionen		12.14.3		
5.10.2	Pufferung und CO2-Austausch		12.14.3		
5.10.3	Säure-Basen Haushalt		12.14.3		
5.10.4	Störungen des Säure-Basen- Gleichgewichts		12.14.3		
**6**	**Arbeits- und Leistungsphysiologie**				
**6.1**	**Allgemeine Grundlagen**		19.2.7		
6.1.1	Muskelarbeit (s. a. 8.2 und 13.1.5)		12.10.3		
6.1.2	Kurzzeitbelastung und Ausdauerleistung (s. a. 8.1.3		12.10.3		
**6.2**	**Organbeteiligung**		12.11.6.11		
6.2.1	Blut (s. a. 2.1 und 2.2)		12.11.6		
6.2.2	Lunge (s. a. 5.4.2 und 5.8)		x		
6.2.3	Kreislaufsystem (s. a. 3.4, 4.2.2 und 4.4.7)		12.11.6		
6.2.4	Skelettmuskulatur (s. a. 13.2.2)		12.10.3		
6.2.5	ZNS (s. a. 15.8.4)		12.19.6.7		
**6.3**	**Erfassung von Leistung und Leistungsbeurteilung**				
6.3.1	Spiroergometrie (s. a. 5.4 und 5.6)		15.10.1		
6.3.2	Training (s. a. 13.1.5 und 13.2)		x		
6.3.3	Ermüdung und Erholung (s. a. 13.2.2)		x		
**7**	**Ernährung, Verdauungstrakt, Leber**				
**7.1**	Ernährung (s. a. 8.1 sowie GK Physik 2,4 und GK Chemie/Bioch. 27.1)		12.15.4, 12.15.4.2	12.6.4.3	12.15.4.1
7.1.1	Nahrungsmittel		12.15.4		
7.1.2	inadäquate Ernährung		12.15.4		
7.1.3	Regulation der Nahrungs- aufnahme		12.15.4	12.18.13.4	
**7.2**	**Motorik des Magen-Darm-Trakts**		12.15.2.2	12.15.2.7	12.18.11.2
7.2.1	Grundlagen		12.15.2		
7.2.2	Kauen und Schlucken		12.15.2	12.15.2.1	
7.2.3	Magen		12.15.1		
7.2.4	Erbrechen		20.110		

7.2.5	Dünn- und Dickdarm; Defäkation (s. a. GK Ana- tomie 8.2.5)		12.15.1	12.15.1.6	
**7.3**	**Sekretion**		12.15.2.1	12.15.2.2	12.15.2.6
7.3.1	Grundlagen	Verdauungs-sekrete	12.15.2		
7.3.2	Mund, Rachen, Ösophagus (s. a. GK Ana- tomie 5.4.6)		12.15.2		
7.3.3	Magen (s. a. GK Ana- tomie 8.2.1)		12.15.2		
7.3.4	Pankreas (s. a. GK Ana- tomie 8.3.4)		12.15.2		
7.3.5	Leber und Galle (s. a. GK Che- mie/Bioch. Kap. 26 und GK Anat. 8.3.3)		12.15.2		
7.3.6	Dünn- und Dickdarmsekrete; Stuhl, Darmflora		12.15.2		
**7.4**	Aufschluss der Nahrung (s. a. GK Chemie/Bioch. 27.2)		12.15.2.4	12.15.2.5	
7.4.1	Kohlenhydrate	verdauung	12.15.2		
7.4.2	Proteine		12.15.2		
7.4.3	Lipide		12.15.2		
**7.5**	Absorption (s. a. GK Anatomie 8.2.2 und GK Chemie/Bioch. 27.2.)		12.15.2.5		
7.5.1	Eigenschaften intestinaler Epithelien	verdauung	12.15.2		
7.5.2	Monosaccharide, Aminosäuren, Oligopeptide		12.15.2		
7.5.3	Lipide		12.15.2		
7.5.4	Wasser und Elektrolyte		12.15.2		
7.5.5	sonstige Nahrungs- bestandteile		12.15.2		
**7.6**	Integrative Steuerung der Magen-Darm-Funktion (s. a. 7.2 und 14.3.5)		12.15.2	12.15.2.3	12.18.13.1
**8**	**Energie- und Wärmehaushalt**				
**8.1**	**Energiehaushalt**				
8.1.1	Grundlagen		12.6		
8.1.2	Energiequellen	nahrung	12.6	12.6.1	12.16.1.2
8.1.3	Energieumsatz (s. a. 6.1.2)		12.6		12.16.1.2
**8.2**	**Wärmehaushalt und Temperaturregulation**		12.6.5.3	12.6.5.1	12.18.13.4
8.2.1	Körpertemperatur	thermo	12.6.5		
8.2.2	Wärmebildung		12.6.5		
8.2.3	Wärmeabgabe und -aufnahme		12.6.5	12.6.5.4	
8.2.4	Temperaturregulation		12.6.5		
8.2.5	Akklimatisation		12.6.5		
**9**	**Wasser- und Elektrolythaushalt, Nierenfunktion**				
**9.1**	**Wasser- und Elektrolythaushalt**		12.16.2, 12.16.2.3	12.16.2.1, 12.16.2.4	12.16.2.2, 12.16.2.5
9.1.1	allgemeine Grundlagen		12.16.2	12.2.3	
9.1.2	Flüssigkeitsräume		12.16.2		
9.1.3	Wasser	Elektrolythaushalt	12.16.2	12.2.3	13.3.13
9.1.4	Natrium (s. a. 10.2 und 10.3)	Elektrolythaushalt	12.16.2	12.2.3	12.3.13
9.1.5	Kalium	Elektrolythaushalt	12.16.2	12.2.3	
9.1.6	Calcium	Elektrolythaushalt	12.16.2	12.2.3	
9.1.7	Phosphat	Elektrolythaushalt	12.16.2	12.2.3	
9.1.8	Magnesium		x		
9.1.9	Säure-Basen-Haushalt		12.16.2	12.14.3	
**9.2**	**Niere**		12.16.1.1, 12.16.3.2	12.16.2, 12.16.3.1	12.16.3, 12.16.3.3
9.2.1	Bau und Funktion		12.16	12.16.1	
9.2.2	Durchblutung		12.11.6	12.16.1	12.11.6.6
9.2.3	Filtration		12.16.3	12.16.1	
9.2.4	Transport an renalen Epithelien		12.16.3		
9.2.5	Resorption, Sekretion		12.16.3		
9.2.6	Harnkonzentrierung		12.16.3		
9.2.7	globale Nierenfunktion und Regulation		12.16.3		
9.2.8	Stoffwechsel und Hormonbildung (s. a. GK Che- mie/Bioch. 29.1 und 29.2)		x		
9.2.9	ableitende Harnwege		12.16.1	12.16.3.4	
**10**	**Hormonelle Regulation (s. a. Kap. 7, 9 und 11 sowie GK Chemie/Bioch. Kap. 23)**		12.5.2, 12.5.2.3	12.5.2.1	12.5.2.2
**10.1**	**Allgemeine Hormonlehre**	Regelprozesse und Regelprinzipien	12.2.1.1, 12.5.1.5	12.5.1	12.5.1.4
10.1.1	funktionelle Struktur des Hormonsystems		12.5.1, 12.5.1.5	12.5.1.2	12.5.1.4
10.1.2	Hormoneigenschaften		12.5.1, 12.5.1.5	12.5.1.4	
10.1.3	Signalkette		12.5.1, 12.5.1.5	12.5.2	12.5.1.4
10.1.4	neuroendokrine Signalübertragung		12.5.1, 12.5.1.5	12.5.2, 12.18.13.1	12.5.1.4
**10.2**	**Physiologie einzelner Hormone**		12.5.1.5, 12.5.3.1	12.5.3, 12.5.3.2	12.5.1.4
**10.3**	**Zusammenspiel von Hormonen in Wasser-, Elektrolyt- und Mineralhaushalt, Wachstum, Entwicklung, Stoffwechsel und Energiehaushalt (s. a. Kap. 8 und 9 sowie GK Chemie/Bioch. Kap. 18)**		12.6.4, 12.5.1.5, 12.5.3.1	12.5.1, 12.5.3	12.5.1.4, 12.5.3.2
**11**	**Sexualentwicklung und Reproduktionsphysiologie (s. a. 10.1)**		12.17.3.1	12.17.3.2	
**11.1**	Geschlechtsfestlegung und Pubertät (s. a. GK Biol. 2.4.3 und GK Psych./Soz. 1.4.8)		12.7.1		

**11.2**	Estrogene und Progesteron (s. a. GK Chemie/Bioch. 23.2.7)	zyklus	12.1.7.2	12.17.2.3	12.18.4.1
**11.3**	**Menstruationszyklus**		12.17.2.4		
**11.4**	Androgene (s. a. GK Chemie/Bioch. 23.2.7)		12.1.7.2	12.17.2.2	
**11.5**	Gameten (s. a. GK Anatomie 1.1, 8.7.1 und 8.8.1)		12.1.7.2, 12.17.2.1	12.7.1	12.7.1.1
**11.6**	Kohabitation und Befruchtung (s. a. GK Anat. 1.3 und 8.8.6 sowie GK Psych./Soz. 2.4.6)		12.1.7.2	12.7.1.3	12.17.2.5
**11.7**	Schwangerschaft (s. a. GK Anat. 1.1.4 und 1.4 sowie GK Chemie/Bioch. 23.2.7)		12.1.7.3	12.11.6.3	
**11.8**	**Fetus**		12.1.7.3	12.11.6.3	
**11.9**	Geburt (s. a. GK Anat. 1.1.4 und 18.14.8)		12.1.7.3	12.18.13.1	
**11.10**	**Laktation**		x		
**11.11**	**Alter**		12.20	13.3.7	
**12**	**Funktionsprinzipien des Nervensystems**				
**12.1**	**Ionenkanäle s. 1.3.2**		12.5.2		
**12.2**	**Ruhemembranpotential s. 1.5.1**		12.3.2		
**12.3**	**Signalübertragung in Zellen (s. a. GK Chemie/Bioch. 32.4)**	Grundlagen der Elektrizitätslehre	12.2.1.5, 12.5.2.1, 12.5.4.1	12.5.2, 12.5.2.2	12.5.1, 12.5.2.3
12.3.1	passive elektrische Eigenschaften		12.5.4	12.5.1	
12.3.2	Aktionspotential		12.5.4	12.5.1	
12.3.3	Fortleitung des Aktionspotentials		12.5.4	12.5.1	
12.3.4	intrazellulärer Transport (s. a. 1.3.3)		12.5.4	12.5.1	
**12.4**	**Signalübertragung zwischen Zellen (s. a. GK Chemie/Bioch. 32.4 und GK Anat. 2.8.1)**		12.5.2, 12.5.1.7, 12.5.2.2	12.4.4.3, 12.5.1.8, 12.5.4.2	12.5.1.6, 12.5.2.1, 12.5.2.3
12.4.1	Prinzipien synaptischer Übertragung		12.5.4		
12.4.2	Transmitterfreisetzung		12.5.4	12.5.1	12.5.2
12.4.3	Transmitter		12.5.4, 12.18.2.5	12.5.1	12.5.2
12.4.4	Übertragung an der motorischen Endplatte		12.5.4		
12.4.5	Liganden-gesteuerte Übertragung an zentralen Synapsen		12.5.4		
12.4.6	Second- messenger gesteuerte Übertragung an chemischen Synapsen		12.5.4, 12.5.3.2	12.5.3, 12.5.3.1	12.5.3
12.4.7	Wirkmechanismen verschiedener Transmitter		12.5.4, 12.5.3.1	12.5.1, 12.5.3.2	12.5.2, 12.5.3
12.4.8	synaptische Plastizität		12.5.4, 12.19.3.1	12.19.3	12.18.9.5
**12.5**	**Signalverarbeitung im Nervensystem**				
12.5.1	Elementarmechanismen		12.5.4		
12.5.2	Verarbeitung in Neuronen- populationen		12.5.4	12.5.4.3	
**12.6**	**Funktionsprinzipien sensorischer Systeme**		12.5.3, 12.5.3.2	12.5.3.1	12.19.2.1
12.6.1	allgemeine Aspekte	sensor	12.18.7		
12.6.2	Rezeptorpotential/Sensorpotential	sensor	12.18.7		
12.6.3	Transformation der Reize		x		
**13**	**Muskulatur**		12.4.3.1,12.4.3.4	12.4.3.2, 12.10.3.2	12.4.3.3
**13.1**	**Allgemeine Muskelphysiologie (s. a. GK Biochemie 22.1.1 und 30.2)**		12.10.3.4		
13.1.1	Myofilamente	muskelgewebe	12.4.3	12.10.3.3	12.10.3.4
13.1.2	Sarkolemm		12.4.3	12.10.3.3	12.10.3.4
13.1.3	sarkoplasmatisches Retikulum		12.4.3	12.10.3.4	
13.1.4	Sarkoplasma		12.4.3	12.10.3.4	
13.1.5	Energieumwandlung		12.4.3	12.10.3.4	
**13.2**	**Quergestreifte Muskulatur**		12.10.3.4		
13.2.1	allgemeine Grundlagen		12.10.3		
13.2.2	Skelettmuskel (s. a. 6.3.2 und GK Anatomie 2.6.1)		12.10.3		
13.2.3	Herzmuskel		12.11.2		
**13.3**	**Glatte Muskulatur (s. a. GK Anatomie 2.6.3)**		12.18.13.1		
**14**	**Vegetatives Nervensystem (VNS)**		12.18.2.7	12.18.13.1	
**14.1**	**Morphologische Grundlagen, Entwicklung, Wachstumsfaktoren, funktionelle Organisation (s. a. GK Ana- tomie 2.9)**		12.18.13	12.18.2	
**14.2**	**Zelluläre und molekulare Mechanismen der Signaltransduktion im VNS**		12.5.2, 12.5.1.7, 12.5.2.2	12.5.1, 12.5.1.8, 12.5.2.3	12.5.1.6, 12.5.2.1
14.2.1	synaptische Übertragung in den Ganglien		12.5.4		
14.2.2	Informationsübertragung von postganglionären Axonen auf Zielorgane		12.5.4, 12.5.3.2	12.5.3	12.5.3.1
14.2.3	Synthese und Abbau der Überträgerstoffe	transmitter	12.5.4	12.5.1	
**14.3**	**Funktionelle Organisation des VNS**		12.5.1	12.14.3.4	12.15.1.3
14.3.1	vegetative Steuerungen		12.18.13	12.16.3.4	
14.3.2	vegetative Reflexe (s. a. GK Ana- tomie 8.2.5)		12.18.3.3	12.16.3.4	12.18.11.2
14.3.3	supraspinale Kontrolle durch das Stammhirn (s. a. GK Ana- tomie 9.3.3)		12.18.13		
14.3.4	hypothalamische und limbische Steuerung		12.5.1, 12.19.6.3	12.5.1.3	12.5.3
14.3.5	enterisches Nervensystem		12.15.2	12.5.3.1	12.5.3.2
**15**	**Motorik**		12.18.7.2, 12.18.8.4, 12.18.8,	12.18.8, 12.18.11.1, 12.18.11.2	12.18.8.2, 12.18.12

**15.1**	**Allgemeine Organisation des motorischen Systems, Willkürbewegung**		12.18.3.3	12.18.12	
**15.2**	**Motorische Repräsentation auf dem Kortex**		12.18.10	12.18.12	
15.2.1	primärer motorischer Kortex (Area 4)		12.18.10	12.18.12	12.19.3.1
15.2.2	prä- und supplementär- motorischer Kortex (Area 6)		12.18.10	12.18.12	
15.2.3	motorischer Assoziations- kortex (u. a. Area 8)		12.18.10	12.18.12	
**15.3**	**Efferente Projektion der motorischen Kortizes**				
15.3.1	prinzipielle Verschaltungsmuster		12.18.12		
15.3.2	Projektion in subkortikale Gebiete		12.18.12		
**15.4**	**Neuronale Systeme des Rückenmarks**		12.5.4.3	12.18.11	12.18.13.1
15.4.1	Neuronentypen und ihre Lage		12.18.2		
15.4.2	Reflexsysteme des Rückenmarks		12.18.11		
15.4.3	Reflexsystem der Muskelspindelafferenzen		12.18.11		
15.4.4	Reflexsystem der Golgi-Sehnenorgane		12.18.11		
15.4.5	Reflexsystem der Beugereflexe		12.18.11		
**15.5**	**Motorische Funktionen des Hirnstamms, Gleichgewichtssinn**		12.18.8.1		
15.5.1	Augenmotorik (s. a. 17.1.8)		12.18.3		
15.5.2	vestibuläres System, Bewegungs- und Lagesinn		12.18.8	12.18.13	
15.5.3	Vestibulariskerne und motorische Funktionen		12.18.8	12.18.13	
15.5.4	andere motorische Funktionen des Hirnstamms		12.18.13		
**15.6**	**Basalganglien**		13.3.2		
15.6.1	Verschaltung, Informationsfluss		x		
15.6.2	Verarbeitungsprinzipien		12.5.4.3		
15.6.3	Störungen der Motorik		13.3.2.4		
**15.7**	**Zerebellum**				
15.7.1	Verschaltung, Informationsfluss		x		
15.7.2	Verarbeitungsprinzipien		x		
15.7.3	Störungen der Motorik		13.3.2.5		
**15.8**	**Integrale motorische Funktionen des Zentralnervensystems**				
15.8.1	Laufen und Gehen		12.18.12		
15.8.2	Stehen und Gleichgewicht		12.18.8	12.18.12	
15.8.3	Ergreifen eines Gegenstandes		12.18.12	12.19.3.1	
15.8.4	motorisches Lernen		x		
15.8.5	Sprache (s. a. 18.5 und 20.1.3)		12.19.7	12.14.2.4	12.19.7.3
**15.9**	**Störungen der Motorik**				
15.9.1	Muskeltonus		x		
15.9.2	Spastik		20.100	12.5.4.3	
15.9.3	Tremor		20.19		
15.9.4	Querschnittsverletzung des Rückenmarks		21.1.10.6		
**16**	**Somatoviszerale Sensorik**		12.18.7.2, 12.18.9.3	12.18.8, 12.19.2.1	12.18.8.4, 12.18.13.2, 12.19.6.7
**16.1**	**Funktionelle und morphologische Grundlagen**		12.18.7, 12.18.10.1	12.18.7.1	12.18.8.3
16.1.1	Einteilung, Modalitäten und Qualitäten		12.18.7	12.19.2	12.18.7.1
16.1.2	rezeptive und afferente Strukturen		12.18.7	12.19.2	12.18.7.1
16.1.3	spinale Strukturen		12.18.7	12.19.2	12.18.7.1
16.1.4	supraspinale Strukturen		12.18.7	12.19.2	12.18.7.1
**16.2**	**Tastsinn**		12.18.7	12.18.7.1	
16.2.1	Qualitäten		12.18.7	12.18.7.1	
16.2.2	Eigenschaften der Sensoren		12.18.7	12.18.7.1	
16.2.3	funktionelle Organisation		12.18.7	12.18.7.1	
16.2.4	Besonderheiten des Tastsinnes der Hand		12.18.7	12.18.7.1	
**16.3**	**Temperatursinn**		12.18.7	12.18.7.1	
16.3.1	Warm-/Kaltsensoren, afferente Bahnen und zentralnervöse Projektionen		12.18.7		
16.3.2	funktionelle Organisation des Warm-/Kaltsinnes		12.18.7		
**16.4**	**Tiefensensibilität**		12.18.8.1	12.18.11.1	12.18.11.2
1641	funktionelle Organisation		12.18.8		
16.4.2	biologische Bedeutung der Tiefensensibilität		12.18.8		
**16.5**	**Viszerale Sensorik**		12.18.11.2		
16.5.1	periphere und zentrale Sensoren		12.18.8		
16.5.2	viszerale Sensibilität		12.18.8	12.16.3.4	
16.5.3	viszerale Reflexe		12.18.11		
**16.6**	**Nozizeption**		12.18.9.2	12.18.9.4, 12.19.3.1	12.18.9.5
16.6.1	Nozizeptoren und periphere Nozizeption		12.18.9	12.18.9.1	
16.6.2	Nervenläsionen		12.18.9		
16.6.3	spinale und trigeminale Organisation der Nozizeption		12.18.9	12.5.4.3	
16.6.4	supraspinale Organisation von Nozizeption und Schmerz		12.18.9		
16.6.5	endogene Schmerzhemmung		12.18.9	12.5.4.3	
**17**	**Visuelles System**		12.18.10.2	12.19.2.1	
**17.1**	**Dioptrischer Apparat**	Grundlagen der Optik	12.2.1.6	12.18.3.6	12.18.11.2
17.1.1	physikalische Grundlagen (s. a. GK Physik 7)		12.18.3		
17.1.2	Auge als optisches System		12.18.3		
17.1.3	Abbildungsfehler		12.18.3		
17.1.4	Akkommodation		12.18.3		
17.1.5	Pupille		12.18.3	12.18.3.3	
17.1.6	Augeninnendruck		12.18.3		
17.1.7	Tränen (s. a. GK Anat. 10.4.3)		12.18.3	12.18.3.4	
17.1.8	Augenmotorik (s. a. 15.5.1 und GK Anat. 10.3.6)		12.18.3	12.18.3.3	
**17.2**	**Signalverarbeitung in der Retina**		12.18.3.5		

17.2.1	Aufbau der Retina (s. a. GK Anat. 10.3.4)		12.18.3	12.18.3.2	
17.2.2	Transduktionsprozess (s. a. GK Chemie/Bio- ch. 33.1)		12.18.3, 12.5.3.2	12.5.3	12.5.3.1
17.2.3	neuronale Verarbeitungsprozesse		12.18.3		
17.2.4	retinale Mechanismen des Farbensehens		12.18.3	12.18.3.7	
**17.3**	**Zentrale Repräsentation des visuellen Systems**				
17.3.1	Gesichtsfeld		12.18.3		
17.3.2	Sehbahn (s. a. GK Ana- tomie 9.8.1)		12.18.3		
**17.4**	**Informationsverarbeitung in der Sehbahn**				
17.4.1	Verschaltung der Sehbahn		12.18.3		
17.4.2	Retina		12.18.3		
17.4.3	Corpus geniculatum laterale		12.18.3		
17.4.4	visuelle Kortizes (Areae 17, 18; V1-V5)		12.18.3		
17.4.5	„extrastriäre Sehbahn“		12.18.3		
17.4.6	Tiefenwahrnehmung		12.18.3		
**18**	**Auditorisches System**		12.19.2.1		
**18.1**	**Physiologische Akustik**	Bedeutung von Schwingungen und Wellen in der Medizin	12.2.1.4		
18.1.1	Grundbegriffe (s. a. GK Physik 6.3)		12.18.4		
18.1.2	Testverfahren		12.18.4		
**18.2**	Äußeres Ohr, Gehörgang und Mittelohr (s. a. GK Anat. 11.2 und 11.3)		12.18.4	12.18.4.1	12.18.4.2, 12.18.4.3
**18.3**	Innenohr (s. a. GK Anat. 11.4.1)		12.18.4, 12.18.4.5	12.18.4.1, 12.18.4.6	12.18.4.4, 12.18.10.3
**18.4**	Zentrale Hörbahn und kortikale Repräsentation (s. a. GK Anat. 9.8.1)		12.18.4	12.18.4.6	12.18.4.7, 12.18.10.3
**18.5**	Sprachbildung und Sprachverständnis (s. a. 15.8.5)		12.18.4	12.19.7.3	
18.5.1	Stimmbildung (s. a. GK Anat. 5.4.11)		12.18.4	12.19.7.4	
18.5.2	Sprachverständnis		12.18.4		
**19**	**Chemische Sinne**		12.19.2.1		
**19.1**	**Grundlagen der chemischen Sinne**		12.18.5, 12.18.6.2	12.18.5.2, 12.18.11.2	12.18.6.1
19.1.1	Einteilung, morphologische Grundlagen und sensorische Funktionen		12.18, 12.18.6.2	12.18.6	12.18.6.1
19.1.2	Schutzreflexe, viszerale und sekretorische Reflexe	Reflex	12.18.11, 12.18.6.2	12.18.6, 12.18.11	12.18.6.1, 12.19.4.1
**19.2**	**Geschmack**		12.18.6.2		12.18.6.1
19.2.1	Geschmacksqualitäten und Psycho- physiologie des Geschmackssinns		12.18.6		
19.2.2	Sensoren		12.18.6, 12.5.3.2	12.5.3	12.5.3.1
19.2.3	zentrale Projektionen		12.18.6		
**19.3**	**Geruchssinn und trigeminaler chemischer Sinn**		12.18.5	12.18.5.2	
19.3.1	Sinnesmodalitäten, Qualitäten und Psychophysiologie des Geruchs		12.18.5		
19.3.2	Transduktionsprozesse (s. a. GK Chemie/Bio- ch. 33.2)		12.18.5, 12.5.3.2	12.5.3	12.5.3.1
19.3.3	Bahnen und zentral-nervöse Verarbeitung		12.18.5	12.18.5.1	
19.3.4	Assoziationsregionen für den Geruchssinn		12.18.5	12.18.5.3	12.19.6.7
**20**	**Integrative Leistungen des Zentralnervensystems**				
**20.1**	**Allgemeine Physiologie und funktionelle Anatomie der Großhirnrinde**		12.18.2.2		
20.1.1	Organisation der Großhirnrinde (s. a. GK Ana- tomie 9.7.3)		12.18		
20.1.2	kortikale Felder		12.18.10	12.19.3.1	12.19.9.5
20.1.3	kortikale Asymmetrie, Händig- keit und Sprachfunktionen		12.19.7	12.18.10	12.19.7.3
20.1.4	Analyse der Hirnrindenaktivität und tiefer liegender Kerngebiete (s. a. GK Psych./Soz. 1.3.6)		12.18.2		
**20.2**	Integrative Funktionen durch Interaktionen zwischen Hirnrinde und subkortikalen Hirnre- gionen		12.18.2.1	12.18.13.3	
20.2.1	zirkadiane Periodik		14b.5	12.18.13.1	
20.2.2	Bewusstsein (s. a. GK Psych./Soz. 1.4.1)		12.19.1	12.19.2	
20.2.3	Plastizität, Gedächtnis und Lernen (s. a. GK Psych./Soz. 1.4.2 und 1.4.3)		12.19.3, 12.19.3.3, 12.19.8.4	12.19.3.1, 12.19.3.4	12.19.3.2, 12.19.4.1
20.2.4	Antrieb, Motivation und Emotionen (s. a. GK Psych./Soz. 1.4.4 und 1.4.5)		12.18.12, 12.19.5.4	12.19.6, 12.19.5.8	12.19.3, 12.19.6.3, 12.19.6.7

3.6. Grundlagen der Medizinischen Psychologie und der Medizinischen Soziologie

	GK Psychologie und Soziologie	Synonym	Abgleich mit NKLM		
**1**	**Entstehung und Verlauf von Krankheiten**				
**1.1**	**Bezugssysteme von Gesundheit und Krankheit**		12.2.6.3		
1.1.1	Gesundheit und Krankheit		12.2.6, 12.2.6.2, 12.9.1.5, 12.10.3.7, 12.11.6.12, 12.12.2.5, 12.13.4.5	12.14.3.6, 12.15.4.3, 12.16.3.5, 12.17.3.3, 12.18.13.5, 12.18.2.10	12.18.3.8, 12.18.4.8, 12.18.5.3, 12.18.6.3, 12.18.7.3, 12.18.8.5
1.1.2	Die betroffene Person		12.2.6,12.2.6.5, 12.9.1.5,12.10.3.7, 12.11.6.12,12.12.2.5, 12.13.4.5,12.14.3.6	12.15.4.3,12.16.3.5, 12.17.3.3,12.18.13.5, 12.18.2.10,12.18.3.8, 12.18.4.8,12.18.5.3	2.18.6.3,12.18.7.3, 12.18.8.5,12.18.9.3, 12.19.2.1,12.19.2.2, 12.19.2.3
1.1.3	Die Medizin als Wissensund Handlungssystem	Menschen als soziales Wesen	12.2.6.3	12.20.1.1	
1.1.4	Die Gesellschaft		12.2.6,12.2.6.3	12.20.1.1, 12.20.1.2	
**1.2**	**Gesundheits- und Krankheitsmodelle**		12.9.1.5,12.10.3.7, 12.11.6.12,12.12.2.5, 12.13.4.5,12.14.3.6	12.15.4.3,12.16.3.5, 12.17.3.3,12.18.13.5, 12.18.2.10,12.18.3.8	12.18.4.8,12.18.5.3, 12.18.6.3,12.18.7.3, 12.18.8.5
1.2.1	Verhaltensmodelle		12.2.6,12.2.6.4, 12.18.9.4	12.18.9.5,12.19.10.3, 12.19.10.5	12.19.4.1,12.19.4.2
1.2.2	Psychobiologische Modelle	biopsychol	12.2.6,12.18.3.7, 12.18.9.4,12.19.2.1, 12.19.3.5	12.20.1.3,14c.1.1, 12.18.4.6,12.18.9.5, 12.19.2.3	16.1.6,12.18.9.3, 12.19.10,12.19.10.1, 12.19.10.2
1.2.3	Psychodynamische Modelle		15.12.2	12.19.10.6	
1.2.4	Sozialpsychologische Modelle	psychosozial	12.2.6,12.2.6.3, 12.19.5.5	12.19.5.6,12.19.10.3, 12.19.10.5	12.20.1.1,12.20.1.3, 12.20.1.2
1.2.5	Soziologische Modelle		12.2.1.2,12.2.6.3, 12.2.7.2	12.19.5.5,12.19.10.3	12.20.1.1,12.20.1.2, 12.20.1.3
**1.3**	**Methodische Grundlagen**		15.1.1		
1.3.1	Hypothesenbildung	Bedeutung hypothetischer Konstrukte	14a.2.1	12.2.6.1	
1.3.2	Operationalisierung		14a.2.1	12.2.6.1	12.19.8.1
1.3.3	Testdiagnostik		14a.2.1	12.2.6.1	
1.3.4	Untersuchungsplanung		14a.2.2		
1.3.5	Sozialwissenschaftliche Methoden der Datengewinnung		14a.2.2		
1.3.6	Psychobiologische Methoden der Datengewinnung		14a.2.2	12.19.10.2	
1.3.7	Datenauswertung und -interpretation		14a.3.1		
1.3.8	Ergebnisbewertung		14a.3.1		
**1.4**	**Theoretische Grundlagen**		12.9.1.5,12.10.3.7, 12.11.6.12,12.12.2.5, 12.13.4.5,12.14.3.6, 12.15.4.3	12.16.3.5,12.17.3.3, 12.18.13.5,12.18.2.10, 12.18.3.8,12.18.4.8, 12.18.5.3	12.18.6.4,12.18.7.3, 12.18.8.5, 12.18.12.3, 12.19.6.7
1.4.1	Psychobiologische Grundlagen	biopsych	12.2.6,12.18.2.3, 12.18.3.7,12.18.4.6	12.19.2.1,12.19.2.3, 12.19.2.4	12.19.3.5,12.19.4.4, 12.19.5.8
1.4.2	Lernen		12.18.9.4,12.18.9.5, 12.19.2.4,12.19.3.5	12.19.4,12.19.4.1, 12.19.4.2	12.19.4.4,12.19.4.5, 12.19.4.6
1.4.3	Kognition		12.19.2.1,12.19.2.2, 12.19.2.3,12.19.2.4, 12.19.3.3	12.19.3.4,12.19.3.5, 12.19.7.1,12.19.7.3, 12.19.8.1	12.19.8.2,12.19.8.3, 12.19.8.4,13.3.2
1.4.4	Emotion		12.19.2.4,12.19.3.5, 12.19.4.5,12.19.4.6, 12.19.5.8	12.19.6,12.19.6.1, 12.19.6.2,12.19.6.3, 12.19.6.4	12.19.6.5,12.19.6.7, 12.19.10.3,12.19.10.5
1.4.5	Motivation		12.19.2.4,12.19.3.5, 12.19.4.4,12.19.5	12.19.5.1,12.19.5.2, 12.19.5.3	12.19.5.4,12.19.5.7, 12.19.5.8
1.4.6	Persönlichkeit und Verhaltensstile		12.7.2,12.19.2.4, 12.19.3.5,12.19.9, 12.19.9.1	12.19.9.2,12.19.9.3, 12.19.9.4,12.19.9.5	12.19.10.1,12.19.10.2, 12.19.10.3,12.19.10.5
1.4.7	Entwicklung und primäre Sozialisation (Kindheit)	grundlegende Mechanismen der Entstehung und Aufrechterhaltung sozialer Strukturen und Institutionen	12.2.7.1,12.2.7.2, 12.7.2,12.7.2.1, 12.7.2.5	12.19.2.4,12.19.3.5, 12.19.7.2,12.19.8.2, 12.19.9.7	12.19.10.1,12.19.10.3, 12.20.1.1,12.20.1.2, 12.20.2.1
1.4.8	Entwicklung und Sozialisation im Lebenslauf (Adoleszenz, mittleres Erwachsenenalter, Senium) und sekundäre Sozialisation		12.2.7.1,12.2.7.2, 12.7.2,12.7.2.1, 12.7.2.5	12.19.2.4,12.19.3.5, 12.19.8.2,12.19.10.1	12.19.10.3,12.19.10.4, 12.20.1.1,12.20.1.2
1.4.9	Soziodemographische Determinanten des Lebenslaufs	demographisch	12.7.2.1,12.7.2.5, 12.19.2.4	12.19.3.5,12.19.10.1, 12.19.10.3	12.20.2.2,18.5.1
1.4.10	Sozialstrukturelle Determinanten des Lebenslaufs		12.2.7.2,12.7.2.1	12.7.2.5,12.20.2.1	12.20.2.2,12.20.2.3
**2**	**Ärztliches Handeln**				
**2.1**	**Arzt-Patient-Beziehung**				
2.1.1	Ärztliche Berufstätigkeit		14c.1.1		
2.1.2	Arztrolle		14c.1.1	12.20.1.2	
2.1.3	Krankenrolle	Gesundheits- und Krankheitserleben als subjektive Prozesse in ihren Wechsel-wirkungen	12.2.6.5,12.19.10.2, 12.19.10.3	12.19.10.5,12.19.5.5	12.20.1.2,14c.1.1
2.1.4	Kommunikation und Interaktion		14c.1.1	12.20.1.3	
2.1.5	Besonderheiten der Kommunikation und Kooperation		14c.1.1		
**2.2**	**Untersuchung und Gespräch**		12.19.4.3		
2.2.1	Erstkontakt		14c.5.4	12.19.5.5	
2.2.2	Exploration und Anamnese		14c.2.4	14c.2.8	12.20.1.3
2.2.3	Körperliche Untersuchung		14b.2	12.19.6.6	
**2.3**	**Urteilsbildung und Entscheidung**				
2.3.1	Grundlagen der diagnostischen Entscheidung		15.1		
2.3.2	Urteilsqualität		15.1		

**2.4**	**Interventionsformen und besondere medizinische Situationen**				
2.4.1	Ärztliche Beratung und Patientenschulung		14c.2.5,12.19.6.6	14c.2.8	14c.4.3
2.4.2	Psychotherapie		16.7.3,12.19.4.4	16.7.6	16.7.7
2.4.3	Intensiv- und Notfallmedizin		AP 17	12.19.6.6	12.19.10.6
2.4.4	Transplantationsmedizin und Onkologie		16.4.12	12.19.6.6	
2.4.5	Humangenetische Beratung und Reproduktionsmedizin		18.3.7	12.19.6.6	12.20.1.3
2.4.6	Sexualmedizin		12.19.6.6		
2.4.7	Tod und Sterben, Trauer		18.3.4	12.19.6.6	12.19.10.6
**2.5**	**Patient und Gesundheitssystem**				
2.5.1	Stadien des Hilfesuchens		12.20.2	12.19.5.7	12.20.2.2
2.5.2	Bedarf und Nachfrage		12.20.2		
2.5.3	Patientenkarrieren im Versorgungssystem		x		
2.5.4	Qualitätsmanagement im Gesundheitswesen		10.5		
**3**	**Förderung und Erhaltung von Gesundheit**				
**3.1**	**Prävention**		15.1.1		
3.1.1	Präventionsbegriff		16.1.2		
3.1.2	Modelle gesundheitsrelevanten	gesundheit	12.19.5	12.19.5.5	12.19.5.6
3.1.3	Primäre Prävention		16.1.2, 12.20.2.3	12.19.5.6	
3.1.4	Sekundäre Prävention		16.1.2	12.19.4.4	12.19.5.6
3.1.5	Tertiäre Prävention		16.1.2, 12.19.10.2, 12.19.10.3	12.19.5.6, 12.19.10.5	12.20.2.3, 12.20.1.3
3.1.6	Rehabilitation		16.8,12.20.2.3	12.19.5.6	12.20.1.3
**3.2**	**Maßnahmen**		12.20.2.3	15.1.1	
3.2.1	Gesundheitserziehung und Gesundheitsförderung		16.7.18, 12.20.2.3	19.1	12.19.5.6
3.2.2	Rehabilitation, Selbsthilfe und Pflege		14c.2.8	12.19.5.6	12.20.2.3

4. NKLM im Vergleich zum GK-2

4.1. GK-2 Teil 1: Gesundheitsstörungen

GK-2 Teil 1: Gesundheitsstörungen	Synonym	Abgleich mit NKLM		
**1.0 Allgemeine Symptome und Befunde**				
1.1 Abnorme Gewichtsabnahme		20.42		
1.2 Abnorme Gewichtszunahme		20.43		
1.3 Abnormer Körpergeruch	Geruch	20.11		
1.4 Adynamie	Schwäche, Erschöpfung, Müdigkeit	20.63	14b.5.7	
1.5 Blutungsneigung bzw. Blutungen	Blutungen	13.3.6.4, 14b.2.3, 17.6.1.4, 20.77	20.23, 21.1.11.28, 21.1.11.37, 21.1.5.20	21.1.7.9, 21.1.10.4, 21.1.11.11
1.6 Bösartige Neubildungen in der Familienanamnese	Neoplasie	20.10		
1.7 Dysmorphiezeichen	Fehlbildung	20.3	21.1.11.2	
1.8 Exsikkose	Flüssigkeitsstörung, Flüssigkeitsmangel	20.20	20.117	21.1.3.19
1.9 Fieber		20.27	16.5.15, 16.10.1	17.6.1
1.10 Hyperhydratation	Flüssigkeitsstörung	21.1.3.19		
1.11 Hypothermie	Unterkühlung	21.1.8.1		
1.12 Ikterus	Gelbsucht	16.3.26	20.34	
1.13 Leistungsminderung	Schwäche	20.63		
1.14 Nachtschweiß	Schwitzen	20118		
1.15 Ödeme		20.36		
1.16 Schüttelfrost		x		
1.17 Schwellung bzw. Verfärbung von Gliedmaßen	Schwarzverfärbung, Schwellung	14b.5.8	20.95	
1.18 Umschriebene Gewebeschwellung	Schwellung	13.3.6.5, 14b.2.4, 17.6.1.5, 20.61	20.78, 21.1.5.21, 21.1.7.10, 21.1.10.5	21.1.11.11, 21.1.11.28, 21.1.11.37
1.19 Vielzahl bzw. Wechsel von Beschwerden	Multi	20.64		
1.20 Wärmeintoleranz	Temperatur	20.2	21.1.11.3	
**2.0 Haut, Unterhaut, Haare, Schleimhaut, Lymphknoten**		20.118,	21.1.3.20	
2.1 Atrophie der Haut		16.5.16	16.10.2	17.6.2
2.2 Blasenbildung der Haut bzw. Schleimhaut	Blase	20.21		
2.3 Blässe		20.22		
2.4 Ekzem		20.89	16.3.27	
2.5 Erythem	Rötung	20.81		
2.6 Exanthem		20.81		
2.7 Haarausfall	Alopezie	21.1.8.43		
2.8 Hämatom	Flecken auf der Haut	20.57		
2.9 Hautblutungen	Flecken auf der Haut	20.29	14b.5.9	
2.10 Hautemphysem		13.3.6.6, 14b.2.5, 17.6.1.6, 20.79	21.1.7.11, 21.1.10.6, 21.1.11.11	21.1.11.28, 21.1.11.37, 21.1.5.22
2.11 Hautschuppung	Schuppig	20.89		
2.12 Hyperhidrose	Schwitzen	20.118	21.1.11.4	
2.13 Hypertrichose bzw. Hirsutismus	Haare	20.112	20.119	21.1.3.21
2.14 Hypohidrose		16.5.17	16.10.3	17.6.3
2.15 Lokalisierte Schwellung, Raumforderung, Knoten der Haut bzw. Unterhaut		20.61		
2.16 Lymphknotenvergrößerung		20.92		
2.17 Mamillare Hautveränderungen		16.3.28		
2.18 Nagelanomalien bzw. Nagelveränderungen	Nagel, Nägel	20.112		
2.19 Papelbildung		20.29		
2.20 Photosensibilität der Haut		x		
2.21 Pigmentierungsstörungen	Flecken auf der Haut	20.29		
2.22 Pruritus		20.52	14b.5.10	
2.23 Pustelbildung		13.3.6.7, 14b.2.6, 17.6.1.7, 20.80	20.29, 21.1.7.12, 21.1.10.7, 21.1.11.11	21.1.11.28, 21.1.11.37, 21.1.5.23
2.24 Schwellung bzw. Rötung der Hand		20.95		
2.25 Teleangiektasien		21.1.11.5		
2.26 Trockenheit der Haut		20.89	20.120	21.1.3.22
2.27 Ulkus der Haut bzw. Schleimhaut	trocken	20.120	16.5.18, 16.10.4	17.6.4
2.28 Urtikaria	Ulcera	20.29	20.61	21.1.8.40
2.29 Veränderungen des Hautreliefs	Haut	20.89	20.91	20.120
2.30 Vermehrte Hornbildung		16.3.29		
2.31 Wunde	Wund	20.120	20.121	
2.32 Wundheilungsstörung		x		
**3.0 Kreislaufsystem**				
3.1 Angina pectoris		20.107	16.5.43	
3.2 Claudicatio intermittens	pAVK	'14b.5.11	20.86, 20.120	21.1.1.4, 21.1.3.6
3.3 Einflussstauung		13.3.6.8, 14b.2.7, 17.6.1.8, 20.81	21.1.7.13, 21.1.10.8, 21.1.11.11	21.1.11.28, 21.1.11.37, 21.1.5.24
3.4 Erhöhter Blutdruck	Hypertonie	13.3.8.6, 13.3.9.1	21.1.1.17, 21.1.1.20	16.5.46
3.5 Kreislaufstillstand		21.1.11.6		
3.6 Niedriger Blutdruck		13.3.8	20.121	21.1.3.23
3.7 Pulslose Extremität	Embolie	21.1.1.3	16.5.19, 16.10.5	17.6.5

3.8 Schock		13.3.8.8, 17.6.1.5	20.22, 21.1.1.2	
3.9 Störungen des Herzrhythmus		16.3.12, 16.5.45	17.6.1	20.47, 21.1.1.13
3.10 Sturzanfall		21.1.1.16	16.3.30	
3.11 Synkope bzw. Kollaps		21.1.1.16	20.56	
3.12 Zyanose		20.124		
**4.0 Atmungssystem**				
4.1 Abnormes Sputum		x		
4.2 Aspiration		21.1.4.2	21.1.11.26	14b.5.12
4.3 Atemnot		13.3.6.9, 14b.2.8, 16.10.11, 17.6.1.2, 17.6.1.9	20.82, 20.7, 21.1.7.14, 21.1.10.9	21.1.11.11, 21.1.11.28, 21.1.11.37, 21.1.5.25
4.4 Atemrhythmusstörungen		20.7	21.1.4.16	
4.5 Atemstillstand		17.5.4	21.1.11.7	
4.6 Behinderte Nasenatmung		20.16	20.122,	21.1.3.24
4.7 Bradypnoe		20.7	16.5.20, 16.10.6	17.6.6
4.8 Fassthorax		x		
4.9 Giemen		x		
4.10 Hämoptoe	Husten	20.49	16.3.31	
4.11 Hämoptyse		20.49		
4.12 Husten		20.49	16.5.50	16.10.11
4.13 Hyperventilation		20.7	20.56	20.106
4.14 Inverse Atmung		x		
4.15 Orthopnoe		14b.5.13		
4.16 Paradoxe Atmung		13.3.6.10, 14b.2.9, 17.6.1.10, 20.83	21.1.7.15, 21.1.10.10, 21.1.11.11	21.1.11.28, 21.1.11.37, 21.1.5.26
4.17 Pfeifende Atmung		x		
4.18 Rasselgeräusche		21.1.11.8		
4.19 Schlafapnoe		20.123, 20.63	21.1.4.16	21.1.3.25
4.20 Schnarchen		16.5.21	16.10.7	17.6.7
4.21 Singultus		x		
4.22 Stöhnende Atmung ("Knorksen")		x		
4.23 Stridor		16.3.32		
4.24 Tachypnoe		x		
4.25 Trichterbrust		x		
4.26 Trommelschlegelfinger		x		
4.27 Uhrglasnägel		x		
**5.0 Verdauungssystem**		14b.5.14		
5.1 Aufstoßen		13.3.6.11, 14b.2.10, 17.6.1.11, 20.84	21.1.7.16, 21.1.10.11, 21.1.11.11	21.1.11.28, 21.1.11.37, 21.1.5.27
5.2 Belegte Zunge		x		
5.3 Blutiger Stuhl		20.77	21.1.11.9	
5.4 Defäkationsschmerzen		20.124	21.1.3.26	
5.5 Diarrhoe	Stuhlgang	16.5.22, 16.10.8	17.6.8	20.113
5.6 Erbrechen		20.110	16.5.53	
5.7 Foetor ex ore	Geruch	20.11		
5.8 Globusgefühl		16.3.33		
5.9 Hämatemesis		20.110		
5.10 Hypersalivation		x		
5.11 Miserere		x		
5.12 Mundtrockenheit		x		
5.13 Obstipation		21.1.7.41	14b.5.15	20.113
5.14 Peranale Blutung		13.3.6.12, 14b.2.11, 17.6.1.12, 20.85	20.77, 21.1.7.17, 21.1.10.12, 21.1.11.11	21.1.11.28, 21.1.11.37, 21.1.5.28
5.15 Regurgitation von Speisebrei		x		
5.16 Schluckstörungen		20.84	16.10.10	21.1.11.10
5.17 Sodbrennen		16.5.51, 16.4.64,	16.4.58, 20.125	21.1.7.10, 21.1.3.27
5.18 Stuhlinkontinenz		21.1.7.36	16.5.23, 16.10.9	17.6.9
5.19 Teerstuhl		20.77	21.1.7.10	21.1.3.27
5.20 Übelkeit		20.110	16.5.53	
5.21 Veränderungen der Stuhlgewohnheiten bzw. der Stuhlbeschaffenheit		20.113	16.3.34	
5.22 Vorfall von Mastdarm bzw. After		x		
5.23 Zungenbrennen		x		
**6.0 Abdomen**				
6.1 Abdominelle Abwehrspannung	Abdomen	21.1.7.5		
6.2 Aszites	Schwellung des Bauches	13.3.10	14b.5.16	
6.3 Hepatomegalie		13.3.6.13, 14b.2.12, 17.6.1.13, 20.86	21.1.7.18, 21.1.10.13, 21.1.11.11	21.1.11.28, 21.1.11.37, 21.1.5.29
6.4 Leistenschwellung	Schwellung	20.96		
6.5 Meteorismus bzw. Blähungen		20.93	21.1.11.11	
6.6 Resistenz im Abdomen	Schwellung	20.105, 20.126	21.1.7.5, 21.1.3.28	
6.7 Splenomegalie		16.5.24	17.6.10	16.10.10
6.8 Störungen der Peristaltik	Motilität	13.3.10		
**7.0 Ernährungsstörungen**				

7.1 Abneigung gegen bestimmte Nahrungsmittel		16.3.35	20.5, 20.69	21.1.7.29
7.2 Anorexie bzw. Untergewicht		21.1.3.28		
7.3 Appetitlosigkeit		20.5		
7.4 Fehl-Ernährung		20.111	21.1.3.28	
7.5 Gedeihstörung		20.119	20.26	21.1.3.11
7.6 Nahrungsverweigerung	Appetitlosigkeit	20.5	14b.5.17	
7.7 Polydipsie		13.3.6.14, 14b.2.13, 17.6.1.14, 20.87	21.1.7.19, 21.1.10.14, 21.1.11.11,	21.1.11.28, 21.1.11.37, 21.1.5.30
7.8 Polyphagie bzw. Essattacken	Gewichtszunahme, Binge-Eating	20.43		
7.9 Übergewicht	Gewichtszunahme	20.43	21.1.11.12	
7.10 Unverträglichkeit für bestimmte Nahrungsmittel		20.69	20.127	21.1.3.29
**8.0 Stoffwechsel, Endokrinium, Immunsystem**		16.5.25	16.10.11,	17.6.11
8.1 Abnormer Körpergeruch		20.11		
8.2 Akromegalie-Symptome		x		
8.3 Allergische Reaktion	allergisch	16.3.36, 16.5.41	20.52, 20.81	21.1.4.23, 21.1.8.10
8.4 Galaktorrhoe		x		
8.5 Gynäkomastie		21.1.3.16		
8.6 Infektneigung		21.1.5.12		
8.7 Libidoverlust		20.103		
8.8 Schilddrüsenvergrößerung	Struma	21.1.3.1	14b.5.18	
8.9 Stammfettsucht	Cushing	13.3.6.15, 14b.2.14, 17.6.1.15, 20.88	21.1.7.20, 21.1.10.15, 21.1.11.11	21.1.11.28, 21.1.11.37, 21.1.5.31, 21.1.3.21
**9.0 Skelett, Bewegungssystem**				
9.1 Abnorme Beweglichkeit	Bewegungsstörung	20.19, 20.100	21.1.11.13	21.1.10.33
9.2 Frakturneigung		20.128	21.1.3.30	
9.3 Gangstörung	Gehstörung	20.33	16.5.26, 16.10.12	17.6.12
9.4 Gelenkinstabilität		21.1.2.18		
9.5 Gelenkschwellung		20.35		
9.6 Gelenksteife		20.100	16.3.37	
9.7 Haltungsfehler		x		
9.8 Kieferklemme bzw. Kiefersperre		x		
9.9 Morgensteifigkeit		20.100		
9.10 Muskelatrophie		13.2.1	21.1.10.35	
9.11 Muskelhypertrophie		13.2.1	14b.5.19	
9.12 Muskelkontraktur		13.3.5, 13.3.6.16, 114b.2.15, 17.6.1.16	21.1.7.21, 21.1.10.16, 21.1.11.11, 20.89	21.1.11.28, 21.1.11.37, 21.1.5.32
9.13 Skelettdeformitäten		21.1.2.13	21.1.2	16.4.52
**10.0 Harntrakt**		21.1.11.14		
10.1 Abnormer Harngeruch		20.129	21.1.3.31	
10.2 Anurie	Urin	20.117, 20.62	16.5.27, 16.10.13	17.6.13
10.3 Ausfluss aus der Harnröhre	Ausfluss	20.39		
10.4 Erschwerte Miktion	Urin	20.62		
10.5 Hämaturie		20.45	16.3.38	
10.6 Harninkontinenz		20.62		
10.7 Harntransportstörungen		20.117		
10.8 Harnverfärbung bzw. -trübung		20.45		
10.9 Harnverhaltung		20.117		
10.10 Nykturie		14b.5.20		
10.11 Oligurie		13.3.6.17, 14b.2.16, 17.6.1.17, 20.90	20.117,' 21.1.7.22, 21.1.10.17, 21.1.11.11	21.1.11.28, 21.1.11.37, 21.1.5.33
10.12 Pollakisurie		20.62		
10.13 Polyurie		20.62	21.1.11.15	
10.14 Schaumiger Harn		20.130	21.1.3.32	
10.15 Schmerzhafte Miktion		16.5.28, 16.10.14	17.6.14	20.62
**11.0 Genitalorgane allgemein**				
11.1 Fertilitätsstörungen		21.1.6.38		
11.2 Genitalblutungen		16.3.39		
11.3 Genitale Fehlbildungen		20.3	21.1.11.30	
11.4 Sterilität		21.1.6.38		
**12.0 Männliche Genitalorgane**				
12.1 Hämospermie		x		
12.2 Hodenfehllage		21.1.6.8	14b.5.21	
12.3 Phimose bzw. Paraphimose		13.3.6.18, 14b.2.17, 17.6.1.18, 20.91	21.1.7.23, 21.1.10.18, 21.1.11.11	21.1.11.28, 21.1.11.37, 21.1.5.34, 21.1.6.10
12.4 Schwellung im Skrotalbereich		20.94		
12.5 Störungen der Hodenentwicklung		21.1.11.16		
**13.0 Weibliche Genitalorgane**		20.131	21.1.3.33	
13.1 Amenorrhoe	Menstruation	16.5.29, 16.10.15	17.6.15	20.1
13.2 Äußerer Vorfall der inneren weiblichen Geschlechtsorgane		21.1.6.30		
13.3 Dysmenorrhoe	Menstruation	20.1		
13.4 Dyspareunie		20.38	16.3.40	
13.5 Fluor genitalis	Ausfluss	20.39		
13.6 Klimakterische Störungen	Klimakterium	21.1.6.29		

13.7 Mamma-Knoten		20.55	21.1.8.5	
13.8 Mastodynie	Schmerzen in der weiblichen Brust	20.87		
13.9 Menstruationsstörungen		20.1	14b.5.22	
13.10 Mittelschmerz		13.3.6.19, 14b.2.18, 17.6.1.19, 20.92	20.01, 21.1.7.24, 21.1.10.19, 21.1.11.11	21.1.11.28, 21.1.11.37, 21.1.5.35
13.11 Pathologische Sekretion aus der Mamma		x		
13.12 Prämature Menopause		21.1.6.29	21.1.11.17	
13.13 Prämenstruelles Syndrom		20.132	21.1.3.34	
**14.0 Schwangerschaft, Wochenbett, Säuglingsalter**		16.5.30,	16.10.16	17.6.16
14.1 Abnormer Fontanellen-Tastbefund		x		
14.2 Atemnot beim Neugeborenen		21.1.11.27	21.1.11.26	
14.3 Fruchtwasserabgang		21.1.11.4	16.3.41	
14.4 Frühgeburtlichkeit		20.78		
14.5 Geburtsunmögliche Lagen		21.1.11.2		
14.6 Habitueller Abort		x		
14.7 Neugeborenen-Hyperexzitabilität		21.1.11		
14.8 Perinatale Asphyxie		21.1.11	14b.5.23	
14.9 Postpartale Blutung		13.3.6.20, 14b.2.19, 17.6.1.20, 20.93	21.1.7.25, 21.1.10.20, 21.1.11.11	21.1.11.28, 21.1.11.37, 21.1.5.36, 21.1.11.11
14.10 Schwangerschaftsbedingte Beschwerden		20.79		
14.11 Stillschwierigkeiten		21.1.11.18		
14.12 Verminderte Kindsbewegungen		20.79	20.133	21.1.3.35
14.13 Vorzeitige Wehen		20.78	16.5.31, 16.10.17	17.6.17
**15.0 Wachstum, Entwicklung**				
15.1 Enkopresis		21.1.6.43		
15.2 Enuresis		21.1.6.43	16.3.42	
15.3 Hochwuchs	Makrosomie, Wachstum, Großwuchs	20.119		
15.4 Kleinwuchs	Wachstum, Kleinwuchs	20.119		
15.5 Makrozephalie	Hydrozephalus	21.1.11.39		
15.6 Mikrozephalie	Hydrozephalus	21.1.11.39		
15.7 Motorische Entwicklungsstörungen		20.119	14b.5.24	
15.8 Psychische Entwicklungsstörungen		13.3.6.21, 14b.2.20, 17.6.1.21, 20.94	21.1.7.26, 21.1.10.21, 21.1.11.11	21.1.11.28, 21.1.11.37, 21.1.5.37, 21.1.10.51
15.9 Sprachliche Entwicklungsstörungen		20.101		
15.10 Störungen der Pubertätsentwicklung		20.119	21.1.11.19	
15.11 Wachstumsstörungen		20.119	20.134	21.1.3.36
**16.0 Augen**		16.5.32	16.10.18	17.6.18
16.1 Abnorme Bindehautsekretion		x		
16.2 Blepharospamus		x		
16.3 Doppelbilder		20.25	16.3.43	
16.4 Einschränkungen des Gesichtsfeldes	Sehstörung	20.25		
16.5 Exophthalmus		x		
16.6 Flimmern vor den Augen	Sehstörung	20.25		
16.7 Fremdkörperbeschwerden des äußeren Auges		16.3.9		
16.8 Hornhauttrübung	Keratokonjunktivitis	21.1.9.18	14b.5.25	
16.9 Lichtscheu		13.3.6.22, 14b.2.21, 17.6.1.22, 20.95	21.1.7.27, 21.1.10.22, 21.1.11.11	21.1.11.28, 21.1.11.37, 21.1.5.38
16.10 Lidschwellung		x		
16.11 Linsentrübung	Katarakt	21.1.9.22	21.1.11.20	
16.12 Papillenschwellung	Papille	21.1.9.28	20.135	21.1.3.37
16.13 Ptosis		16.5.33	16.10.19	17.6.19
16.14 Pupillenstörungen		x		
16.15 Rotes Auge		20.80		
16.16 Schielen	Strabismus	20.25	16.4.22	16.3.44
16.17 Sicca-Symptomatik		21.1.9.18		
16.18 Sonnenuntergangsphänomen		x		
16.19 Störung des Sehvermögens bzw. Blindheit	Sehstörung	20.25		
16.20 Störungen von Bewegungen bzw. der Beweglichkeit des Auges	Blickmotorik	13.3.2		
16.21 Tränenträufeln		16.5.30	14b.5.26	
16.22 Verzerrtsehen	Sehstörung	13.3.6.23, 14b.2.22, 17.6.1.23, 20.96	20.25, 21.1.7.28, 21.1.10.23, 21.1.11.11	21.1.11.28, 21.1.11.37, 21.1.5.39
**17.0 Ohren**				
17.1 Ausfluss bzw. Blutung aus dem Gehörgang		20.13	21.1.11.21	
17.2 Gehörgangsfremdkörper		16.3.10	20.136	21.1.3.38
17.3 Störungen des Hörvermögens bzw. Taubheit	Hörstörung, Schallempfindungs-/leitungsstörung	16.4.27, 16.10.7, 20.48	16.5.34, 16.10.20, 17.6.20	21.1.9.2, 21.1.9.3
17.4 Tinnitus		21.1.9.6		
**18.0 Nase, Geruchs- und Geschmackssinn**				
18.1 Abnorme Nasensekretion	Ausfluss aus der Nase	20.14	16.3.45	
18.2 Borkenbildung in der Nase		x		
18.3 Epistaxis		20.14	21.1.4.24	
18.4 Nasenfremdkörper		20.16		
18.5 Störungen des Geruchs- bzw. Geschmackssinnes		13.3.2.29		
**19.0 Neurologische Störungen**		14b.5.27		

19.1 Apraxie	Bewegungsstörung	13.3.6.24, 14b.2.23, 17.6.1.24, 20.97	20.19, 20.33 21.1.7.29, 21.1.10.24	21.1.11.11, 21.1.11.28, 21.1.11.37, 21.1.5.40
19.2 Ataxie		x		
19.3 Dystonien (generalisiert, fokal)		21.1.11.22		
19.4 Faszikulationen		20.137	21.1.3.39	
19.5 Hirntod		16.5.35	16.10.21	17.6.21
19.6 Hyperkinesen	Bewegungsstörung	20.19		
19.7 Hypokinese bzw. Hypomimie	Bewegungsstörung	20.19		
19.8 Krampfanfall		20.58	16.3.46	21.1.10.19
19.9 Lähmungen		20.60		
19.10 Liquorrhoe		20.14		
19.11 Meningismus		20.68		
19.12 Muskelkrämpfe		21.1.10.20		
19.13 Muskuläre Hypertonie		14b.5.28		
19.14 Muskuläre Hypotonie	Muskelschwäche	13.3.6.25, 14b.2.24, 20.98, 20.66	17.6.1.25, 21.1.7.30, 21.1.10.25, 21.1.11.11	21.1.11.28, 21.1.11.37, 21.1.5.41
19.15 Myoklonien		x		
19.16 Opisthotonus		21.1.11.23		
19.17 Reflexanomalien		20.138	21.1.3.40	
19.18 Rigor		16.5.36, 16.10.22	17.6.22	20.100
19.19 Schwindel bzw. Gleichgewichtsstörungen		16.5.16, 16.10.2	20.97	21.1.10.27
19.20 Sensibilitätsstörungen		20.106		
19.21 Spastik		20.100	16.3.47	
19.22 Tremor		20.19		
**20.0 Sprache, Sprechen, Stimme**				
20.1 Aphasie		20.101	21.1.10.48	
20.2 Dysarthrophonie bzw. Dysglossie		x		
20.3 Heiserkeit		14b.5.29		
20.4 Mutismus		13.3.6.26, 14b.2.25, 17.6.1.26, 20.99	21.1.7.31, 21.1.10.26, 21.1.11.11	21.1.11.28, 21.1.11.37, 21.1.5.42
20.5 Stottern bzw. Poltern	Sprachstörung	20.101	21.1.10.48	
**21.0 Schmerzen**		21.1.11.24		
21.1 Akuter (plötzlicher) Schmerz		16.5.13, 16.10.3	20.139	21.1.3.41
21.2 Augenschmerzen		20.12	16.5.37, 16.10.23	17.6.23
21.3 Bauchschmerzen		20.15		
21.4 Brustschmerzen	Thoraxschmerzen	20.107		
21.5 Chronischer Schmerz	Schmerzen	16.3.48	20.18, 20.75	21.1.2.44, 21.1.10.22
21.6 Flankenschmerzen		20.28		
21.7 Gelenkschmerzen		20.86		
21.8 Gesichtsschmerz		20.40		
21.9 Halsschmerzen		20.44		
21.10 Hodenschmerzen		20.38	14b.5.30	
21.11 Ischialgie	Schmerzen der Extremitäten	13.3.6.27, 14b.2.26, 17.6.1.27, 20.100	20.82, 20.86, 21.1.7.32, 21.1.10.27	21.1.11.11, 21.1.11.28, 21.1.11.37, 21.1.5.43
21.12 Knochenschmerzen	Schmerzen der Extremitäten	20.86	20.88	
21.13 Kolikartige Schmerzen	Bauchschmerzen	20.15	21.1.11.25	
21.14 Kopfschmerzen		20.57, 20.140	21.1.10.21, 21.1.10.23	21.1.3.42
21.15 Leistenschmerzen		16.5.38	16.10.24	17.6.24
21.16 Multilokuläre Schmerzen		20.37		
21.17 Myalgie		20.65		
21.18 Nackenschmerzen		20.67	16.3.49	
21.19 Neuralgiforme Schmerzen		20.37		
21.20 Ohrenschmerzen		20.70		
21.21 Phantomschmerz		20.75		
21.22 Radikulärer Schmerz	Rückenschmerzen	21.1.6.17		
21.23 Rücken- bzw. Kreuzschmerzen		20.82	14b.5.31	
21.24 Säuglingskolik	Bauchschmerzen	13.3.6.28, 14b.2.27, 17.6.1.28	20.101, 20.15, '21.1.7.33, 21.1.10.28	21.1.11.11, 21.1.11.28, 21.1.11.37, 21.1.5.44
21.25 Schmerzen bei der Atmung	Thoraxschmerzen	20.107		
21.26 Schmerzen im Bereich der Extremitäten		20.86	21.1.11.26	
21.27 Schmerzen im Bereich von Becken bzw. Damm	Schmerzen im Bereich der Vulva	20.141	21.16.17	21.1.3.43
21.28 Schmerzen in Zusammenhang mit der Nahrungsaufnahme		16.5.39	16.10.25	17.6.25
21.29 Tenesmen	Beschwerden beim Stuhlgang	20.113		
21.30 Zahnschmerz	Schmerzen in Mund und Kiefer, Gesichtsschmerzen	20.88	20.40	
21.31 Zentraler Schmerz	Phantomschmerz, generalisierter Schmerz, ohne org. Korrelat	20.37	16.3.50	
**22.0 Psychische Störungen, Verhaltensstörungen, psychosoziale Probleme**				
22.1 Aggressivität		x		

22.2 Angst bzw. Phobie		20.4,<br>20.47	16.5.22	21.1.10.53
22.3 Anhedonie		14b.5.6		
22.4 Antriebsstörung		14b.5.7	14b.5.32	
22.5 Aufmerksamkeits- bzw. Konzentrationsstörungen		13.3.6.29,<br>14b.5.3,<br>14b.2.28,<br>17.6.1.29,<br>20.102	21.1.7.34,<br>21.1.10.29,<br>21.1.11.11,<br>21.1.11.28	21.1.11.37,<br>21.1.5.45,<br>21.1.10.67,<br>21.1.10.53
22.6 Autoaggressives Verhalten		14b.5.10		
22.7 Bewusstseinsstörungen (qualitativ, quantitativ)		20.20,<br>14b.5.2	17.6.1.1	21.1.11.27
22.8 Bindungs- bzw. Beziehungsstörungen		20.142	21.1.3.44	
22.9 Denkstörungen		16.5.40	16.10.26	17.6.26
22.10 Depressivität		16.5.21,<br>20.4,<br>20.5,<br>20.22,<br>20.32,<br>20.63	20.66,<br>20.71,<br>20.73,<br>20.75,<br>20.83,	20.98,<br>20.99,<br>20.102,<br>20.116,<br>21.1.10.45
22.11 Dissoziales Verhalten		21.1.10.61		
22.12 Dissoziation (Bewusstsein)		20.20	16.3.51	
22.13 Ermüdungssyndrom	Müdigkeit	20.63		
22.14 Flashbacks	posttraumatisch	21.1.10.55		
22.15 Gedächtnisstörungen		20.32		
22.16 Ich-Störungen		14b.5.5		
22.17 Innere Anspannung bzw. innere Unruhe		14b.5.33		
22.18 Interessenverarmung		13.3.6.30,<br>14b.2.29,<br>17.6.1.30,<br>20.103	21.1.7.35,<br>21.1.10.30,<br>21.1.11.11	21.1.11.28,<br>21.1.11.37,<br>21.1.5.46
22.19 Katatonie	kataton	21.1.10.66		
22.20 Konfabulation		21.1.11.28		
22.21 Körperschemastörung		20.143	21.1.3.45	
22.22 Motorische Unruhe bzw. Bewegungsdrang	Bewegungsstörung,<br>restless leg	21.1.10.33	16.5.41,<br>16.10.27	17.6.27
22.23 Orientierungsstörungen		x		
22.24 Parathymie		x		
22.25 Probleme im sozialen Umfeld	sozialer Rückrug,<br>Sozialverhalten	16.3.52	20.99	21.1.10.50
22.26 Psychische Verstimmung	Dysthymie	20.63	21.1.10.46	
22.27 Schlafstörungen		20.83	21.1.10.62	
22.28 Schul- bzw. Lernschwierigkeiten		20.26		
22.29 Sozialer Rückzug		20.99		
22.30 Stimmungsschwankungen	Zyklothymia	21.1.10.44	14b.5.34	
22.31 Störungen der Krankheitsbewältigung einschl. Non-Compliance		12.19.10,<br>14b.2.30,<br>17.6.1.31,<br>20.104	21.1.7.36,<br>21.1.10.31,<br>21.1.11.11,<br>21.1.11.28	21.1.11.37,<br>21.1.5.47,<br>13.3.6.31
22.32 Störungen der Sexualität (Funktion, Verhalten, Identität)		20.103		
22.33 Stupor		x	21.1.11.29	
22.34 Suizidalität		14c.3.2,<br>14b.5	15.12.2,<br>'18.3.4	21.1.3.46,<br>20.144
22.35 Tagesschläfrigkeit	Müdigkeit	20.71,<br>20.51,<br>20.63	16.5.42,<br>16.10.28	17.6.28,<br>21.1.10.72
22.36 Tics bzw. Stereotypien		20.123	21.1.10.68	
22.37 Verlangsamung bzw. herabgesetztes Reaktionsvermögen		20.116		
22.38 Verwirrtheit	Verwirrung, Delir	20.20	16.3.2	16.3.53
22.39 Wahnsymptome	Wahn	20.24		
22.40 Wahrnehmungsstörungen bzw. Halluzinationen		20.24		
22.41 Zwangsgedanken bzw. Zwangshandlungen	Zwang	16.5.22	20.123	21.1.10.57

4.2. GK-2 Teil 2: Krankheitsbilder

GK-2 Teil 2: Krankheitsbilder	Synonym	Abgleich mit NKLM		
**A00-A09 Infektiöse Darmkrankheiten**	Gastroenteritis	20.15	20.110	21.1.7.28
**A15-A19 Tuberkulose**	Pulmonale und extrapulmonale Tuberkulose	21.1.4.14		
**A20-A28 Bestimmte bakterielle Zoonosen**	Zoonose	21.1.10.32	20.61	
A20 Pest		x		
A21 Tularämie		x		
A22 Anthrax [Milzbrand]		x		
A23 Brucellose		x		
A26 Erysipeloid	Entzündung der Haut	21.1.8.42		
A27 Leptospirose		x		
A28 Sonstige bakterielle Zoonosen, anderenorts nicht klassifiziert		21.1.10.32		
**A30-A49 Sonstige bakterielle Krankheiten**				
A31 Infektion durch sonstige Mykobakterien		x		
A32 Listeriose		x		
A35 Sonstiger Tetanus		21.1.2.30		
A36 Diphtherie		x		
A37 Keuchhusten		21.1.4.27		
A38 Scharlach		21.1.8.39	20.81	
A39 Meningokokkeninfektion	Meningitis	20.20, 20.24	20.68	21.1.10.14, 21.1.11.19
A40 Streptokokkensepsis	Sepsis neonatal	21.1.11.19	21.1.1.1	
A41 Sonstige Sepsis		21.1.1.1		
A42 Aktinomykose		x		
A44 Bartonellose		x		
A46 Erysipel [Wundrose]		21.1.8.42	20.81	20.36
A48 Sonstige bakterielle Krankheiten, anderenorts nicht klassifiziert	Staphylococcal scaled skin syndrome,toxisches Schocksyndrom	21.1.8.18		
A49 Bakterielle Infektion nicht näher bezeichneter Lokalisation		x		
**A50-A64 Infektionen, die vorwiegend durch Geschlechtsverkehr übertragen werden**	Lues, Gonorrhoe, Chlamydien, Syphilis, Neurolues	21.1.6.35	21.1.6.33	21.1.6.34
**A65-A69 Sonstige Spirochätenkrankheiten**	Borreliose	21.1.2.40		
A69 Sonstige Spirochäteninfektionen		21.1.2.40		
**A70-A74 Sonstige Krankheiten durch Chlamydien**		21.1.6.35		
**A75-A79 Rickettsiosen**		x		
**A80-A89 Virusinfektionen des Zentralnervensystems**				
A80 Akute Poliomyelitis [Spinale Kinderlähmung]	Poliomyelitis	21.1.10.30		
A81 Atypische Virus-Infektionen des Zentralnervensystems		x		
A82 Tollwut [Rabies]		21.1.10.31		
A83 Virusenzephalitis, durch Moskitos [Stechmücken] übertragen		21.1.10.14		
A84 Virusenzephalitis, durch Zecken übertragen	Sonstige Zoonosen (z.B. Toxoplasmose, Leishmaniose, Frühsommer-Meningoenzephalitis)	21.1.10.14	21.1.10.32	
A87 Virusmeningitis		21.1.8.9		
**A90-A99 Durch Arthropoden übertragene Viruskrankheiten und virale hämorrhagische Fieber**		x		
**B00-B09 Virusinfektionen, die durch Haut- und Schleimhautläsionen gekennzeichnet sind**	Herpesinfektionen und Reaktivierung, Herpesenzephalitis, Warzen, humane Papillomviren (HPV), Molluscum contagiosum, Erythema infectiosum (Ringelröteln), Drei-Tage-Fieber (Exanthema subitum), Röteln ,Masern	21.1.8.9, 21.1.8.29	21.1.8.31, 21.1.8.32	21.1.8.36, 21.1.8.37
**B15-B19 Virushepatitis**	Infektiöse Hepatitiden	21.1.7.23	20.34	21.1.7.44
**B20-B24 HIV-Krankheit [Humane Immundefizienz-Viruskrankheit]**		21.1.5.12		
B20 Infektiöse und parasitäre Krankheiten infolge HIVKrankheit [Humane Immudefizienz-Viruskrankheit]		21.1.5.12		
B21 Bösartige Neubildungen infolge HIV-Krankheit [Humane Immundefizienz-Viruskrankheit]		21.1.5.12		
B22 Sonstige näher bezeichnete Krankheiten infolge HIVKrankheit[Humane Immundefizienz-Viruskrankheit]		21.1.5.12		
B23 Sonstige Krankheitszustände infolge HIV-Krankheit [Humane Immundefizienz-Viruskrankheit]		21.1.5.12		

B24 Nicht näher bezeichnete HIV-Krankheit [Humane Immundefizienz-Viruskrankheit]		21.1.5.12		
**B25-B34 Sonstige Viruskrankheiten**				
B25 Zytomegalie		x		
B26 Mumps		21.1.7.30		
B27 Infektiöse Mononukleose		21.1.5.7		
B30 Viruskonjunktivitis		21.1.9.16		
B34 Viruskrankheit nicht näher bezeichneter Lokalisation		x		
**B35-B49 Mykosen**		20.112		
B35 Dermatophytase [Tinea]	Dermatophytie	21.1.8.16		
B36 Sonstige oberflächliche Mykosen		20.112		
B37 Kandidose		21.1.8.11		
B44 Aspergillose		x		
B45 Kryptokokkose		x		
**B50-B64 Protozoenkrankheiten**	Sonstige Zoonosen (z.B. Toxoplasmose, Leishmaniose, Frühsommer-Meningoenzephalitis), Malaria	21.1.10.32	21.1.5.4	
**B65-B83 Helminthosen**	Wurmerkrankungen, Kolitis, Enterokolitis, Gastreoenteritis	21.1.7.28		
B65 Schistosomiasis [Bilharziose]		21.1.7.28		
B67 Echinokokkose		21.1.7.28		
B68 Taeniasis		21.1.7.28		
B69 Zystizerkose		21.1.7.28		
B77 Askaridose		21.1.7.28		
B80 Enterobiasis		21.1.7.28		
**B85-B89 Pedikulose [Läusebefall], Akarinose [Milben befall] und sonstiger Parasitenbefall der Haut**				
B85 Pedikulose [Läusebefall] und Phthiriasis [Filzläusebefall]	Kopflaus- und Filzlausbefall	21.1.8.34		
B86 Skabies		21.1.8.38		
**C00-C14 Bösartige Neubildungen der Lippe, der Mundhöhle und des Pharynx**	Entzündungen und Neoplasien (benigne,maligne) der Kopfspeicheldrüsen, Sialolithiasis, Benigne und maligne Mundhöhlentumore	21.1.8.45	21.1.7.26	
**C15-C26 Bösartige Neubildungen der Verdauungsorgane**				
C15 Bösartige Neubildung des Ösophagus		21.1.7.11		
C16 Bösartige Neubildung des Magens		21.1.7.16		
C17 Bösartige Neubildung des Dünndarmes		x		
C18 Bösartige Neubildung des Kolons	Benigne,maligne Tumoren des Kolorektums	21.1.7.19		
C19 Bösartige Neubildung am Rektosigmoid, Übergang		21.1.7.19		
C20 Bösartige Neubildung des Rektums		21.1.7.19		
C21 Bösartige Neubildung des Anus und des Analkanals	Analkarzinom	21.1.7.48		
C22 Bösartige Neubildung der Leber und der intahepatischen Gallengänge	Benigne und primäre,sekundäre maligne Tumoren der Leber	21.1.7.21		
C23 Bösartige Neubildung der Gallenblase	Benigne und maligne Tumoren der Gallenblase und der Gallengänge	21.1.7.37		
C24 Bösartige Neubildung sonstiger und nicht näher bezeichneter Teil der Gallenwege		21.1.7.37		
C25 Bösartige Neubildung des Pankreas	Benigne und maligne Tumoren des Pankreas	21.1.7.38		
**C30-C39 Bösartige Neubildungen der Atmungsorgane und sontiger intrathorakealer Organe**				
C32 Bösartige Neubildung des Larynx	malignome des Larynx	21.1.4.18		
C33 Bösartige Neubildung der Trachea		x		
C34 Bösartige Neubildung der Bronchien und der Lunge	Lungenkarzinom und pulmonale Metastasen	21.1.4.17		
**C40-C41 Bösartige Neubildungen des Knochens und des Gelenkknorpels**				

C40 Bösartige Neubildung des Knochens und des Gelenkknorpels der Extremitäten		x		
C41 Bösartige Neubildung des Knochens und des Gelenkknorpels sonstiger und nicht näher bezeichneter Lokalisation		x		
**C43-C44 Melanom und sonstige bösartige Neubildungen der Haut**				
C43 Bösartiges Melanom der Haut	Benigne und maligne Hauttumore	21.1.8.6	20.91	
C44 Sonstige bösartige Neubildungen der Haut		21.1.8.6		
**C45-C49 Bösartige Neubildungen des mesothelialen Gewebes und des Weichteilgewegbes**	Benigne und maligne Weichgewebstumore, Mesotheliom	21.1.2.2	21.1.4.19	
**C50 Bösartige Neubildung der Brustdrüse [Mamma]**	Gutartige und bösartige Tumore der Mamma	21.1.8.5		
**C51-C58 Bösartige Neubildungen der weiblichen Genitalorgane**				
C51 Bösartige Neubildung der Vulva	Vulva- und Peniskarzinom	21.1.6.42		
C52 Bösartige Neubildung der Vagina		x		
C53 Bösartige Neubildung der Cervix uteri	Zervixkarzinom und Dysplasie	21.1.6.26		
C54 Bösartige Neubildung des Corpus uteri	Endometriumkarzinom	21.1.6.24		
C56 Bösartige Neubildung des Ovars	Ovarialkarzinom	21.1.6.25		
C57 Bösartige Neubildung sonstiger und nicht näher bezeichneter weiblicher Genitalorgane	Endometriumkarzinom	21.1.6.24		
**C60-C63 Bösartige Neubildungen der männlichen Genitaloragane**	Prostatakarzinom, Benigne und maligne Hodentumore, Vulva- und Peniskarzinom	21.1.6.42	21.1.6.3	21.1.6.41
**C64-C68 Bösartige Neubildungen der Harnorgane**	Benigne und maligne Tumore der Niere und des harnableitenden Systems	21.1.6.7		
**C69-C72 Bösartige Neubildungen des Auges, des Gehirns und sonstiger Teile des Zentralnervensystems**				
C69 Bösartige Neubildung des Auges und der Augenanhangsgebilde	Retinoblastom	21.1.9.29		
C71 Bösartige Neubildung des Gehirns	Zerebrale und spinale Tumoren und Metastasen	21.1.10.13		
C72 Bösartige Neubildung des Rückenmarkes, der Hirnnerven und anderer Teile des Zentralnervensystems		21.1.10.13		
**C73-C75 Bösartige Neubildungen der Schilddrüse und sonstiger endokriner Drüsen**				
C73 Bösartige Neubildung der Schilddrüse	Struma mit Knoten	21.1.3.1	20.92	
C74 Bösartige Neubildung der Nebenniere	Benigne und maligne Tumoren der Nebenniere	21.1.3.20		
**C76-C80 Bösartige Neubildungen ungenau bezeichneter, sekundärer und nicht näher bezeichneter Lokalisationen**	Lungenkarzinom und pulmonale Metastasen, Zerebrale und spinale Tumoren und Metastasen	21.1.4.17	21.1.10.13	
**C81-C96 Bösartige Neubildungen des lymphatischen, blutbildenden und verwandten Gewebes**		21.1.5.3		
C81 Hodgkin-Krankheit [Lymphogranulomatose]	Lymphome (Hodgkin,Non-Hodgkin)	21.1.5.3		
C82 Follikuläres [noduläres] Non-Hodgkin-Lymphom		21.1.5.3		
C83 Diffuses Non-Hodgkin-Lymphom		21.1.5.3		
C84 Periphere und kutane T-Zell-Lymphome		21.1.5.3		
C90 Plasmozytom und bösartige Plasmazellen-Neubildungen		21.1.5.3		
C91 Lym phatische Leukämie	Leukämie	21.1.5.2		
C92 Myeloische Leukämie		21.1.5.2		
C96 Sonstige und nicht näher bezeichnete bösartige Neibildungen des lymphatischen, blutbildenden und verwandten Gewebes	Neoplasie	21.1.5.13		
**D00-D09 In-situ-Neubildungen**				
DOO Carcinoma in situ der Mundhöhle, des Ösophagus und des Magens		21.1.8.45		
D03 Melanoma in situ		21.1.8.6	20.91	
D04 Carcinoma in situ der Haut		21.1.8.6	20.91	
**D10-D36 Gutartige Neubildungen**				
D12 Gutartige Neubildung des Kolons, des Rektums, des Analkanals und des Anus	Benigne und maligne Tumore des Kolorektums	21.1.7.19		
D13 Gutartige Neubildung sonstiger und ungenau bezeichneter Teile des Verdauungssystems	Benigne und primäre,sekundäre maligne Tumoren der Leber, Entzündungen und Neoplasien der Kopfspeicheldrüsen und Sialolithiasis, benigne und maligne Tumoren der Gallenblase und der Gallengänge, benigne und maligne Tumoren des Pankreas	21.1.7.19, 21.1.7.21	21.1.7.26, 21.1.7.37	21.1.7.38, 21.1.8.45
D14 Gutartige Neubildung des Mittelohres und des Atmungssystems		x		
D16 gutartige Neubildung des Knochens und des Gelenkknorpels		x		
D17 Gutartige Neubildung des Fettgewebes		x		
D18 Hämangiom und Lymphangiom	Angeborene Gefäßmissbildungen (Angiodysplasie, Av-Malformation, Hämangiome, Lymphangiome)	21.1.1.24		

D21 Sonstige gutartige Neubildungen des Bindegewebes und anderer Weichteilgewebe	Benigne und maligne Weichgewebstumore, benigne und maligne Hauttumore	21.1.2.2	21.1.8.6	
D22 Melanozytennävus	Benigne und maligne Hauttumore	21.1.8.6	20.29, 20.91	
D25 Leiomyom des Uterus	Benigne Uterustumore (Myome, Endometriumpolypen, etc.)	21.1.6.18		
D27 Gutartige Neubildung des Ovars		21.1.6.22		
D31 Gutartige Neubildung des Auges und der Augenanhangsgebilde		x		
D32 Gutartige Neubildung der Meningen	Zerebrale und spinale Tumoren und Metastasen	21.1.10.13		
D33 Gutartige Neubildung des Gehirns und anderer Teile des Zentralnervensystems	Zerebrale und spinale Tumoren und Metastasen, Akustikusneurinom, Vestibularisschwannom	21.1.10.13	21.1.10.25	
D35 Gutartige Neubildung sonstiger und nicht näher bezeichneter endokriner Drüsen	Benigne und maligne Tumoren der Nebennieren	21.1.3.26	21.1.3.20	
**D37-D48 Neubildungen unsicheren oder unbekannten Verhaltens**				
D44 Neubildung unsicheren oder unbekannten Verhaltens der endokrinen Drüsen	Benigne und maligne Tumoren der Nebennieren	21.1.3.20		
D45 Polycythaemia vera	Myeloproliferative Neoplasien (Polyzythaemia vera, essentielle Thrombozythämie, Myelofibrose)	21.1.5.13		
D46 Myelodysplastische Syndrome		21.1.5.14		
D47 Sonstige Neubildungen unsicheren oder unbekannten Verhaltens des lympathischen, blutbildenden und verwandten Gewebes	Myeloproliferative Neoplasien (Polyzythaemia vera, essentielle Thrombozythämie, Myelofibrose)	21.1.5.13		
**D50-D53 Alimentäre Anämien**				
D50 Eisenmangelanämie	Anämien	21.1.5.21		
D51 Vitamin-B12-Mangelanämie		21.1.5.21		
D52 Folsäure-Mangelanämie		21.1.5.21		
**D55-D59 Hämolytische Anämien**	Anämien	21.1.5.21		
**D60-D64 Aplastische und sonstige Anämien**	Anämien	21.1.5.21		
**D65-D69 Koagulopathien, Purpura und sonstige hämorrhagische Oiathesen**	Hämophilie A und B, Sonstige hämorrhagische und thrombophile Diathesen und DD Diathesen	21.1.5.10	21.1.5.20	
**D70-D77 Sonstige Krankheiten des Blutes und der blutbildenden Organe**		21.1.5.1-21		
**D80-D90 Bestimmte Störungen mit Beteiligung des Immunsystems**		21.1.5.12		
D83 Variabler Immundefekt [common variable immunodeficiency]		21.1.5.12		
D84 Sonstige Immundefekte	Urtikaria und Angioödem	21.1.5.1-21	21.1.5.12	21.1.8.40
D86 Sarkoidose		21.1.4.20		
D89 Sonstige Störungen mit Beteiligung des Immunsystems, anderernorts nicht klassifiziert		21.1.5.12		
D90 Immunkompromittierung nach Bestrahlung, Chemotherapie und sonstigen immunsuppressiven Maßnahmen		21.1.5.12		
**E00-E07 Krankheiten der Schilddrüse**	Struma mit und ohne Knoten, Hypo- und Hyperthyreose, Immunthyreopathien	21.1.3.1-3		
**E10-E14 Diabetes mellitus**				
E10 Primär insulinabhängiger Diabetes mellitus [Typ-1-Diabetes]	Diabetes mellitus Typ 1	21.1.3.4		
E11 Nicht primär insulinabhängiger Diabetes mellitus [Typ-2-Diabetes]	Diabetes mellitus Typ 2	21.1.3.5		
E14 Nicht näher bezeichneter Diabetes mellitus		21.1.3.17		
**E15-E16 Sonstige Störungen der Blutglukose-Regulation und der inneren Sekretion des Pankreas**		x		
**E20-E35 Krankheiten sonstiger endokriner Orüsen**				
E21 Hyperparathyreoidismus und sonstige Krankheiten der Nebenschilddrüse	Hyper- und Hypoparathyreoidismus	21.1.3.3		
E22 Überfunktion der Hypophyse		13.3.14		
E23 Unterfunktion und andere Störungen der Hypophyse	Diabetes insipidus, Hypophyseninsuffizienz	21.1.3.17	21.1.3.18	
E24 Cushing-Syndrom	Hypercortisolismus (Cushing-Syndrom)	21.1.3.21		
E25 Adrenogenitale Störungen	Adrenogenitales Syndrom	21.1.3.25		
E26 Hyperaldosteronismus		21.1.3.22		
E27 Sonstige Krankheiten der Nebenniere	Nebenniereninsuffizienz (z.B. M. Addison)	21.1.3.23	13.3.14, 21.1.3.20	
E28 Ovarielle Dysfunktion		21.1.6.28	21.1.3.15, 21.1.3.24	
E29 Testikuläre Dysfunktion		x		
E30 Pubertätsstörungen, anderenorts nicht klassifiziert	Pubertas praecox,tarda	21.1.3.10		
E31 Polyglanduläre Dysfunktion		x		

E34 Sonstige endokrine Störungen		13.3.14		
**E40-E46 Mangelernährung**		20.111		
**E50-E64 Sonstige alimentäre Mangelzustände**		16.2.1	20.111	
**E65-E68 Adipositas und sonstige Überernährung**		21.1.2.26	20.43	
E66 Adipositas		21.1.2.26	20.43	
**E70-E90 Stoffwechselstörungen**				
E70 Störungen des Stoffwechsels aromatischer Aminosäuren	Störungen des Aminosäurestoffwechsels	21.1.3.8		
E72 Sonstige Störungen des Aminosäurestoffwechsels		21.1.3.8		
E73 Laktoseintoleranz	Nahrungsmittelunverträglichkeiten und -allergie, Störungen des Kohlenhydratstoffwechsels (Galaktosämie, Glykogenosen)	20.69	21.1.3.9	21.1.7.29
E74 Sonstige Störungen des Kohlenhydratstoffwechsels	Störungen des Kohlenhydratstoffwechsels (Galaktosämie, Glykogenosen)	21.1.3.9		
E75 Störungen des Sphingolipidstoffwechsels und sonstige Störungen der Lipidspeicherung	Störungen des Lipidstoffwechsels	21.1.3.7		
E78 Störungen des Lipoproteinstoffwechsels und sonstige Lipidämien		21.1.3.7		
E79 Störungen des Purin- und Pyrimidinstoffwechsels		21.1.2.3		
E80 Störungen des Porphyrin- und Bilirubinstoffwechsels		21.1.5.11		
E83 Störungen des Mineralstoffwechsels	Elektrolyt, Hyperparathyreoidismus, Morbus Wilson, Hömochromatose	21.1.3.3	21.1.3.19	21.1.3.12, 21.1.3.13
E84 Zystische Fibrose	Mukoviszidose	21.1.4.15	20.10	
E85 Amyloidose		21.1.3.27		
E86 Volumenmangel	Flüssigkeitshaushalt	20.22	20.56, 20.117	21.1.3.19
E87 Sonstige Störungen des Wasser- und Elektrolythaushaltes sowie des Säure-Basen-Gleichgewichts	Azidose und Alkalose, Störungen des Elektrolyt- und Flüssigkeitshaushaltes	21.1.3.14	21.1.3.19	
**F00-F09 Organische, einschließlich symptomatischer psychischer Störugnen**				
F00 Demenz bei Alzheimer-Krankheit	Demenz-Syndrome	21.1.10.59		
F01 Vaskuläre Demenz		21.1.10.59		
F02 Demenz bei anderenorts klassifizierten Krankheiten		21.1.10.59		
F04 Organisches amnestisches Syndrom, nicht durch Alkohol oder andere psychotrope Substanzen bedingt	Amnesieformen, Wenicke-Encephalopathie, organisches amnestisches Syndrom	21.1.10.41	20.32	21.1.10.60
F05 Delir, nicht durch Alkohol oder andere psychotrope Substanzen bedingt	Postoperative kognitive Dysfunktion,Delir	21.1.10.58	21.1.10.42	
F06 Andere psychische Störungen aufgrund einer Schädigung oder Funktionsstörung des Gehirns oder einer körperlichen Krankheit		21.1.10		
F07 Persönlichkeits- und Verhaltensstörung aufgrund einer Krankheit, Schädigung oder Funktionsstörung des Gehirns	Persönlichkeitsstörungen (dissoziale, histionische, paranoide, schizoide, emotional instabile Persönlichkeitsstörung)	21.1.10.61		
**F10-F19 Psychische und Verhaltensstörungen durch psychotrope Substanzen**	Drogen und Medikamentenintoxikation, missbrauch	21.1.10.64	21.1.10.65	
**F20-F29 Schizophrenie, schizotype und wahnhafte Störungen**				
F20 Schizophrenie		21.1.10.66		
F22 Anhaltende wahnhafte Störungen	Wahn	21.1.10.66	20.24	
F23 Akute vorübergehende psychotische Störungen		20.20		
F25 Schizoaffektive Störungen		21.1.10.47		
**F30-F39 Affektive Störungen**				
F31 Bipolare affektive Störung		21.1.10.43		
F32 Depressive Episode	Depression	21.1.10.45		
F33 Rezidivierende depressive Störung		21.1.10.45		
F34 Anhaltende affektive Störungen		21.1.10.45	13.3.2	
**F40-F48 Neurotische, Belastungs- und somatoforme Störungen**				
F40 Phobische Störungen	spezifische Phobien, Angsstörungen	21.1.10.53	21.1.10.54	
F41 Andere Angststörungen	Angststörungen	21.1.10.53		
F42 Zwangsstörung		20.123	21.1.10.57	

F43 Reaktionen auf schwere Belastungen und Anpassungsstörungen	Anpassungsstörungen, Akute Belastungsreaktion und Posttraumatische Belastungsstörung	21.1.10.52	21.1.10.55	
F44 Dissoziative Störungen [Konversionsstörungen]		20.19	20.33, 20.106	13.3.3.(1)
F45 Somatoforme Störungen	Somatisierungsstörung, somatoforme autonome Funktionsstörung, somatoforme Schmerzerkrankung, Hypochondische Störung	21.1.10.56	21.1.10.63	20.85
**F50-F59 Verhaltensauffälligkeiten mit körperlichen Störungen und Faktoren**				
F50 Essstörungen	Anorexia nervosa, Bulimia nervosa, Binge-Eating Störung	20.111	21.1.3.28, 21.1.10.69	21.1.10.70, 21.1.10.71
F51 Nichtorganische Schlafstörungen	Organische und nichtorganische Schlafstörungen	20.83	21.1.10.62	
F52 Sexuelle Funktionsstörungen, nicht verursacht durch eine organische Störung oder Krankheit	erektile Dysfunktion	20.103	21.1.10.39	
F53 Psychische oder Verhaltensstörungen im Wochenbett, anderenorts nicht klassifiziert		21.1.11.10		
F54 Psychologische Faktoren oder Verhaltensfaktoren bei anderenorts klassifizierten Krankheiten		x		
**F60-F69 Persönlichkeits- und Verhaltensstörungen**				
F60 Spezifische Persönlichkeitsstörungen	Persönlichkeitsstörungen (dissoziale, histionische, paranoide, schizoide, emotional instabile Persönlichkeitsstörung)	21.1.10.61		20.73
F68 Andere Persönlichkeits- und Verhaltensstörungen	Persönlichkeitsstörungen (dissoziale, histionische, paranoide, schizoide, emotional instabile Persönlichkeitsstörung), Münchhausen Syndrom, Münchhausen-by-proxy Syndrom	21.1.10.61	21.1.10.74	20.73
**F70-F79 Intelligenzminderung**	Entwicklungsstörungen, Intelligenzminderung	21.1.10.51		
**F80-F89 Entwicklungsstörungen**	Sprech- und Sprachstörungen, Lernstörungen, Entwicklungsstörungen, Intelligenzminderung	21.1.10.48, 21.1.10.49	21.1.10.51	20.119
**F90-F98 Verhaltens- und emotionale Störungen mit Beginn in der Kindheit und Jugend**		20.119	20.26	21.1.10.51
F90 Hyperkinetische Störungen		20.19		
F91 Störungen des Sozialverhaltens		21.1.10.50		
F93 Emotionale Störungen des Kindesalters		20.54		
F94 Störungen sozialer Funktionen mit Beginn in der Kindheit und Jugend	Borderline	20.71		
F95 Ticstörungen		21.1.10.68		
F98 Andere Verhaltens- und emotionale Störungen mit Beginn in der Kindheit und Jugend	Enuresis und Enkopresis	21.1.6.43		
**G00-G09 Entzündliche Krankheiten des Zentralnervensystems**	Meningoenzephalitis	21.1.10.14		
**G10-G13 Systematrophien, die vorwiegend das Zentralnervensystem betreffen**	Multiple Sklerose, AML	21.1.10.11	21.1.10.36	
G10 Chorea Huntington	Neurodegenerative Erkrankungen, z.B. Amyotrophe Lateralsklerose, Chorea Huntington	21.1.10.36		
G11 Hereditäre Ataxie		x		
G12 Spinale Muskelatrophie und verwandte Syndrome	Spinale Muskelatrophie	21.1.10.35		
**G20-G26 Extrapyramidale Krankheiten und Bewegungsstörungen**				
G20 Primäres Parkinson-Syndrom	Parkinson-Syndrom, atypische Parkinson-Syndrome (inkl. Multisystematrophie, Lewy-Körperchen-Demenz)	21.1.10.17	21.1.10.18	
G21 Sekundäres Parkinson-Syndrom		21.1.10.17	21.1.10.18	
G24 Dystonie	Bewegungsstörung	20.19		
G25 Sonstige extrapyramidale Krankheiten und Bewegungsstörungen	Nächtliche Bewegungsstörungen (Restless legs Syndrom)	21.1.10.33		

**G30-G32 Sonstige degenerative Krankheiten des Nervensystems**				
G30 Alzheimer-Krankheit	Neurodegenerative Erkrankungen, z.B. Amyotrophe	21.1.10.36	21.1.10.59	
G31 Sonstige degenerative Krankheiten des Nervensystems, anderenorts nicht klassifiziert		21.1.10.36	21.1.10.59	
**G35-G37 Demyelinisierende Krankheiten des Zentralnervensystems**				
G35 Multiple Sklerose [Encephalomyelitis disseminata]		21.1.10.11		
G36 Sonstige akute disseminierte Demyelinisation		21.1.10.11		
**G40-G47 Episodische und paroxysmale Krankheiten des Nervensystems**				
G40 Epilepsie	Krampfanfälle, Epilepsie, Status epilepticus	21.1.10.19		
G41 Status epilepticus		21.1.10.19		
G43 Migräne		21.1.10.15		
G44 Sonstige Kopfschmerzsyndrome	Spannungskopfschmerz, Clusterkopfschmerz, Medikamenten-induzuerter Kopfschmerz	21.1.10.23	20.57	
G45 Zerebrale transitorische Ischämie und verwandte Syndrome		x		
G46 Zerebrale Gefäßsyndrome bei zerebrovaskulären Krankheiten		x		
G47 Schlafstörungen	Organische und nichtorganische Schlafstörungen	21.1.10.62	20.83	
**G50-G59 Krankheiten von Nerven, Nervenwurzeln und Nervenplexus**				
G50 Krankheiten des N. trigeminus [V. Hirnnerv]	Trigeminusneuralgie und DD atypischer Gesichtsschmerz, Verletzungen und Paresen peripherer Nerven und Hirnnerven	21.1.10.8	21.1.10.21	20.40
G51 Krankheiten des N. facialis [VII. Hirnnerv]	Verletzungen und Paresen peropherer Nerven und Hirnnerven, Fazialisparese	21.1.10.8	21.1.10.24	
G52 Krankheiten sonstiger Hirnnerven	Verletzungen und Paresen peropherer Nerven und Hirnnerven	21.1.10.8		
G54 Krankheiten von Nervenwurzeln und Nervenplexus	Verletzungen und Paresen peropherer Nerven und Hirnnerven	21.1.11.8	21.1.11.15	
G55 Kompression von Nervenwurzeln und Nervenplexus bei anderenorts klassifizierten Krankheiten	Verletzungen und Paresen peropherer Nerven und Hirnnerven, Bandscheibenpathologien und radikuläre Syndrome	21.1.10.5	21.1.10.8	21.1.11.15
G56 Mononeuropathien der oberen Extremität	Periphere Neuropathien	21.1.10.16		
G57 Mononeuropathien der unteren Extremität		21.1.10.16		
**G60-G64 Polyneuropathien und sonstige Krankheiten des peripheren Nervensystems**				
G61 Polyneuritis	Periphere Neuropathien, Guillain-Barré Syndrom und Miller-Fisher-Syndrom	21.1.10.16	21.1.10.28	
G62 Sonstige Polyneuropathien	Periphere Neuropathien	20.106	21.1.3.6	21.1.10.16
G63 Polyneuropathie bei anderenorts klassifizierten Krankheiten	Periphere Neuropathien, Diabeteskomplikationen (Mikro- und Makroangiopathien, Nephropathien, KHK, pAVK, Apoplex, diabetisches Dußsyndrom, diabetische Polyneuropathie, diabetische Retino- und Makulopathie)	20.106	21.1.3.6	21.1.10.16
**G70-G73 Krankheiten im Bereich der neuromuskulären Synapse und des Muskels**				
G70 Myasthenia gravis und sonstige neuromuskuläre Krankheiten		21.1.10.29		
G71 Primäre Myopathien	Muskelschwäche, Muskeldystrophien	20.66	21.1.10.37	

G72 Sonstige Myopathien	Muskelschwäche	20.66		
**G80-G83 Zerebrale Lähmung und sonstige Lähmunssyndrome**				
G80 Infantile Zerebralparese		21.1.10.34		
G81 Hemiparese und Hemiplegie	Lähmung	20.60		
G82 Paraparese und Paraplegie, Tetraparese und Tetraplegie	Lähmung	20.60		
G83 Sonstige Lähmungssyndrome		x		
**G90-G99 Sonstige Krankheiten des Nervensystems**				
G91 Hydrozephalus		21.1.11.39		
G95 Sonstige Krankheiten des Rückenmarkes	Spina	21.1.10.7	21.1.10.13	21.1.11.38
H00-H06 Affektionen des Augenlides, des Tränenappparates und der Orbita				
H00 Hordeolum und Chalazion		21.1.9.14		
H02 Sonstige Affektionen des Augenlides	Ektropium, Entropium, Trichiasis	21.1.9.12		
H04 Affektionen des Tränenapparates	Dakryoadenitis, Dakryozystitis	21.1.9.11		
**H10-H13 Affektionen der Konjunktiva**				
H10 Konjunktivitis		21.1.9.16		
H11 Sonstige Affektionen der Konjunktiva	Hyposphagma	21.1.9.17	21.1.4.23	21.1.9.18
**H15-H22 Affektionen der Sklera, der Hornhaut, der Iris und des Ziliarkörpers**				
H15 Affektionen der Sklera		x		
H16 Keratitis	HSV Keratitis	21.1.9.20	21.1.9.18	
H18 Sonstige Affektionen der Hornhaut	Fremdkörper, Infiltrat der Hornhaut und Erosio, Ulkus Corneae	21.1.9.19	21.1.9.18	
H20 Iridozyklitis	Uveitis: anterior, intermedia, posterior und Endophthalmitis	21.1.9.23		
H22 Affektionen der Iris und des Ziliarkörpers bei anderenorts klassifizierten Krankheiten		21.1.9.23		
**H25-H28 Affektionen der Linse**				
H25 Cataracta senilis	Katarakt	21.1.9.22		
H26 Sonstige Kataraktformen		21.1.9.22		
**H30-H36 Affektionen der Aderhaut und der Netzhaut**				
H30 Chorioretinitis	Uveitis: anterior, intermedia, posterior und Endophthalmitis	21.1.9.23		
H32 Chorioretinale Affektionen bei anderenorts klassifizierten Krankheiten		x		
H33 Netzhautablösung und Netzhautriss	Ablatio retinae	21.1.9.24		
H34 Netzhautgefäßverschluss	Gefäßverschlüsse der Netzhaut	21.1.9.25		
H35 Sonstige Affektionen der Netzhaut	Hypertone Veränderungen der Netzhaut, Altersbedingte Makuladegeneration	21.1.9.26	21.1.9.27	
H36 Affektionen der Netzhaut bei anderenorts klassifizierten Krankheiten	Diabeteskomplikationen (Mikro- und Makroangiopathien, Nephropathie, KHK, pAVK, Apoplex, diabetisches Fußsyndrom, diabetisches Polyneuropathie, diabetische Retino- und Makulopathie)	21.1.9.26	21.1.3.6	
**H40-H42 Glaukom**		21.1.9.21		
**H43-H45 Affektionen des Glaskörpers und des Augapfels**				
H43 Affektionen des Glaskörpers		x		
H44 Affektionen des Augapfels	Bulbusverletzungen: Contusio, Perforation, Fremdkörper, Uveitis: anterior, intermedia, posterior und Endophthalmitis	21.1.9.15	21.1.9.23	
**H46-H48 Affektionen des N. opticus und der Sehbahn**				
H46 Neuritis nervi optici	Stauungspapille, Atrophie des N. opticus	21.1.9.28		
H47 Sonstige Affektionen des N. opticus [11. Hirnnerv] und der Sehbahn		21.1.9.28		
H48 Affektionen des N. opticus [11. Hirnnerv] und der Sehban bei anderenorts klassifizierten Krankheiten		21.1.9.28		

H49-H52 Affektionen der Augenmuskeln, Störungen der Blickbewegungen und Refraktionsfehler		x		
H49 Strabismus paralyticus		20.25		
H50 Sonstiger Strabismus		20.25		
H52 Akkommodationsstörungen und Refraktionsfehler		21.1.9.9		
**H53-H54 Sehstörungen und Blindheit**		20.25		
**H55-H59 Sonstige Affektionen des Auges und der Augenanhangsgebilde**		21.1.9.9-29		
H57 Sonstige Affektionen des Auges und der Augenanhangsgebilde		21.1.9.9-29		
**H60-H62 Krankheiten des äußeren Ohres**				
H60 Otitis externa	Otitis exerna einschl. Otitis externa maligna	21.1.9.1		
H61 Sonstige Krankheiten des äußeren Ohres		x		
**H65-H75 Krankheiten des Mittelohres und des Warzenfortsatzes**				
H65 Nichteitrige Otitis media	Otitis media acuta und chronica einschl. otogener Komplikationen	21.1.9.7		
H66 Eitrige und nicht näher bezeichnete Otitis media		21.1.9.7		
H68 Entzündung und Verschluss der Tuba auditiva	Tubenfunktionsstörung	21.1.9.2		
H70 Mastoiditis und verwandte Zustände		x		
H71 Cholesteatom des Mittelohres		x		
H72 Trommelfellperforation	Trommelfellverletzungen	21.1.9.4		
**H80-H83 Krankheiten des Innenohres**				
H80 Otosklerose		21.1.9.8		
H81 Störungen der Vestibularfunktion	Peripher-vestibuläre Schwindelformen (BPLS, M. Menière, Neuritis vestibularis)	21.1.10.27		
H83 Sonstige Krankheiten des Innenohres	Schallempfindungsstörung	21.1.9.3		
**H90-H95 Sonstige Krankheiten des Ohres**				
H90 Hörverlust durch Schalleitungs- oder Schallempfindungsstörung	Schallleitungsschwerhörigkeit (z.B. Tubenfunktionsstörung, Paikenerguß)	21.1.9.3	21.1.9.2	
H91 Sonstiger Hörverlust		21.1.9.5		
H93.1 Tinnitus aurium	Ohrgeräusche, Tinitus	21.1.9.6		
**100-102 Akutes rheumatisches Fieber**	Rheumatisches Fieber	21.1.5.9		
**105-109 Chronische rheumatische Herzkrankheiten**				
105 Rheumatische Mitralklappenkrankheiten	Klappenerkrankung	21.1.1.8		
106 Rheumatische Aortenklappenkrankheiten	Klappenerkrankung	21.1.1.8		
107 Rheumatische Trikuspidalklappenkrankheiten	Klappenerkrankung	21.1.1.8		
**110-115 Hypertonie [Hochdruckkrankheit]**				
110 Essentielle (primäre) Hypertonie		21.1.1.17		
111 Hypertensive Herzkrankheit	Cor pulmonale	21.1.1.20		
112 Hypertensive Nierenkrankheit	Nierenarterienstenose	21.1.128		
115 Sekundäre Hypertonie		21.1.1.19		
**120-125 Ischämische Herzkrankheiten**	Diabeteskomplikationen (Mikro- und Makroangiopathien, Nephropathie, KHK, pAVK, Apoplex, diabetisches Fußsyndrom, diabetisches Polyneuropathie, diabetische Retino- und Makulopathie)	21.1.3.6		
120 Angina pectoris	Thoraxschmerz, akutes Koronarsyndrom	20.107	21.1.1.15	
121 Akuter Myokardinfarkt	Akutes Koronarsyndrom	20.7	21.1.1.15	
122 Rezidivierender Myokardinfarkt	Akutes Koronarsyndrom	21.1.1.15		
125 Chronische ischämische Herzkrankheit	Kronare Herzkrankheit	21.1.1.12		
**126-128 Pulmonale Herzkrankheit und Krankheiten des Lungenkreislaufes**				
126 Lungenembolie		21.1.4.3		
127 Sonstige pulmonale Herzkrankheiten	Cor pulmonale	21.1.1.20		
**130-152 Sonstige Formen der Herzkrankheit**				
130 Akute Perikarditis		21.1.1.11		
131 Sonstige Krankheiten des Perikards	Herzbeuteltamponade	21.1.1.23		
133 Akute und subakute Endokarditis	Endokarditis	21.1.1.9		
134 Nichtrheumatische Mitralklappenkrankheiten	Herzklappenerkrankung	21.1.1.8		
135 Nichtrheumatische Aortenklappenkrankheiten		21.1.1.8		
136 Nichtrheumatische Trikuspidalklappenkrankheiten		21.1.1.8		
138 Endokarditis, Herzklappe nicht näher bezeichnet	Endokarditis	21.1.1.9		
139 Endokarditis und Herzklappenkrankheiten bei Anderenorts klassifizierten Krankheiten		21.1.1.9		
140 Akute Myokarditis	Myokarditis	21.1.1.10		

141 Myokarditis bei anderenorts klassifizierten Krankheiten		21.1.1.10		
142 Kardiomyopathie		21.1.1.25		
144 Atrioventrikulärer Block und Linksschenkelblock	Herzrhythmusstörungen	21.1.1.13		
145 Sonstige kardiale Erregungsleitungsstörungen		21.1.1.13		
146 Herzstillstand		21.1.1.13		
147 Paroxysmale Tachykardie		21.1.1.13		
148 Vorhofflattern und Vorhofflimmern		21.1.1.13		
149 Sonstige kardiale Arrhythmien		21.1.1.13		
150 Herzinsuffizienz		21.1.1.14		
**160-169 Zerebrovaskuläre Krankheiten**				
160 Subarachnoidalblutung	intrakranielle Blutung	21.1.10.4		
161 Intrazerebrale Blutung		21.1.10.4		
162 Sonstige nichttraumatische intrakranielle Blutung		21.1.10.4		
163 Hirninfarkt	Diabeteskomplikationen (Mikro- und Makroangiopathien, Nephropathie, KHK, pAVK, Apoplex, diabetisches Fußsyndrom, diabetisches Polyneuropathie, diabetische Retino- und Makulopathie)	21.1.3.6		
165 Verschluss und Stenose präzerebraler Arterien ohne resultierenden Hirninfarkt	Gefäßverschlüsse	x		
166 Verschluss und Stenose zerebraler Arterien ohne resultierenden Hirninfarkt	Zerebrovaskuläre Verschlusskrankheit (cAVK)	21.1.10.1		
167 Sonstige zerebrovaskuläre Krankheiten		21.1.10.1		
169 Folgen einer zerebrovaskulären Krankheit		21.1.10.1		
**170-179 Krankheiten der Arterien, Arteriolen und Kapillaren**				
170 Atherosklerose		21.1.1.26		
171 Aortenaneurysma und -dissektion	Aneurysma, Aortendissektion	21.1.1.5	21.1.1.6	
172 Sonstiges Aneurysma	Aneurysma	21.1.1.5		
173 Sonstige periphere Gefäßkrankheiten	Peripher arterielle Verschlusskrankheit(pAVK), Primäres und sekundäres Raynaud-Syndrom, Diabeteskomplikationen (Mikro- und Makroangiopathien, Nephropathie, KHK, pAVK, Appoplex, diabetisches Fußsyndrom, diabetische Polyneuropathie, diabetische Retino- und Makulapathie)	21.1.1.4	21.1.1.27	21.1.3.6
174 Arterielle Embolie und Thrombose	Akute arterielle Thrombose und Embolie der Extremitäten inkl. Cholesterinembolie-Syndrom	21.1.1.3		
**180-189 Krankheiten der Venen, der Lymphgefäße und der Lymphknoten, anderenorts nicht klassifiziert**				
180 Phlebitis und Thrombophlebitis	Thrombophlebitis und Phlebothrombose, chronisch venöse nsuffizienz	21.1.1.7		
181 Pfortaderthrombose		x		
182 Sonstige venöse Embolie und Thrombose		21.1.1.7		
183 Varizen der unteren Extremitäten	Ulcus cruris, Varikosis	21.1.8.2	21.1.8.3	
184 Hämorrhoiden		21.1.7.20	20.77	
185 Ösophagusvarizen		x		
186 Varizen sonstiger Lokalisationen	Varikozele, Hydrozele und Spermatozele	21.1.6.6		
187 Sonstige Venenkrankheiten		21.1.1.7		
**195-199 Sonstige und nicht näher bezeichnete Krankheiten des Kreislaufsystems**				
195 Hypotonie		x		
**J00-J06 Akute Infektionen der oberen Atemwege**				
J00 Akute Rhinopharyngitis [Erkältungsschnupfen]	Akute und chronische Rhinitis und Sinusitis (inkl. Dentogener Sinusitis),	21.1.4.22		
J01 Akute Sinusitis		21.1.4.22	21.1.4.23	
J02 Akute Pharyngitis		21.1.4.22	20.44, 20.84	

J03 Akute Tonsillitis	Tonsillitis, Peritonsillarabszess und Retropharyngealabszess und sonstige Logenabszesse	20.1.5.5	20.44		
J04 Akute Laryngitis und Tracheitis	Stenosierende Laryngotracheitis (Pseudokrupp, Laryngitis subglottica) und sonstige akute und chronische Entzündungen des Kehlkopfs	21.1.4.26	20.44		
J05 Akute obstruktive Laryngitis [Krupp] und Epiglottitis	Epiglottitis, Stenosierende Laryngotracheitis (Pseudokrupp, Laryngitis subglottica) und sonstige akute und chronische Entzündungen des Kehlkopfs	21.1.4.21	21.1.4.26		
J06 Akute Infektionen an mehreren oder nicht näher bezeichneten Lokalisationen der oberen Atemwege	Influenza	21.1.4.10			
**J09-J18 Grippe und Pneumonie**		20.27	21.1.4.9	21.1.4.10	
**J20-J22 Sonstige akute Infektionen der unteren Atemwege**					
J20 Akute Bronchitis	Akute und chronische Bronchitis, Bronchiektasien	21.1.4.6			
J21 Akute Bronchiolitis		21.1.4.7			
**J30-J39 Sonstige Krankheiten der oberen Atemwege**					
J30 Vasomotorische und allergische Rhinopathie	Allergische Rhinokonjunktivitis und allergische Rhinosinusitis	21.1.4.23			
J31 Chronische Rhinitis, Rhinopharyngitis und Pharyngitis	Akute und chronische Rhinitis und Sinusitis (inkl. Dentogener Sinusitis),	21.1.4.22			
J32 Chronische Sinusitis		21.1.4.22			
J33 Nasenpolyp	Hypertrophie der Polypen und Mandeln	20.16	21.1.5.6		
J34 Sonstige Krankheiten der Nase und der Nasennebenhöhlen	Epitaxis	20.14	21.1.4.24		
J35 Chronische Krankheiten der Gaumen- und Rachenmandeln	Tonsillitis, Peritonsillarabszess , Retropharyngealabszess und sonstige Logenabszesse, Hypertrophie der Polypen und Mandeln	21.1.5.5	21.1.5.6		
J38 Krankheiten der Stimmlippen und des Kehlkopfes, anderenorts nicht klassifiziert	Stenosierende Laryngotracheitis (Pseudokrupp, Laryngitis subglottica) und sonstige akute und chronische Entzündungen des Kehlkopfs	21.1.4.26			
**J40-J47 Chronische Krankheiten der unteren Atemwege**					
J41 Einfache und schleimig-eitrige chronische Bronchitis	Akute und Chronische Bronchitis, Bronchiektasien	20.49	21.1.4.6		
J43 Emphysem	Lungenemphysem	21.1.4.11			
J44 Sonstige chronische obstruktive Lungenkrankheit		21.1.4.8			
J45 Asthma bronchiale		21.1.4.13			
J46 Status asthmaticus		x			
J47 Bronchiektasen	Akute und chronische Bronchitis, Bronchiektasien	21.1.4.6			
**J60-J70 Lungenkrankheiten durch exogene Substanzen**					
J61 Pneumokoniose durch Asbest und sonstige anorganische Fasern	Pneumokoniosen	21.1.4.33			
J62 Pneumokoniose durch Quarzstaub		21.1.4.33			
J63 Pneumokoniose durch sonstige anorganische Stäube		21.1.4.33			
J66 Krankheit der Atemwege durch spezifischen organischen Staub		x			
J67 Allergische Alveolitis durch organischen Staub		x			
J69 Pneumonie durch feste und flüssige Substanzen		21.1.4.9			
**J80-J84 Sonstige Krankheiten der Atmungsorgane, die hauptsächlich das Interstitium betreffen**					
J81 Lungenödem		x			
J84 Sonstige interstitielle Lungenkrankheiten	Lungenfibrose	21.1.4.29			
**J85-J86 Purulente und nekrotisierende Krankheitszustände der unteren Atemwege**					
J86 Pyothorax	Pleuraerguss, Pleuritis, Pleuraempyem, Hämatothorax, Chylothorax	21.1.4.5			
**J90-J94 Sonstige Krankheiten der Pleura**					
J90 Pleuraerguss, anderenorts nicht klassifiziert	Pleuraerguss, Pleuritis, Pleuraempyem,	21.1.4.5			
J91 Pleuraerguss bei anderenorts klassifizierten Krankheiten		21.1.4.5			
J93 Pneumothorax		21.1.4.4			

**J95-J99 Sonstige Krankheiten des Atmungssystems**				
J98 Sonstige Krankheiten der Atemwege	Lungenemphysem, Mediastinitis	21.1.4.11	21.1.4.12	
**K00-K14 Krankheiten der Mundhöhle, der Speicheldrüsen und der Kiefer**	Entzündungen und Neoplasien (benigne,maligne) der Kopfspeicheldrüsen, Sialolithiasis	21.1.8.45	21.1.7.26	
K10 Sonstige Krankheiten der Kiefer		20.88		
K12 Stomatitis und verwandte Krankheiten		20.88	20.120	
K13 Sonstige Krankheiten der Lippe und der Mundschleimhaut		20.120		
**K20-K31 Krankheiten des Ösophagus, des Magens und des Duodenums**				
K20 Ösophagitis	Refluxösophagitis, Barrett-Ösophagus	21.1.7.10	20.107	
K21 Gastroösophageale Refluxkrankheit		21.1.7.10		
K22 Sonstige Krankheiten des Ösophagus	Achalasie, Divertikel, Pseudodiverikel des Ösophagus und Pharynx	21.1.7.10, 21.1.7.11	21.1.7.39, 21.1.7.40	
K25 Ulcus ventriculi	Gastroduodenale Ulkuserkrankung	21.1.7.15		
K26 Ulcus duodeni		21.1.7.15		
K29 Gastritis und Duodenitis	akute und chronische Gastritis, Kolitis, Enterokolitis, Gastroenteritis und Wurmerkrankungen	21.1.7.25	21.1.7.28	
K30 Dyspepsie		x		
K31 Sonstige Krankheiten des Magens und des Duodenums		x		
**K35-K38 Krankheiten der Appendix**		20.15	20.110, 21.1.7.1	
K35 Akute Appendizitis		21.1.7.1		
**K40-K46 Hernien**		21.1.7.12	21.1.7.13, 21.1.7.14	21.1.11.33
K40 Hernia inguinalis	Leistenhernie und Femoralhernie	21.1.7.12		
K41 Hernia femoralis		21.1.7.12		
K42 Hernia umbilicalis	Bauchwandhernie, Nabelhernie, Nabelschnurhernie und [illegible]	21.1.7.14		
K43 Hernia ventralis		21.1.7.14		
K44 Hernia diaphragmatica	Diaphragmale Hernie	21.1.11.33	21.1.7.13	
**K50-K52 Nichtinfektiöse Enteritis und Kolitis**	Kolitis, Enterokolitis, Gastroenteritis und Wurmerkrankungen	20.15, 20.113	21.1.7.18	21.1.7.28
K50 Crohn-Krankheit [Enteritis regionalis] [Morbus Crahn]	Chronisch entzündliche Darmerkrankungen	21.1.7.18		
K51 Colitis ulcerasa		21.1.7.18		
**K55-K63 Sonstige Krankheiten des Darmes**				
K55 Gefäßkrankheiten des Darmes	Verschlusskrankheit der Viszeralarterien (Mesenterialischämie)	21.1.7.22		
K56 Paralytischer Ileus und mechanischer Ileus ohne Hernie	Mechanischer und paralytischer Ileus, Invagination	21.1.7.4	21.1.7.32	
K57 Divertikulose des Darmes	Dünndarm- und Dickdarmdivertikel inkl. Komplikationen	21.1.7.17		
K58 Reizdarmsyndrom		21.1.7.45		
K59 Sonstige funktionelle Darmstörungen	Obstipation	21.1.7.41		
K60 Fissur und Fistel in der Anal- und Rektalregion	Analfissur, Analfistel und Pilonidalsinus	21.1.7.35		
K61 Abszess in der Anal- und Rektalregion		x		
K62 Sonstige Krankheiten des Anus und des Rektums		21.1.7.35	21.1.7.48	
K63 Sonstige Krankheiten des Darmes	Kolitis, Enterokolitis, Gastroenteritis und Wurmerkrankungen	21.1.7.28		
**K65-K67 Krankheiten des Peritoneums**				
K65 Peritonitis	Peritonitis, Akutes Abdomen	21.1.7.5		
**K70-K77 Krankheiten der Leber**		20.34	21.1.7.8, 21.1.7.21	21.1.7.24, 21.1.7.46
K70 Alkoholische Leberkrankheit	Alkoholische und nicht-alkoholische	21.1.7.24	21.1.7.30	
K71 Toxische Leberkrankheit		21.1.7.24		
K72 Leberversagen, anderenorts nicht klassifiziert		x		
K74 Fibrose und Zirrhose der Leber	Leberzirrhose	21.1.7.24		
K75 Sonstige entzündliche Leberkrankheiten	Hepatitis, Autoimmunogene Hepatitiden, alkoholische und nicht-alkoholische Steatohepatitis	21.1.7.23	21.1.7.44, 21.1.7.43	21.1.7.30
K76 Sonstige Krankheiten der Leber	Fettlebererkrankung	21.1.7.46		

**K80-K87 Krankheiten der Gallenblase, der Gallenwege und des Pankreas**				
K80 Cholelithiasis		21.1.7.3		
K81 Cholezystitis		21.1.7.2		
K83 Sonstige Krankheiten der Gallenwege		21.1.7.37		
K85 Akute Pankreatitis		21.1.7.6		
K86 Sonstige Krankheiten des Pankreas		21.1.7.6-8		
**K90-K93 Sonstige Krankheiten des Verdauungssystems**				
K90 Intestinale Malabsorption	Zöliakie	20.42	20.111	21.1.7.27
K92 Sonstige Krankheiten des Verdauungssystems		21.1.7		
**L00-L08 Infektionen der Haut und der Unterhaut**				
L00 Staphylococcal scalded skin syndrome [SSS-Syndrom]		21.1.8.18		
L01 Impetigo	Impetigo contagiosa	21.1.8.41		
L02 Hautabszess, Furunkel und Karbunkel	Entzündungen der Haut und Hautanhangsgebilde (z.B. Erysipel, Phlegmone,	21.1.8.42		
L03 Phlegmone		21.1.8.42		
L04 Akute Lymphadenitis		21.1.5.1		
L05 Pilonidalzyste		21.1.7.35		
L08 Sonstige lokale Infektionen der Haut und der Unterhaut	Entzündungen der Haut und Hautanhangebilde (z.B. Erysipel, Phlegmone, Follikultitis, Furunkel)	21.1.8.42		
**L10-L14 Bullöse Dermatosen**				
L10 Pemphiguskrankheiten		20.21		
L12 Pemphigoidkrankheiten		20.21		
L13 Sonstige bullöse Dermatosen		20.21		
**L20-L30 Dermatitis und Ekzem**				
L20 Atopisches [endogenes] Ekzem		21.1.8.21		
L21 Seborrhoisches Ekzem		21.1.8.25		
L22 Windeldermatitis		21.1.8.35		
L23 Allergische Kontaktdermatitis	Toxische und allergische Kontaktdermatitis	21.1.8.10		
L24 Toxische Kontaktdermatitis		21.1.8.10		
L27 Dermatitis durch oral, enteral oder parenteral aufgenommene Substanzen	Arzneimittel-Exanthem	21.1.8.13	21.1.8.17	
L30 Sonstige Dermatitis		21.1.8.10	21.1.8.13	21.1.8.14
**L40-L45 Papulosquamöse Hautkrankheiten**				
L40 Psoriasis		21.1.8.24	20.89, 20.1.12	
L41 Parapsoriasis		x		
L42 Pityriasis rosea		x		
L43 Lichen ruber planus		x		
**L50-L54 Urtikaria und Erythem**				
L50 Urtikaria	Urtikaria und Angioödem	21.1.8.40		
L51 Erythema exsudativum multiforme	Toxische epidermale Nekrolyse, Stevens-Johnson-Syndrom	21.1.8.19		
L52 Erythema nodosum		x		
**L55-L59 Krankheiten der Haut und der Unterhaut durch Strahleneinwirkung**				
L55 Dermatitis solaris acuta		21.1.8.14		
L56 Sonstige akute Hautveränderungen durch Ultraviolettstrahlen		x		
L57 Hautveränderungen durch chronische Exposition gegenüber nichtionisierender Strahlung		x		
**L60-L75 Krankheiten der Hautanhangsgebilde**				
L60 Krankheiten der Nägel	Erkrankungen des Nagels, Fingerkuppenverletzungen	20.112	21.1.8.8	
L63 Alopecia areata	Effluvium und Alopezie	21.1.8.43		
L64 Alopecia androgenetica		21.1.8.43		
L68 Hypertrichose	Veränderungen der Haare	20.112		
L70 Akne		21.1.8.30		
L71 Rosazea		21.1.8.15		
L72 Follikuläre Zysten der Haut und der Unterhaut		x		
L73 Sonstige Krankheiten der Haarfollikel		x		
**L80-L99 Sonstige Krankheiten der Haut und der Unterhaut**				
L80 Vitiligo		21.1.8.28		
L82 Seborrhoische Keratose		21.1.8.26		
L83 Acanthosis nigricans		x		
L85 Sonstige Epidermisverdickung		x		
L88 Pyoderma gangraenosum		20.120	20.91	
L89 Dekubitalgeschwür	Dekubitus	21.1.8.4		
L90 Atrophische Hautkrankheiten		x		
L92 Granulomatöse Krankheiten der Haut und der Unterhaut		x		
L93 Lupus erythematodes		21.1.8.22		
L94 Sonstige lokalisierte Krankheiten des Bindegewebes		x		
**M00-M03 Infektiöse Arthropathien**				
M00 Eitrige Arthritis		21.1.2.41	20.86	
M01 Direkte Gelenkinfektionen bei anderenorts Lymeklassifizierten infektiösen und parasitären Krankheiten		20.35	20.86	21.1.2.33
M02 Reaktive Arthritiden	Septische Arthritis, reaktive Arthritis, 'Coxitis fugax	21.1.2.33	21.1.2.47	

M03 Postinfektiöse und Reaktive Arthritiden bei anderenorts klassifizierten Krankheiten		21.1.2.33		
**M05-M14 Entzündliche Polyarthropathien**	Gicht und Chondrokalzinose, Seropositive und seronegative rheumatoide Arthritis, Psoriasis, Psoriasisarthritis	21.1.2.3	21.1.2.38	21.1.8.24
**M15-M19 Arthrose**	Arthrose	21.1.2.31	20.35	20.86
M15 Polyarthrose		21.1.2.31		
M16 Koxarthrose [Arthrose des Hüftgelenkes]		21.1.2.31		
M17 Gonarthrose [Arthrose des Kniegelenkes]		21.1.2.31		
M19 Sonstige Arthrose		21.1.2.31		
**M20-M25 Sonstige Gelenkkrankheiten**		20.35	20.86	
M20 Erworbene Deformitäten der Finger und Zehen	Hallux valgus	21.1.11.23	21.1.2.27	
M21 Sonstige erworbene Deformitäten der Extremitäten		x		
M22 Krankheiten der Patella	Verletzung der Patella (Patellafraktur, -luxation)	21.1.2.32		
M23 Binnenschädigung des Kniegelenkes [internal derangement]	Traumatische und degenerative Kniebinnenschäden (Meniskusläsion, Kreuzbandruptur)	21.1.2.22		
M24 Sonstige näher bezeichnete Gelenkschädigungen		21.1.2.16, 21.1.2.18	21.1.2.24, 21.1.2.36	
M25 Sonstige Gelenkkrankheiten, anderenorts nicht klassifiziert	Schulterluxation, Ellenbogenluxation, Eadiuskopfsubluxation und weitere Gelenkluxationen, Sonstige Arthritifen und DD Arthritis	21.1.2.16 21.1.2.18	21.1.2.24, 21.1.2.36	21.1.2.41
**M30-M36 Systemkrankheiten des Bindegewebes**				
M30 Panarteriitis nodosa und verwandte Zustände	Kawasaki-Syndrom (mukokutanes Lymphknotensyndrom), Vaskulitis	21.1.8.33	21.1.1.23	
M31 Sonstige nekrotisierende Vaskulopathien	Vaskulitis	21.1.1.23		
M32 Systemischer Lupus erythematodes		21.1.8.22		
M33 Dermatomyositis- Polymyositis		21.1.2.29		
M34 Systemische Sklerose		21.1.8.44		
M35 Sonstige Krankheiten mit System beteiligung des Bindegewebes		x		
**M40-M43 Deformitäten der Wirbelsäule und des Rückens**				
M40 Kyphose und Lordose	Skoliosen und sonstige Wirbelsäulendeformitäten	21.1.2.13		
M41 Skoliose		21.1.2.13		
M42 Osteochondrose der Wirbelsäule		21.1.2.13	21.1.2.11	
M43 Sonstige Deformitäten der Wirbelsäule und des Rückens		21.1.2.13		
**M45-M49 Spondylopathien**				
M45 Spondylitis ankylosans	Morbus Bechterew, Osteochrondrosis und Spondylose	21.1.2.39, 21.1.2.11		
M46 Sonstige entzündliche Spondylopathien		x		
M47 Spondylose	Osteochrondrosis und Spondylose	21.1.2.11		
M48 Sonstige Spondylopathien		x		
**M50-M54 Sonstige Krankheiten der Wirbelsäule und des Rückens**				
M50 Zervikale Bandscheibenschäden	Bandscheibenpathologien und radikuläre Syndrome	21.1.10.5		
M51 Sonstige Bandscheibenschäden		21.1.10.5		
M53 Sonstige Krankheiten der Wirbelsäule und des Rückens, anderenorts nicht klassifiziert		x		
M54 Rückenschmerzen	chronische Rückenschmerzen	20.82	21.1.2.44	
**M60-M63 Krankheiten der Muskeln**				
M60 Myositis		20.66	21.1.2.29	
M61 Kalzifikation und Ossifikation von Muskeln	Muskeldystrophie	21.1.10.37		
**M65-M68 Krankheiten der Synovialis und der Sehnen**	Achillessehnenruptur und Sehnenrupturen an anderen Lokalisationen	21.1.2.25		
M65 Synovitis und Tenosynovitis	Sehnenscheiden-entzündung	21.1.2.9		
**M70-M79 Sonstige Krankheiten des Weichteilgewebes**				
M70 Krankheiten des Weichteilgewebes im Zusammenhang mit Beanspruchung, Überbeanspruchung und Druck		21.1.2		
M71 Sonstige Bursopathien		x		
M72 Fibromatosen	Schwerwiegende Weichgewebsinfektionen (z.B. Nekrotisierende Fasziitis)	21.1.2.7	21.1.10.38	
M75 Schulterläsionen	Verletzung und degenerative Veränderungen der Rotatorenmanschette	21.1.11.13	21.1.2.18	21.1.2.18
M76 Enthesopathien der unteren Extremität mit Ausnahme des Fußes	Sehnenruptur	21.1.2.25		

M77 Sonstige Enthesopathien	Sehnenscheiden-entzündung	21.1.2.9		
M79 Sonstige Krankheiten des Weichteilgewebes, anderenorts nicht klassifiziert	Schwerwiegende Weichgewebsinfektionen (z.B. Nekrotisierende Fasziitis), Fibromyalgie-Syndrom, Lipödem	21.1.2.7	21.1.2.42	21.1.2.43
**M80-M85 Veränderungen der Knochendichte und -struktur**				
M80 Osteoporose mit pathologischer Fraktur	Osteoporose	21.1.2.10		
M81 Osteoporose ohne pathologische Fraktur		21.1.2.10		
M84 Veränderungen der Knochenkontinuität		x		
M85 Sonstige Veränderungen der Knochendichte und -struktur	Osteomalazie und Rachitis	21.1.2.10	21.1.2.11	21.1.2.37
**M86-M90 Sonstige Osteopathien**				
M86 Osteomyelitis		21.1.2.12		
M87 Knochennekrose	Aseptische Knochennekrosen (Osgood-Schlatter, Perthes, Knienböck,…)	21.1.2.34		
M88 Osteodystrophia deformans [Paget-Krankheit]		x		
M89 Sonstige Knochenkrankheiten	Komplexes regionales Schmerzsyndrom	21.1.2	21.1.10.22	
**M91-M94 Chondropathien**	Osteochondrosis und Spondylose	21.1.2.3	21.1.2.11	21.1.2.46
**M95-M99 Sonstige Krankheiten des Muskel-Skelett-Systems und des Bindegewebes**		21.1.2		
M99 Biomechanische Funktionsstörungen, anderenorts nicht klassifiziert	Spinalkanalstenose (zervikal, lumbal)	21.1.10.7		
**N00-N08 Glomeruläre Krankheiten**				
N00 Akutes nephritisches Syndrom	Glomerulonephritiden	21.1.6.12		
N01 Rapid-progressives nephritisches Syndrom		21.1.6.12		
N02 Rezidivierende und persistierende Hämaturie		20.45	21.1.6.12	
N03 Chronisches nephritisches Syndrom		21.1.6.12		
N04 Nephrotisches Syndrom		21.1.6.12		
**N10-N16 Tubulointerstitielle Nierenkrankheiten**				
N10 Akute tubulointerstitielle Nephritis	Tubulo-interstitielle Nierenerkrankungen	21.1.6.13		
N11 Chronische tubulointerstitielle Nephritis		21.1.6.13		
N13 Obstruktive Uropathie und Refluxuropathie		21.1.6.13		
N14 Arzneimittel- und schwermetallinduzierte tubulointerstitielle und tubuläre Krankheitszustände		21.1.6.13		
N15 Sonstige tubulointerstitielle Nierenkrankheiten		21.1.6.13		
**N17-N19 Niereninsuffizienz**				
N17 Akutes Nierenversagen		21.1.6.14		
N18 Chronische Niereninsuffizienz		21.1.6.15		
**N20-N23 Urolithiasis**				
N20 Nieren- und Ureterstein	Urolithiasis und Nierenkoliken	21.1.6.5		
N21 Stein in den unteren Harnwegen		21.1.6.5		
**N25-N29 Sonstige Krankheiten der Niere und des Ureters**				
N25 Krankheiten infolge Schädigung der tubulären Nierenfunktion		x		
N26 Schrumpfniere, nicht näher bezeichnet		x		
N28 Sonstige Krankheiten der Niere und des Ureters, anderenorts nicht klassifiziert	Diabeteskomplikationen (Mikro- und Makroangiopathien, Nephropathie, KHK, pAVK, Apoplex, diabetisches Fußsyndrom, diabetisches Polyneuropathie, diabetische Retino- und Makulopathie)	21.1.6		
**N30-N39 Sonstige Krankheiten des Harnsystems**				
N30 Zystitis	Cystitis	20.45	21.1.6.1	
N31 Neuromuskuläre Dysfunktion der Harnblase, anderenorts nicht klassifiziert	Blasenfunktionsstörungen	21.1.6.31		
N32 Sonstige Krankheiten der Harnblase		21.1.6.31		
N34 Urethritis und urethrales Syndrom	Infektionen der Niere und der ableitenden Harnwege (Pyelonephritis, Cystitis, Urethritis)	21.1.6.1		
N35 Harnröhrenstriktur	Harnröhrenstriktur, Ureterstriktur, Hydronephrose	21.1.6.11		
N39 Sonstige Krankheiten des Harnsystems		21.1.6		
**N40-N51 Krankheiten der männlichen Genitalorgane**				
N40 Prostatahyperplasie	Benignes Prostatahyperplasie Syndrom	21.1.6.2		
N41 Entzündliche Krankheiten der Prostata		21.1.6.4		
N43 Hydrozele und Spermatozele	Varikozele, Hydrozele und Spermatozele	21.1.6.6		
N44 Hodentorsion und Hydatidentorsion	akutes Skrotum (Orchitis,	21.1.6.9		

N45 Orchitis und Epididymitis	Epididymitis,	21.1.6.9		
N46 Sterilität beim Mann	Sterilität und Infertilität	21.1.6.38		
N47 Vorhauthypertrophie, Phimose und Paraphimose	Phimose, Paraphimose	21.1.6.10		
N48 Sonstige Krankheiten des Penis	Balanitis, Priapismus	21.1.6.36	21.1.6.40	
N49 Entzündliche Krankheiten der männlichen Genitalorgane, anderenorts nicht klassifiziert		x		
**N60-N64 Krankheiten der Mamma [Brustdrüse]**				
N60 Gutartige Mammadysplasie	Gutartige und bösartige Tumoren der Mamma	21.1.8.5	20.55	
N61 Entzündliche Krankheiten der Mamma [Brustdrüse]	Mastitis	21.1.6.27		
N62 Hypertrophie der Mamma [Brustdrüse]	Gynäkomastie	21.1.3.16		
**N70-N77 Entzündliche Krankheiten der weiblichen Beckenorgane**				
N70 Salpingitis und Oophoritis		21.1.6.21		
N71 Entzündliche Krankheit des Uterus, ausgenommen der Zervix		21.1.6.21		
N72 Entzündliche Krankheit der Cervix uteri		21.1.6.21		
N73 Sonstige entzündliche Krankheiten im weiblichen Becken		21.1.6.21		
N75 Krankheiten der Bartholin-Drüsen		x		
N76 Sonstige entzündliche Krankheit der Vagina und Vulva	bakterielle Vaginose	20.38, 20.52	21.1.6.17, 21.1.6.23	
**N80-N98 Nichtentzündliche Krankheiten des weiblichen Genitaltraktes**				
N80 Endometriose		21.1.6.19		
N81 Genitalprolaps bei der Frau	Descensus uteri, Lageveränderungen des weiblichen Genitaltraktes (Descensus uteri, vaginae)	21.1.6.30		
N83 Nichtentzündliche Krankheiten des Ovars, der Tuba uterina und des Lig. Latum uteri	Primäre und sekundäre Ovarialinsuffizienz, Ovarialtorsion, Aufbrechen von Adnextumor, Eierstockzyste	21.1.3.24, 21.1.6.20, 21.1.6.22		
N85 Sonstige nichtentzündliche Krankheiten des Uterus, ausgenommen der Zervix		21.1.6		
N86 Erosion und Ektropium der Cervix uteri		x		
N87 Dysplasie der Cervix uteri		21.1.6.26		
N89 Sonstige nichtentzündliche Krankheiten der Vagina		x		
N90 Sonstige nichtentzündliche Krankheiten der Vulva und des Perineums	Schwellung im Bereich der Vulva, akute Schmerzen	21.1.6.17		
N91 Ausgebliebene, zu schwache oder zu seltene Menstruation	Störungen des ovariellen	20.1	21.1.6.28	
N92 Zu starke, zu häufige oder unregelmäßige Menstruation	sowie des endometrialen	20.1	21.1.6.28	
N94 Schmerz und andere Zustände im Zusammenhang mit den weiblichen Genitalorganen und dem Menstruationszyklus	Störungen des ovariellen sowie des endometrialen Zyklus und assoziierte Erkrankungen (Prämenstruelle Störungen,Hypermenorrhoe,Amenorrhoe,Oligomenorrhoe,Dysmenorrhoe), Schwellung im Bereich der Vulva, akute Schmerzen	20.1	21.1.6.17	21.1.6.28
N95 Klimakterische Störungen	Menopause, Klimakterium	21.1.6.29	20.1	
N97 Sterilität der Frau		21.1.6.38		
N98 Komplikationen im Zusammenhang mit künstlicher Befruchtung	Ovarielles Hyperstimulationssyndrom (OHSS, schwere Form)	18.3.5	21.1.3.15	
**000-008 Schwangerschaft mit abortivem Ausgang**				
000 Extrauteringravidität	Ektope Schwangerschaft	21.1.11.1		
001 Blasenmole		x		
003 Spontanabort		x		
**010-016 Ödeme, Proteinurie und Hypertonie während der Schwangerschaft, der Geburt und des Wochenbettes**	Eklampsie	21.1.11.5	21.1.11.10	21.1.11.15
014 Gestationshypertonie [schwangerschaftsinduziert] mit bedeutsamer Proteiunurie	Gestosen (Präeklampsie, Eklampsie, HELLP etc.)	21.1.11.5		
015 Eklampsie		21.1.11.5	21.1.11.15	
**020-029 Sonstige Krankheiten der Mutter, die vorwiegend mit der Schwangerschaft verbunden sind**				
020 Blutung in der Frühschwangerschaft		x		
021 Übermäßiges Erbrechen während der Schwangerschaft		x		
024 Diabetes mellitus in der Schwangerschaft	Gestationsdiabetes	21.1.11.6		
026 Betreuung der Mutter bei sonstigen Zuständen, die vorwiegend mit der Schwangerschaft verbunden sind		x		
**030-048 Betreuung der Mutter im Hinblick auf den Feten und die Amnionhöhle sowie mögiche Entbindungskomplitationen**	Nabelschnurkomplikationen, z.B. Vorliegen, Vorfall, Umschlingung, Knoten, Erkrankungen der Eihäte und des Fruchtwassers, z.B. vorzeitiger Blasensprung, Amnioninfekt, Poly-,Oligohydramnion	21.1.11.3	21.1.11.4	

**060-075 Komplikationen bei Wehentätigkeit und Entbindung**	Regelwidriger Geburtsmechanismus durch Lageanomalien, Haltungsanomalien, Einstellungsanomalien, Schulterdystokie	21.1.11.2	21.1.11.13	
**085-092 Komplikationen, die vorwiegend im Wochenbett auftreten**				
085 Puerperalfieber	Komplikationen im Wochenbett (Infektion, Anämie, Rückbildungsstörung, psychische Störungen etc.)	21.1.11.10		
088 EmboUe während der Gestationsperiode	Komplikationen im Wochenbett (Infektion, Anämie, Rückbildungsstörung, psychische Störungen etc.), Fruchtwasserembolie	21.1.11.10	21.1.11.14	
091 Infektionen der Mamma [Brustdrüse] im Zusammenhang mit der Gestation	Komplikationen im Wochenbett (Infektion, Anämie, Rückbildungsstörung, psychische Störungen etc.)	21.1.11.10		
**095-099 Sonstige Krankheitszustände während der Gestationsperiode, die anderenorts nicht klassifiziert sind**	Intrauterine Infektionen (TORCH)	21.1.11.20		
**P00-P04 Schädigung des Feten und Neugeborenen durch mütterliche Faktoren und durch Komplikationen bei Schwangerschaft, Wehentätigkeit und Entbundugn**	Regelwidriger Geburtsmechanismus, Nabelschnurkomplikationen, Substanzabusus, Regelwidrigkeiten der Plazenta, Fetomaternale Inkompatibilität	21.1.11.2, 21.1.11.3	21.1.11.8, 21.1.11.29	21.1.11.7
**P05-P08 Störungen im Zusammenhang mit der Schwangerschaftsdauer und dem fetalen Wachstum**				
P05 Intrauterine Mangelentwicklung und fetale Mangelernährung	Wachstumsstörungen	21.1.11.17		
P08 Störungen im Zusammenhang mit kurzer Schwangerschaftsdauer und niedrigem Geburtsgewicht, anderenorts nicht klassifiziert	Störungen des Geburtszeitpunktes (Frühgeburtlihckeit, Übertragung), Frühgeborenen-Retinopathie	21.1.11.18	21.1.11.35	
**P10-P15 Geburtstrauma**	Geburtsverletzungen der Mutter (z.B. Dammriss, Episiotomie)	21.1.11.9	21.1.11.15	
**P20-P29 Krankheiten des Atmungs- und Herz-Kreislaufsystems, die für die Perinatalperiode spezifisch sind**	Ductus botalli, ARDS, bronchopulmonale Dysplasie, Acute Respiratory Distress Syndrome (ARDS), perinatale Asphyxie, IRDS, neonatale Pneumonie und respiratorische Anpassungsstörungen (respiratory distress in infants) inkl. Mekoniumaspiration	21.1.4.32, 21.1.11.16	21.1.11.22, 21.1.11.26	21.1.11.27
**P35-P39 Infektionen, die für die Perinatalperiode spezifisch sind**	intrauterine Infektionen, neonatale Sepsis	21.1.11.20	21.1.11.19	
**P50-P61 Hämorrhagische und hämatologische Krankheiten beim Feten und Neugeborenen**	Vitamin-K-Mangelblutung	21.1.11.28		
P53 Hämorrhagische Krankheit beim Feten und Neugeborenen	Fetomaternale Inkompatibilität (M. haemolyticus neonatorum, fetale,noenatale)	21.1.11.28	21.1.11.29	
P55 Hämolytische Krankheit beim Feten und Neugeborenen		21.1.11.29		
P57 Kernikterus		16.3.26		
P59 Neugeborenenikterus durch sonstige und nicht näher bezeichnete Ursachen		16.3.26		
**P70-P74 Transitorische endokrine und Stoffwechselstörungen, die für den Feten und das Neugeborene spezifisch sind**				
P70 Transitorische Störungen des Kohlenhydratstoffwechsels, die für den Feten und das Neugeborene spezifisch sind		x		
P74 Sonstige transitorische Störungen des Elektrolythaushaltes und des Stoffwechsels beim Neugeborenen		x		
**P75-P78 Krankheiten des Verdauungssystems beim Feten und neugeborenen**				
P75 Mekoniumileus		21.1.11.32		
P77 Enterocolitis necroticans beim Feten und Neugeborenen	Nekrotisierende Enterokolitis bei Frühgeborenen	21.1.11.34		
**P90-P96 Sonstige Störungen, die ihren Ursprung in der Perinatalperiode haben**				

P90 Krämpfe beim Neugeborenen	neonatale Hirnblutung, zerebrale Fehlbildungen	21.1.11.39	21.1.11.37	
P91 Sonstige zerebrale Störungen beim Neugeborenen	Periventrikuläre Leukomalazie, Neonatale Hirnblutung	21.1.11.36, 21.1.11.37	21.1.11.38, '21.1.11.39	
P92 Ernährungsprobleme beim Neugeborenen		x		
**Q00-Q07 Angeborene Fehlbildungen des Nervensystems**		21.1.11.36	21.1.11.38	21.1.11.39
Q05 Spina bifida	Neuralrohrdefekte, Spina Bifida	21.1.11.38		
Q07 Sonstige angeborene Fehlbildungen des Nervensystems	Neuralrohrdefekte, Spina Bifida, Hydrocephalus und zerebrale Fehlbildungen	21.1.11.36	21.1.11.38	21.1.11.39
**Q10-Q18 Angeborene Fehlbildungen des Auges, des Ohres, des Gesichtes und des Halses**		21.1.11.24	20.3	
**Q20-Q28 Angeborene Fehlbildungen des Kreislaufsystems**	Kongenitale Herzfehler, Persistierender Ductus arteriosus des Frühgeborenen	21.1.11.21, 21.1.11.22		
**Q30-Q34 Angeborene Fehlbildungen des Atmungssystems**	Fehlbildungen des Respirationstraktes: Choanalatresie	21.1.11.25, 21.1.11.27		
Q30 Angeborene Fehlbildungen der Nase		20.3	21.1.11.25	
Q33 Angeborene Fehlbildungen der Lunge		21.1.11.25	21.1.11.27	
**Q35-Q37 Lippen-, Kiefer- und Gaumenspalte**	Lippen-Kiefer-Gaumenspalte, Gesichtsfehlbildung	21.1.11.24		
**Q38-Q45 Sonstige angeborene Fehlbildungen des Verdauungssystems**	Atresien und Fehlbildungen des Gastrointestinaltrakts bei Feten und Neugeborenen (Speiseröhren-, Darm-, Gallengangs-, Anal-Atresie; Volvolus, Malrotation)	21.1.11.31		
Q39 Angeborene Fehlbildungen des Ösophagus	Atresien und Fehlbildungen des Gastrointestinaltrakts bei Feten und Neugeborenen (Speiseröhren-, Darm-, Gallengangs-, Anal-Atresie; Volvolus, Malrotation)	21.1.11.31		
Q40 Sonstige angeborene Fehlbildungen des oberen Verdauungstraktes	Angeborene Pylorusstenose	21.1.11.31	21.1.7.34	
Q41 Angeborene(s) Fehlen, Atresie und Stenose des Dünndarmes	Atresien und Fehlbildungen des Gastrointestinaltrakts bei Feten und Neugeborenen (Speiseröhren-, Darm-, Gallengangs-, Anal-Atresie; Volvolus, Malrotation)	21.1.11.31		
Q43 Sonstige angeborene Fehlbildungen des Darmes	Dünndarm- und Dickdarmdivertikel inkl. Komplikationen, Malrotation, Volvulus, Kongenitales Megakolon	21.1.7.17	21.1.11.31	21.1.11.33
Q44 Angeborene Fehlbildungen der Gallenblase, der Gallengänge und der Leber	Atresien und Fehlbildungen des Gastrointestinaltrakts bei Feten und Neugeborenen (Speiseröhren-, Darm-, Gallengangs-, Anal-Atresie; Volvolus, Malrotation)	21.1.11.31		
**Q50-Q56 Angeborene Fehlbildungen der Genitalorgane**		21.1.11.30	20.3	
Q51 Angeborene Fehlbildungen des Uterus und der Cervix uteri		20.3		
Q52 Sonstige angeborene Fehlbildungen der weiblichen Genitalorgane		20.3		
Q53 Nondescensus testis	Hodenhochstand	21.1.6.8		
Q54 Hypospadie		21.1.11.30		
Q55 Sonstige angeborene Fehlbildungen der männlichen Genitalorgane		21.1.6.8, 21.1.6.10	21.1.6.11, 21.1.11.30	
**Q60-Q64 Angeborene Fehlbildungen des Harnsystems**	Zystische Nierenkrankheit, Renale und urogenitale Fehlbildung (z.B. Hufeisenniere, Hypospadie, Blasenekstrophie, Harnröhrenklappen, Hymenalatresie)	21.1.6.16	21.1.11.30	
**Q65-Q79 Angeborene Fehlbildungen und Deformitäten des Muskel-Skelett-Systems**				
Q65 Angeborene Deformitäten der Hüfte	Hüftgelenksdysplasie	21.1.2.36		
Q66 Angeborene Deformitäten der Füße	Angeborene Fußdeformitäten, z.B. Klumpfuß, Sichelfuß	21.1.11.23		

Q67 Angeborene Muskel-Skelett-Deformitäten des Kopfes, des Gesichtes, der Wirbelsäule und des Thorax	Skoliosen und sonstige Wirbelsäulendeformitäten	21.1.2.13	21.1.11.24	
Q71 Reduktionsdefekte der oberen Extremität		x		
Q72 Reduktionsdefekte der unteren Extremität		x		
Q73 Reduktionsdefekte nicht näher bezeichneter Extremität(en)		x		
Q75 Sonstige angeborene Fehlbildungen der Schädel- und Gesichtsschädelknochen	Achondroplasie, Osteogenesis imperfecta, Kraniosynostose	20.3	21.1.2.46	21.1.11.24
Q76 Angeborene Fehlbildungen der Wirbelsäule und des knöchernen Thorax		21.1.2.13		
Q78 Sonstige Osteochondrodysplasien	Achondroplasie, Osteogenesis imperfecta, Kraniosynostose	21.1.2.11	21.1.2.46	
Q79 Angeborene Fehlbildungen des Muskel-Skelett-Systems, anderenorts nicht klassifiziert	Kongenitale Hernien und Defekte von Zwerchfell und Bauchwand, Osteogenesis imperfecta	21.1.2.33	21.1.2.37	
**Q80-Q89 Sonstige angeborene Fehlbildungen**				
Q80 Ichthyosis congenita		x		
Q82 Sonstige angeborene Fehlbildungen der Haut		x		
Q85 Phakomatosen, anderenorts nicht klassifiziert	Neurofibromatose	21.1.10.38		
Q86 Angeborene Fehlbildungssyndrome durch bekannte äußere Ursachen, anderenorts nicht klassifiziert	Fehlbildungen, Folgen von Substanzabusus (z.B. Nikotin, Alkohol) und von Gewalterfahrung und -erleben in der Schwangerschaft	20.3	21.1.11.7	
Q87 Sonstige näher bezeichnete angeborene Fehlbildungssyndrome mit beteiligung mehrerer Systeme	Fehlbildungen, Marfan-Syndrom	20.3	21.1.2.5	
**Q90-Q99 Chromosomenanomalien, anderenorts nicht klassifiziert**	Chromosomenanomalien (Trisomien, Klinefelter-Syndrom, Turner-Syndrom)	21.1.11.21		
**R10-R19 5ymptome, die das Verdauungssystem und das Abdomen betreffen**				
R10 Bauch- und Beckenschmerzen		20.15		
R11 Übelkeit und Erbrechen		20.110		
**R50-R69 Allgemeinsymptome**		20.63		
R55 Synkope und Kollaps		21.1.1.16		
R56 Krämpfe, anderenorts nicht klassifiziert	Crampi, Muskelkrämpfe	20.58	21.1.10.19	21.1.10.20
R64 Kachexie	Unterernährung (Kachexie, Anorexie, Sarkopenie)	20.111	21.1.3.38	
**R95-R99 Ungenau bezeichnete und unbekannte Todesursachen**				
R95 Plötzlicher Kindstod		21.1.4.25		
**S00-S09 Verletzungen des Kopfes**	Weichgewebsverletzung des Gesichts, Schädelfrakturen, Schädel-Hirn-Trauma	20.88, 21.1.2.6	21.1.2.45	21.1.10.2
**S10-S19 Verletzungen des Halses**		x		
**S20-S29 Verletzungen des Thorax**	Thoraxtrauma	21.1.4.1		
**S30-S39 Verletzungen des Abdomens, der Lumbosakralgegend, der Lendenwirbelsäule und des Beckens**	Beckentrauma und begleitende Organverletzungen, Verletzung parenchymatöser abdomineller Organe, z.B. Milz, Leber, Pankreas, Niere	21.1.2.1	21.1.7.8	
**S40-S49 Verletzungen der Schulter und des Oberarmes**	Claviculafraktur, Verletzungdes AC-Gelenks, Humerusfrakturen	21.1.2.15, 21.1.2.16	21.1.2.17, 21.1.2.18	21.1.2.21
**S50-S59 Verletzungen des Ellenbogens und des Unterarmes**	Distale Radiusfraktur und andere Verletzungen des Unterarms	21.1.2.23	21.1.2.18	21.1.2.19
**S60-S69 Verletzungen des Handgelenkes und der Hand**	Frakturen und Bandverletzungen der Hand und des Handgelenks, Erkrankungen desNagels, Fingerkuppenverletzung	21.1.2.24	21.1.8.8	
**S70-S79 Verletzungen der Hüfte und des Oberschenkels**	Femurfrakturen	21.1.2.1	21.1.2.20	
**S80-S89 Verletzungen des Knies und des Unterschenkels**	Frakturen und Bandverletzungen der Hand und des Handgelenks	21.1.2.22	21.1.2.23	21.1.2.32
**S90-S99 Verletzungen der Knöchelregion und des Fußes**		21.1.2.23		
**T00-T07 Verletzungen mit Beteiligung mehrerer Körperregionen**		x		

**T08-T14 Verletzungen nicht näher bezeichneter Teile des Rumpfes, der Extremitätten oder anderer Körperregionen**	Verletzungen der Wirbelsäule, Wirbelfrakturen, Thoraxtrauma	21.1.2.14, 21.1.4.1		
**T15-T19 Folgen des Eindringens eines Fremdkörpers durch eine natürliche Körperöffnung**	Fremdkörperaspiration und -ingestion	21.1.4.2	21.1.9.15	
**T20-T32 Verbrennungen oder Verätzungen**	Verbrennung, Verbrühung, Unterkühlung, Erfrierung	20.114	21.1.8.1	
**133-135 Erfrierungen**	Verbrennung, Verbrühung, Unterkühlung, Erfrierung	21.1.8.1		
**T36-T50 Vergiftungen durch Arzneimittel, Drogen und biologisch aktive Substanzen**	Intoxikation	21.1.10.65	20.51	
**T51-T65 Toxische Wirkungen von vorwiegend nicht medizinisch verwendeten Substanzen**	Intoxikationen durch Alkohol, Drogen, Medikamente und andere Toxine	21.1.10.65	20.51	
**T66-T78 Sonstige und nicht näher bezeichnete Schäden durch äußere Ursachen**		x		
T67 Schäden durch Hitze und Sonnenlicht		x		
T68 Hypothermie	Unterkühlung	21.1.8.1		
T69 Sonstige Schäden durch niedrige Temperatur	Erfrierung	21.1.8.1		
T71 Erstickung		x		
T74 Missbrauch von Personen	Misshandlung, Kindesmusshandlung, Sexueller Missbrauch	20.41, 20.54	21.1.2.35, 21.1.6.32	
T75 Schäden durch sonstige äußere Ursachen		x		
T78 Unerwünschte Nebenwirkungen, anderenorts nicht klassifiziert	Maligne Hyperthermie	20.51	20.59	21.1.2.8
T79 Bestimmte Frühkomplikationen eines Traumas, anderenorts nicht klassifiziert	Kompartmentsyndrom der Extremitäten	21.1.2.4		
**T80-T88 Komplikationen bei chirurgischen Eingriffen und medizinscher Behandlung, anderenorts nicht klassifiziert**		x		
**U00-U49 Vorläufige Zuordnungen für Krankheiten mit unklarer Ätiologie**		x		
U04 Schweres akutes respiratorisches Syndrom [SARS]		x		
**U80-U85 Infektionserreger mit Resistenzen gegen bestimmte Antibiotika oder Chemotherapeutika**		x		
U80 Erreger mit bestimmten Antibiotikaresistenzen, die besondere therapeutische oder hygienische Maßnahmen erfordern		x		
U82 Mykobakterien mit Resistenz gegen Antituberkulotika (Erstrangmedikamente)		x		
**V01-X59 Unfälle**		x		
**X60-X84 Vorsätzliche Selbstbeschädigung**		20.71		
**X85-Y09 Tätlicher Angriff**		20.71		
**Z00-Z013 Personen, die das Gesundheitswesen zur Untersuchung und Abklärung in Anspruch nehmen**				
Z00 Allgemeinuntersuchung und Abklärung bei Personen ohne Beschwerden oder angegebene Diagnose		20.85	21.1.10.56	
Z08 Nachuntersuchung nach Behandlung wegen bösartiger Neubildung		20.76		
Z09 Nachuntersuchung nach Behandlung wegen anderer Krankheitszustände außer bösartigen Neubildungen		20.76		
**Z30-Z39 Personen, die das Gesundheitswesen im Zusammenhang mit Problemen der Reproduktion in Anspruch nehmen**		x		
Z30 Kontrazeptive Maßnahmen		x		
**Z70-Z76 Personen, die das Gesundheitswesen aus sonstigen Gründen in Anspruch nehemen**		x		
Z71 Personen, die das Gesundheitswesen zum Zwecke anderer Beratung oder ärztlicher Konsultation in Anspruch nehmen, anderenorts nicht klassifiziert		x		

MERLIN
Medical Education Research -
Lehrforschung im Netz BW

Universität Tübingen

Kompetenzzentrum für Hochschuldidaktik in Medizin Baden-Württemberg
Medizinische Fakultät
Elfriede-Aulhorn-Straße 10 · 72076 Tübingen
Telefon +49 7071 29-77974 · Fax +49 7071 29-5218
www.medizin-bw.de
www.medidaktik.de
